# Novel perfluoropolyalkylethers monomers: synthesis and photo-induced cationic polymerization

**DOI:** 10.1007/s00396-021-04838-1

**Published:** 2021-04-25

**Authors:** Giuseppe Trusiano, Alessandra Vitale, Jason Pulfer, Josiah Newton, Christine Joly-Duhamel, Chadron M. Friesen, Roberta Bongiovanni

**Affiliations:** 1 Department of Applied Science and Technology, Politecnico di Torino, Corso Duca degli Abruzzi 24, 10129 Turin, Italy; 2 Department of Chemistry, Trinity Western University, 22500 University Drive, Langley City, BC V2Y 1Y1 Canada; 3 Department of Chemistry, Simon Fraser University, 8888 University Drive, Burnaby, BC V5A 1S6 Canada; 4 University of Montpellier, Institut Charles Gerhardt Montpellier, CNRS, ENSCM, Cedex 5, 34095 Montpellier, France

**Keywords:** Functionalized perfluoropolyalkylethers, Photopolymerization, Fluoropolymers

## Abstract

Several difunctional oligomers were synthesized by functionalizing perfluoropolyalkylether (PFPAE) chains with different vinyl ethers and epoxides end-groups. Due to their innate synthetic challenges and demanding purification protocols, the PFPAE derivatives were obtained in low yield and with an average functionality lower than 2. However, the functionalized PFPAE oligomers were successful in being used in photo-induced cationic polymerization processes, obtaining transparent and soft films. The influences of the fluorinated chains, and various end-groups on the photopolymerization process were investigated, as well their chemical stability, thermal degradation, and surface properties.

**Supplementary Information:**

The online version contains supplementary material available at 10.1007/s00396-021-04838-1.

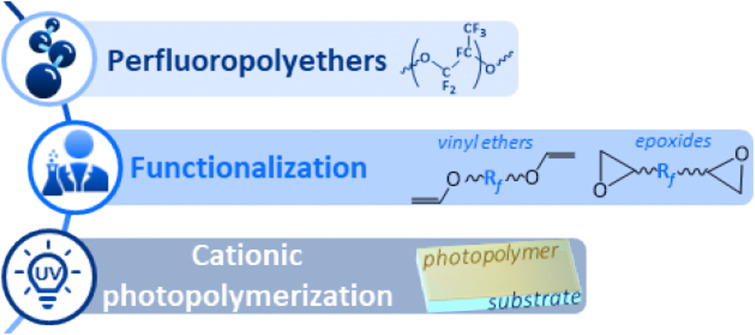

## Introduction

Cationic photo-induced polymerization of reactive monomers, such as epoxides and vinyl ethers, is a process that has an established history [1, 2]. At the moment, it is commonly used in several industrial fields such as inks, coatings, adhesives, and microelectronic devices thanks to the significant benefits of the process (lack of solvents and of oxygen inhibition, low toxicity, fast, and room temperature operations) and to the important advantages shown by the obtained cured materials (low shrinkage, good mechanical, and adhesion properties).

Having established industrial relevance, cationic photoinduced polymerization utility can be further refined by gaining photocontrol over chain growth [3], using a variety of catalysts and chain-transfer agents [4]. Spatiotemporal control is easily obtained by light and allows for precise regulation of polymer structure, function, and architectural design. Recently, radical photo-controlled (co)polymerization of fluoro-olefins have been reported [5], allowing smooth transformation of CTFE at ambient pressure and room temperature.

In addition, another research line in cationic photopolymerization is addressed by improving performances of the products with fluorinated building blocks. Beyond fluoroalkylic monomers, perfluoropolyalkylethers (PFPAEs) are interesting structures: they represent a special class of fluoropolymers based on repetitive units such as –(CF_2_O)–, –(CF_2_CF_2_O)–, –(CF_2_CF_2_CF_2_O)–, and –(CF(CF_3_)CF_2_O)–, and end-groups like CF_3_O–, C_2_F_5_O–, C_3_F_7_O–, depending on the synthesis route [6, 7]. PFPAEs show outstanding properties: high thermal and chemical inertness, low glass transition temperature, low adhesion and friction, low refractive index, very low surface tension, and good protection against corrosion, environmental pollution, and weather aggression [6–13].

Moreover, data has shown that PFPAEs are non-toxic [14–22], even when the fluorinated chains are short: therefore, they are a safe alternative to the fluoropolymers currently banned in many countries.

For these reasons, PFPAEs have previously been explored containing functionalization with acrylic [23, 24], methacrylic [10, 25–28], or maleimidic [29, 30] end-groups to be used in radical photopolymerization. It is well known that this process suffers from oxygen inhibition; this drawback is even more severe for fluorinated products, as they show a high oxygen solubility. Synthesizing fluorinated monomers with functionalities suitable for cationic photoprocesses is therefore attractive. However, up to now few structure are available and have been investigated only as comonomers [31–38]. The synthesis of functional PFPAE derivatives is a demanding target to achieve: PFPAEs are extremely non-polar substances, poor nucleophile, and insoluble in almost all the organic solvents, except for few fluorinated solvents [29, 31, 32].

In this work, several difunctional oligomers were synthesized by functionalizing PFPAEs chains with vinyl ethers and epoxides end-groups: their general structure is RG-S-PFPAE-S-RG where RG represents the reactive function and S is a non-fluorinated alkyl spacer.

The functionalized PFPAE oligomers were previously used in copolymerization with hydrogenated vinylethers and epoxides: here, after describing their synthesis, the molecular structures and main physico-chemical properties by multinuclear NMR, their reactivity in photo-induced cationic polymerization was studied, showing the influence of the fluorinated chain, and of the different length of the hydrogenated spacers by means of FTIR spectroscopy and photo DSC. The thermal and the surface properties of the obtained polymers are also presented.

## Materials and methods

### Synthesis of the fluorinated vinyl ethers and epoxides

#### Materials

Oligo(PFPAE) diacyl fluoride was synthesized via the anionic ring-opening polymerization of hexafluoropropoxide (HFPO, generously supplied by Chemours™, USA), following the procedure described elsewhere [39–42]. Different batches of the synthesized oligo(PFPAE) diacyl fluoride were mixed to be used for the syntheses of vinyl ether derivatives. Oligo(PFPAE) dicarboxylic acid, obtained by hydrolysis of the acyl fluoride, was used for the synthesis of the epoxy derivatives.

The chemicals employed for the functionalization of the oligo(PFPAE) are the following: 1,1,1,3,3-pentafluorobutane (PFB) was purchased from Alfa Aesar (Ward Hill, Massachusetts, USA). Epibromohydrin, ethylene glycol vinyl ether (EGVE), 1,4-Butylene glycol divinyl ether (BGVE), di(ethylene glycol) vinyl ether (DEGVE), silver oxide, 4-dimethylaminopyridine (DMAP), diethyl ether (Et_2_O), ethyl acetate (EtOAc), Triethylamine (Et_3_N), acetonitrile (MeCN), and dichloromethane (DCM), and all other chemicals were purchased from Sigma Aldrich (Canada). PFB, Et_3_N, MeCN, and DCM were dried for 48 h over preconditioned 3 Å molecular sieves (20% w v^−1^).

#### Monomer characterization

The structure of the synthesized reagents and PFPAE monomers were determined by NMR spectroscopy at room temperature (25 °C). NMR spectra were recorded on a Bruker AVANCE III 400 MHz spectrometer instruments using deuterated chloroform-*d*, dichloromethane-*d*_*2*_, and benzene-*d*_*6*_ capillaries as internal references for the fluorinated products. The experimental conditions were accomplished by using MestReNova 12.00 operating at 400.13 (^1^H), 376.46 (^19^F), 100.62 (^13^C) MHz. The letters *s*, *d*, *t*, *q*, *quint*, *sext*, and *spt* stand for singlet, doublet, triplet, quartet, quintuplet, sextet, and septuplet, respectively.

GC-MS spectra were obtained by using an Agilent Technologies 6890N gas-chromatographer (GC), coupled with an Agilent Technologies 7638B series injector. An Agilent Technologies 5975B inert mass spectrometer (MS) was employed with 70 eV electron impact (EI) as the mode of ionization. The GC was equipped with a Zebron ZB-5ms column, 30 m × 0.18 mm id, 0.18 mm df. The detector and the injector temperatures were 200 °C and 280 °C, respectively. The temperature program started from 50 °C with a 2 min hold then the heating rate was 25 °C/min until reaching 250 °C and holding at 250 °C for 2 min. Total pressure was 108 kPa, total flow was 25.9 mL/min, column flow 0.74 mL min^−1^, purge flow 3 mL min^−1^, linear velocity 38.2 cm s^−1^, and a split injection of 30:1. The samples were diluted in methoxyperfluorobutane (3M’s Novec™ HFE-7100) in a GC vial. The written fragments correspond to at least 10% of the highest signal.

#### General synthesis of PFPAE-vinyl ethers

A solution of PFPAE diacyl fluoride (1 equiv., *M*_*n*_ = 4450 g mol^−1^) in 5 mL of dried PFB was added to a 100 mL 3-necked round-bottomed flask and cooled at 0 °C. A mixture of triethylamine (2.5 equiv.), DMAP (6 wt.%), and alkyl glycol vinyl ether (5 equiv.) in 25 mL of dry 40:60 DCM:PFB solution was added dropwise to the PFPAE diacyl fluoride/PFB mixture, at 0 °C, under continuous stirring. After 30 min, the ice bath was removed, and the reaction mixture was let to stir at room temperature during 24 h. The reaction progress was monitored by thin layer chromatography (TLC): powdered iodine was used as TLC stain and a 5:95 EtOAc/pentane solution as eluent. The conversion of the diacyl fluoride into the corresponding vinyl ether was checked by the appearance of an orange-brown spot on light yellow background, located between the baseline of the TLC plate and the eluent front). The reaction was completed after 24 h, as suggested by gas chromatography–mass spectrometry (GC–MS). Then, the reaction mixture was purified by flash chromatography (5:95 EtOAc/pentane solution used as eluent). After vacuum purging, a transparent oil was obtained as pure product (S1 in the Supporting Information).

#### Synthesis of perfluoropolyalkylether–ethylene glycol vinyl ether (PFPAE-EGVE)

PFPAE diacyl fluoride (*M*_*n*_ = 4450 g mol^−1^, 1 mmol, 2.21 mL), Et_3_N (2.5 equiv., 2.5 mmol, 0.35 mL), DMAP (6 wt.%, 0.6 mmol, 73.3 mg), ethylene glycol vinyl ether (5 equiv., 5 mmol, 0.45 mL). Isolated yield: 18%.

^1^H-NMR (400 MHz, benzene-*d*_*6*_ capillary, 25 °C, S2 in the Supporting Information):

δ = 6.66 (*dd*, -OC***H***CH_2_, ^3^*J*_*cis*_ = 14.4 Hz, ^3^*J*_*trans*_ = 6.8 Hz, 1H), 4.81 (*d*, -C(O)OC***H***_***2***_CH_2_-, *J* = 3.7 Hz, 2H), 4.40 (*d*, -OCH*=*C***H***_***trans***_H_cis_, ^3^*J*_cis_ *=* 15.3 Hz, 1H), 4.28 (*d,* -OCH*=*CH_trans_***H***_***cis***_, ^3^*J*_cis_ = 6.5 Hz, 1H), 4.16 (*s*, -C(O)OCH_2_C***H***_***2***_-, *J* = 5.3 Hz, 2H).

^13^C-NMR (101 MHz, benzene-*d*_*6*_ capillary, 25 °C, S3 in the Supporting Information):

δ = 158.53 (*s,* -***C***=O-), 151.14 (*s*, -O***C***HCH_2_), 122.49-99.17 (*m,* carbons of repeat unit), 86.92 (*s,* -OCH***C***H_2_), 66.16 (*s,* -***C***H_2_OCHCH_2_), 64.35 (*s*, -***C***H_2_CH_2_OCHCH_2_).

^19^F-NMR (376.5 MHz, benzene-*d*_*6*_ capillary, 25 °C, S4 in the Supporting Information):

δ = − 145.15 (*q*, C***F***(CF_3_) of repeat unit, mono and difunctional), − 131.99 (*ω* C***F***(CF_3_), mono and difunctional), − 130.23 (*s*, α C***F***_***2,***_ monofunctional), − 125.67 (*s*, - CF_2_C***F***_***2***_CF_2_O-_***,***_ difunctional), − 122.66 (*s*, - C***F***_***2***_CF_2_CF_2_O-_***,***_ difunctional), from − 85.64 to − 78.92 (CF_3_ and CF_2_ of repeat unit). The molecular weight of the product was calculated from the ^19^F-NMR spectra, as in S5 of the Supporting Information.

GC–MS (EI) fragmentation (S6 in the Supporting Information):

m/z = OCHCH_2_^+^ (43 m/z), CH_2_OCHCH_2_^+^ (57 m/z), CF_3_^+^ (69 m/z), CH_2_CH_2_OCHCH_2_^+^ (71 m/z), OCH_2_CH_2_OCHCH_2_^+^ (87 m/z), C_2_F_4_^+^ (100 m/z), C_2_F_5_^+^ (119 m/z), C_3_F_5_O^+^ (147 m/z), C_3_F_6_^+^ (150 m/z), C_3_F_7_^+^ (169 m/z), CF(CF_3_)C(O)OCH_2_CH_2_OCHCH_2_^+^ (215 m/z).

#### Synthesis of perfluoropolyalkylether–butylene glycol vinyl ether (PFPAE-BGVE)

PFPAE diacyl fluoride (*M*_*n*_ = 4450 g mol^−1^, 1 mmol, 2.21 mL), Et_3_N (2.5 equiv., 2.5 mmol, 0.35 mL), DMAP (6 wt.%, 0.6 mmol, 73.3 mg), 1,4 butylene glycol vinyl ether (5 equiv., 5 mmol, 0.62 mL). Isolated yield: 78%.

^1^H-NMR (400 MHz, benzene-*d*_*6*_ capillary, 25 °C, S7 in the Supporting Information):

δ = 6.65 (*dd*, -OC**H**CH_2_, ^3^*J*_*cis*_ = 14.4 Hz, ^3^*J*_*trans*_ = 6.6 Hz, 1H), 4.67 (*s*, -C(O)OC***H***_***2***_CH_2_-, 2H), 4.35 (*d,* ^3^*J*_cis_ = 17.3 Hz, -OCH*=*C***H***_***trans***_H_cis_, 1H), 4.19 (*d,* ^3^*J*_cis_ *=* 11.1 Hz, -OCH*=*CH_trans_***H***_***cis***_, 1H), 3.93 (*s*, -C(O)OCH_2_CH_2_CH_2_C***H***_***2***_-, 2H), 2.14 (*s*, -C(O)OCH_2_C***H***_***2***_CH_2_CH_2_-, 2H), 2.03 (*s*, -C(O)OCH_2_CH_2_C***H***_***2***_CH_2_-, 2H).

^13^C-NMR (101 MHz, benzene-*d*_*6*_ capillary, 25 °C, S8 in the Supporting Information):

δ = 158.46 (*d,* -***C***=O-), 151.74 (*s*, -O***C***HCH_2_), 122.47-99.58 (*m,* carbons of repeat unit), 86.19 (*s,* -OCH***C***H_2_), 68.31 (*s,* -***C***H_2_OCHCH_2_), 66.34 (*s*, -***C***H_2_CH_2_CH_2_CH_2_OCHCH_2_), 25.57 (*s*, -CH_2_CH_2_***C***H_2_CH_2_OCHCH_2_), 25.44 (*s*, -CH_2_***C***H_2_CH_2_CH_2_OCHCH_2_).

^19^F-NMR (376.5 MHz, benzene-*d*_*6*_ capillary, 25 °C, S9 in the Supporting Information):

δ = − 145.22 (*q*, C***F***(CF_3_) of repeat unit, mono and difunctional), − 131.91 (*ω* C***F***(CF_3_), mono and difunctional), − 130.29 (*s*, α C***F***_***2,***_ monofunctional), − 125.73 (*s*, -CF_2_C***F***_***2***_CF_2_O-_***,***_ difunctional), − 122.72 (*s*, -C***F***_***2***_CF_2_CF_2_O-_***,***_ difunctional), from − 85.45 to − 79.30 (CF_3_ and CF_2_ of repeat unit). The molecular weight of the product was calculated from the ^19^F-NMR spectra, as in S10 of the Supporting Information.

GC–MS (EI) fragmentation (S11 in the Supporting Information):

m/z = OCHCH_2_^+^ (43 m/z), CH_2_OCHCH_2_^+^ (57 m/z), CF_3_^+^ (69 m/z), CH_2_CH_2_OCHCH_2_^+^ (71 m/z), CH_2_CH_2_CH_2_OCHCH_2_^+^ (85 m/z), CH_2_CH_2_CH_2_CH_2_OCHCH_2_^+^ (99 m/z), C_2_F_4_^+^ (100 m/z), OCH_2_CH_2_CH_2_CH_2_OCHCH_2_^+^ (115 m/z), C_2_F_5_^+^ (119 m/z), C_3_F_5_O^+^ (147 m/z), C_3_F_6_^+^ (150 m/z), C_3_F_7_^+^ (169 m/z), CF(CF_3_)C(O)OCH_2_CH_2_CH_2_CH_2_OCHCH_2_^+^ (243 m/z).

#### Synthesis of perfluoropolyalkylether–di-(ethylene glycol) vinyl ether (PFPAE-DEGVE)

PFPAE diacyl fluoride (*M*_*n*_ = 4450 g mol^−1^, 1 mmol, 2.21 mL), Et_3_N (2.5 equiv., 2.5 mmol, 0.35 mL), DMAP (6 wt.%, 0.6 mmol, 73.3 mg), diethylene glycol vinyl ether (5 equiv., 5 mmol, 0.67 mL). Isolated yield: 12%.

^1^H-NMR (400 MHz, benzene-*d*_*6*_ capillary, 25 °C, S12 in the Supporting Information):

δ = 6.64 (*dd*, -OC***H***CH_2_, ^3^*J*_*cis*_ = 14.1 Hz, ^3^*J*_*trans*_ = 7.3 Hz, 1H), 4.76 (*s*, -C(O)OC***H***_***2***_CH_2_O-, 2H), 4.39 (*d,* -OCH*=*C***H***_***trans***_H_cis_, ^3^*J*_cis_ = 14.1 Hz, 1H), 4.21 (*d*, -OCH*=*CH_trans_***H***_***cis***_, ^3^*J*_cis_ *=* 7.3 Hz, 1H), 4.03 (*s*, -C(O)OCH_2_C***H***_***2***_OC***H***_***2***_CH_2_O-, 4H), 3.95 (*s*, -OCH_2_C***H***_***2***_OCHCH_2_, 2H).

^13^C-NMR (101 MHz, benzene-*d*_*6*_ capillary, 25 ^o^C, S13 in the Supporting Information):

δ = 158.82 (*d*, -***C***=O-), 151.77 (*s*, -O***C***HCH_2_), 122.52-99.61 (*m*, carbons of repeat unit), 86.43 (*s*, -OCH***C***H_2_), 69.98 (*s*, *-****C***H_2_CH_2_OCHCH_2_), 68.59 (*s*, *-*C(O)OCH_2_***C***H_2_O-), 67.40 (*s*, -OCH_2_***C***H_2_OCHCH_2_), 67.32 (*s*, *-*C(O)O***C***H_2_CH_2_O-).

^19^F-NMR (376.5 MHz, benzene-*d*_*6*_ capillary, 25 °C, S14 in the Supporting Information):

δ = − 145.18 (*q*, C***F***(CF_3_) of repeat unit, mono and difunctional), − 131.96 (*ω* C***F***(CF_3_), mono and difunctional), − 130.32 (*s*, α C***F***_***2,***_ monofunctional), − 125.74 (*s*, - CF_2_C***F***_***2***_CF_2_O-_***,***_ difunctional), − 122.70 (*s*, -C***F***_***2***_CF_2_CF_2_O-_***,***_ difunctional), from − 85.48 to − 79.10 (CF_3_ and CF_2_ of repeat unit). The molecular weight of the product was calculated from the ^19^F-NMR spectra, as in S15 of the Supporting Information.

GC–MS (EI) fragmentation (S16 in the Supporting Information):

m/z = OCHCH_2_^+^ (43 m/z), CH_2_OCHCH_2_^+^ (57 m/z), CF_3_^+^ (69 m/z), CH_2_CH_2_OCHCH_2_^+^ (71 m/z), OCH_2_CH_2_OCHCH_2_^+^ (87 m/z), C_2_F_4_^+^ (100 m/z), CH_2_CH_2_OCH_2_CH_2_OCHCH_2_^+^ (115 m/z), C_2_F_5_^+^ (119 m/z), C_3_F_5_O^+^ (147 m/z), C_3_F_6_^+^ (150 m/z), C_3_F_7_^+^ (169 m/z), CF(CF_3_)C(O)OCH_2_CH_2_OCH_2_CH_2_OCHCH_2_^+^ (259 m/z).

#### General synthesis of the oxirane precursors

**Scheme 1:**
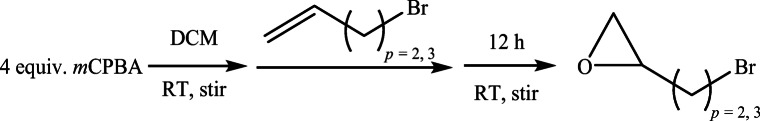
Synthesis of the bromo-alkyl-oxirane by epoxidation reaction of bromo-alkenes

The bromo-alkene was added to a solution of *m*CPBA in DCM (15 mL). The solution was allowed to stir overnight (Scheme 1). After mixing, the reaction mixture was cool down at 0 °C: the *m*CPBA/benzoic acid precipitate was filtered through a short column, and the solvent was removed under vacuum (×3). The mixture was then washed with 10% aqueous solution of Na_2_SO_4_ (×3), with a saturated solution of NaHCO_3_ (×3), and with a saturated aqueous solution of LiCl. The crude was dried over Na_2_SO_4_ and the solvent traces were then removed under vacuum, to afford the desired known compound as a colorless liquid.

#### Synthesis of 2-(2-bromoethyl)oxirane)

4-bromo-1-butene (0.1 mL, 1 mmol, 1 equiv.), *m*CPBA (0.69 g, 4 mmol, 4 equiv.). Isolated yield: 23%.

^1^H-NMR (400 MHZ, dichloromethane-*d*_*2*_, 25 °C, S17 in the Supporting Information, in agreement with literature sources[43]):

*δ* = 3.50 (*t*, Br-C***H***_***2***_-CH_2_CH(O)CH_2_, 2H), 3.06 (*m,* -CH(O)C***H***_***b***_H_d_, 1H), 2.80 (*t,* -C***H***(O)CH_2_, 1H), 2.56 (*m,* -CH(O)CH_b_***H***_***d***_, 1H), 2.13 (*m,* Br-CH_2_-C***H***_***e***_H_f_CH(O)CH_2_, 1H), 2.05 (*m,* Br-CH_2_-CH_e_***H***_***f***_CH(O)CH_2_, 1H).

#### Synthesis of 2-(3-bromopropyl)oxirane)

5-bromo-1-pentene (0.12 mL, 1 mmol), *m*CPBA (0.69 g, 4 mmol, 4 equiv.). Isolated yield: 21%.

^1^H-NMR (400 MHZ, chloroform-*d*, 25 °C, S18 in the Supporting Information, in agreement with literature sources[43]):

*δ* = 3.42 (*m*, Br-C***H***_***2***_-CH_2_CH_2_-, 2H), 2.89 (*m,* -CH(O)C***H***_***b***_H_d_, 1H), 2.72 (*t,* -C***H***(O)CH_2_, 1H), 2.47 (*m,* -CH(O)CH_b_***H***_***d***_, 1H), 1.99 (*m*, BrCH_2_-C***H***_***2***_-CH_2_-, 2H), 1.78 (*m,* Br-CH_2_CH_2_-C***H***_***f***_H_g_CH(O)CH_2_, 1H), 1.56 (*m,* Br-CH_2_CH_2_-CH_f_***H***_***g***_CH(O)CH_2_, 1H).

#### General synthesis of PFPAE-oxiranes

Silver oxide (2 equiv.) in 40:60 acetonitrile:PFB solution (25 mL) was added to a 125 mL 3-necked round-bottomed flask, and placed under N_2_ purge without expose the Ag_2_O to the light. PFPAE dicarboxylic acid (1 equiv., *M*_*n*_ = 3131 g mol^−1^) was slowly introduced at RT to the 3 necked round-bottomed under continuous stirring. The bromo-alkyl-oxirane (3 equiv.) was added dropwise at RT to the silver carboxylate mixture under continuous stirring. The reaction was complete after 48 h, as suggested by the formation of an AgBr whiteish precipitate and by GC–MS. Then, the reaction mixture was purified by flash chromatography; pure hexane was used first as eluent to remove fluorinated undesired byproduct as HFPO II hydrogen end cap (R_F_-H). Then, the polarity of the eluent was slowly increased up to 40:60 diethyl ether:hexane. Ceric ammonium molybdate was used as TLC stain. The conversion of the silver carboxylate salt into the corresponding epoxide was checked by the appearance, after a gently heating, of a dark blue spot on light blue background, located between the baseline of the TLC plate and the eluent front. After vacuum purging, a transparent oil was obtained as pure product (S19 in the Supporting Information).

#### Synthesis of perfluoropolyalkylether–methyl oxirane (PFPAE-MO)

PFPAE dicarboxylic acid (*M*_*n*_ = 3131 g mol^−1^, 1 mmol, 1.57 mL), silver oxide (2 equiv., 2 mmol, 0.462 g), epibromohydrin (3 equiv., 3 mmol, 0.26 mL). Isolated yield: 13%.

^1^H-NMR (400 MHz, MHz, benzene-*d*_*6*_ capillary, 25 °C, S20 in the Supporting Information):

δ = 4.77 (*m*, -OC***H***_***a***_H_b_-CH(O)CH_2_, 1H), 4.45 (*m*, -OCH_a_***H***_***b***_-CH(O)CH_2_, 1H), 3.36 (*s,* -C***H***(O)CH_2_, 1H), 2.99 (*s,* -CH(O)C***H***_***d***_H_e_, 1H), 2.80 (*s,* -CH(O)CH_a_***H***_***e***_, 1H). Impurities 6.28-6.15 ppm attributable to the HFPO II hydrogen end cap.

^13^C-NMR (101 MHz, benzene-*d*_*6*_ capillary, 25 °C, S21 in the Supporting Information):

δ = 158.78 (*d*, -***C***=O-), from 122.40 to 95.46 (*m*, −***C***F_3_ and ***C***F_2_ of repeat unit), 68.90 (*d*, -***C***H_2_CH(O)CH_2_), 47.53 (*s*, -CH_2_***C***H(O)CH_2_), 43.70 (*s*, -CH_2_CH(O)***C***H_2_).

^19^F-NMR (376.5 MHz, benzene-*d*_*6*_ capillary, 25 °C, S22 in the Supporting Information):

δ = − 146.68 (*s*, HFPO II hydrogen end cap), − 145.38 (*q*, C***F***(CF_3_) of repeat unit, mono and difunctional), − 132.16 (*ω* C***F***(CF_3_), mono and difunctional), − 130.50 (*s*, α C***F***_***2,***_ monofunctional), − 125.91 (*s*, -CF_2_C***F***_***2***_CF_2_O-_***,***_ difunctional), − 122.85 (*s*, -C***F***_***2***_CF_2_CF_2_O-_***,***_ difunctional), from − 87.17 to − 79.13 (C***F***_***3***_ and C***F***_***2***_ of repeat unit). The molecular weight of the product was calculated from the ^19^F-NMR spectra, as in S23 of the Supporting Information.

GC–MS (EI) fragmentation (S24 in the Supporting Information):

m/z = CHOCH_2_^+^ (43 m/z), CH_2_CHOCH_2_^+^ (57 m/z), CF_3_^+^ (69 m/z), OCH_2_CHOCH_2_^+^ (73 m/z), C_2_F_4_^+^ (100 m/z), C_2_F_5_^+^ (119 m/z), C_3_F_5_O^+^ (147 m/z), C_3_F_6_^+^ (150 m/z), C_3_F_7_^+^ (169 m/z), CF(CF_3_)C(O)OCH_2_CHOCH_2_^+^ (201 m/z).

#### Synthesis of perfluoropolyalkylether–ethyl oxirane (PFPAE-EO)

PFPAE dicarboxylic acid (*M*_*n*_ = 3131 g mol^−1^, 1 mmol, 1.57 mL), silver oxide (2 equiv., 2 mmol, 0.462 g), 2-(2-bromoethyl)oxirane (3 equiv., 3 mmol, 0.27 mL). Isolated yield: 10%.

^1^H-NMR (400 MHZ, benzene-*d*_*6*_ capillary, 25 °C, S25 in the Supporting Information):

δ = 4.75 (*m*, -OC***H***_***2***_-CH_2_-, 2H), 3.12 (*s,* -C***H***(O)CH_2_, 1H), 2.92 (*s,* -CH(O)C***H***_***c***_H_d_, 1H), 2.64 (*s,* -CH(O)CH_c_***H***_***d***_, 1H), 2.29 (*m, -*CH_2_-C***H***_***e***_H_f_-CH(O)CH_2_, 1H), 2.04 (*m,* -CH_2_- CH_e_***H***_***f***_-CH(O)CH_2_, 1H). Impurities 6.32-6.19 ppm attributable to the HFPO II hydrogen end cap.

^13^C-NMR (101 MHz, benzene-*d*_*6*_ capillary, 25 °C**,** S26 in the Supporting Information):

δ = 158.74 (*d*, -***C***=O-), from 122.45 to 95.51 (*m*, −***C***F_3_ and ***C***F_2_ of repeat unit), 65.53 (*s*, -O***C***H_2_CH_2_CH(O)CH_2_), 47.91 (*s*, -***C***H(O)CH_2_), 46.05 (*s*, -CH(O)***C***H_2_), 31.76 (*s*, -CH_2_***C***H_2_CH(O)CH_2_).

^19^F-NMR (376.5 MHz, benzene-*d*_*6*_ capillary, 25 °C, S27 in the Supporting Information):

δ = − 146.55 (*s*, HFPO II hydrogen end cap), − 145.25 (*q*, C***F***(CF_3_) of repeat unit, mono and difunctional), − 132.01 (*ω* C***F***(CF_3_), mono and difunctional), − 130.38 (*s*, α C***F***_***2***_, monofunctional), − 125.78 (*s*, -CF_2_C***F***_***2***_CF_2_O-_***,***_ difunctional), − 122.75 (*s*, -C***F***_***2***_CF_2_CF_2_O-_***,***_ difunctional), from − 87.01 to − 79.21 (CF_3_ and CF_2_ of repeat unit). The molecular weight of the product was calculated from the ^19^F-NMR spectra, as in S28 of the Supporting Information.

GC–MS (EI) fragmentation (S29 in the Supporting Information):

m/z = CHOCH_2_^+^ (43 m/z), CH_2_CHOCH_2_^+^ (57 m/z), CF_3_^+^ (69 m/z), CH_2_CH_2_CHOCH_2_^+^ (71 m/z), OCH_2_CH_2_CHOCH_2_^+^ (87 m/z), C_2_F_4_^+^ (100 m/z), C_2_F_5_^+^ (119 m/z), C_3_F_5_O^+^ (147 m/z), C_3_F_6_^+^ (150 m/z), C_3_F_7_^+^ (169 m/z), CF(CF_3_)C(O)OCH_2_CH_2_CHOCH_2_^+^ (215 m/z).

#### Synthesis of perfluoropolyalkylether–propyl oxirane (PFPAE-PO)

PFPAE dicarboxylic acid (*M*_*n*_ = 3131 g mol^−1^, 1 mmol, 1.57 mL), silver oxide (2 equiv., 2 mmol, 0.462 g), 2-(3-bromopropyl)oxirane (3 equiv., 3 mmol, 0.33 mL). Isolated yield: 13%.

^1^H-NMR (400 MHz, benzene-*d*_*6*_ capillary, 25 °C, S30 in the Supporting Information):

δ = 4.69 (*m*, -OC***H***_***2***_-CH_2_-, 2H), 3.06 (*q,* -C***H***(O)CH_2_, 1H), 2.89 (*t,* -CH(O)C***H***_***c***_H_d_, 1H), 2.61 (*q,* -CH(O)CH_c_***H***_***d***_, 1H), 2.16 (*m,* -OCH_2_C***H***_2_CH_2-_, 2H), 2.04 (*m,* -C***H***_***f***_H_g_-CH(O)CH_2_, 1H), 1.68 (*m,* -CH_f_***H***_***g***_-CH(O)CH_2_, 1H). Impurities 6.32-6.19 ppm attributable to the HFPO II hydrogen end cap. Other peaks attributable to a unknow byproduct.

^13^C-NMR (101 **MHz, benzene-*d***_***6***_ **capillary,** 25 ^o^C, S31 in the Supporting Information):

δ = 158.76 (*d*, -***C***=O-), from 122.51 to 95.52 (*m*, −***C***F_3_ and ***C***F_2_ of repeat unit), 68.14 (*s*, -O***C***H_2_CH_2_CH_2_-), 50.61 (*s*, -***C***H(O)CH_2_), 45.94 (*s*, -CH(O)***C***H_2_), 28.86 (*s*, -OCH_2_CH_2_***C***H_2_-), 25.35 (*s*, -OCH_2_***C***H_2_CH_2_-).

^19^F-NMR (376.5 MHz, benzene-*d*_*6*_ capillary, 25 °C, S32 in the Supporting Information):

δ = − 146.38 (*s*, HFPO II hydrogen end cap), − 145.21 (*q*, C***F***(CF_3_) of repeat unit, mono and difunctional), − 131.90 (*ω* C***F***(CF_3_), mono and difunctional), − 130.26 (*s*, α C***F***_***2,***_ monofunctional), − 125.70 (*s*, -CF_2_C***F***_***2***_CF_2_O-_***,***_ difunctional), − 122.69 (*s*, -C***F***_***2***_CF_2_CF_2_O-_***,***_ difunctional), from − 86.95 to − 79.14 (CF_3_ and CF_2_ of repeat unit). The molecular weight of the product was calculated from the ^19^F-NMR spectra, as in S33 of the Supporting Information.

GC–MS (EI) fragmentation (S34 in the Supporting Information):

m/z = CHOCH_2_^+^ (43 m/z), CH_2_CHOCH_2_^+^ (57 m/z), CF_3_^+^ (69 m/z), CH_2_CH_2_CHOCH_2_^+^ (71 m/z), CH_2_CH_2_CH_2_CHOCH_2_^+^ (85 m/z), C_2_F_4_^+^ (100 m/z), OCH_2_CH_2_CH_2_CHOCH_2_^+^ (101 m/z), C_2_F_5_^+^ (119 m/z), C_3_F_5_O^+^ (147 m/z), C_3_F_6_^+^ (150 m/z), C_3_F_7_^+^ (169 m/z), CF(CF_3_)C(O)OCH_2_CH_2_CH_2_CHOCH_2_^+^ (229 m/z).

### Synthesis of polymers by photoinduced polymerization

To PFPAE monomers was added the 2 wt% of photoinitiator, which is 50 wt% of triphenylsulfonium hexafluorophosphate salt in propylene carbonate, purchased from Sigma Aldrich (Italy).

The UV-sensitive mixtures were coated onto a glass substrate, using a wire-wound applicator. The glass substrate used was a microscope slide (pre-cleaned/ready-to-use) purchased from Thermo-Scientific, used as received without any surface treatment.

The samples were irradiated by means of a lamp Dymax ECE 5000, predominately producing UV-A light (440–360 nm) and some amount of UV-B light (320–280 nm) [44], using a light intensity of 150 mW cm^−2^ for 5 min. Samples with different thickness, going from 100 to 300 μm, were prepared. Light intensity was measured with a UV Power Puck II digital radiometer.

After irradiation, the samples were stored for at least 48 h at room temperature before evaluating their properties.

### Polymer characterization

The photopolymerization reaction was monitored by Fourier transform-infrared (FT-IR) analysis and by differential scanning calorimetry (DSC).

Real-time FT-IR spectroscopy analyses were performed using a Nicolet™ iS50 FT-IR spectrometer (Thermo Fisher Scientific). Simultaneously with the FT-IR scan acquisition (i.e., 1 scan/spectrum), thin films (i.e., about 10 μm on a Si wafer as substrate) of the reactive monomeric mixtures were irradiated for 10 min with a UV Hamamatsu LC8 lamp, provided of an optical fiber, having an intensity equal to 100 mW cm^−2^. Light intensity was measured with a UV Power Puck II digital radiometer.

Polymerization conversion was followed by monitoring the decrease in the absorbance of the vinyl ether groups at 1620 cm^−1^ or of the epoxy groups in the region 900–920 cm^−1^ as a function of irradiation time. The conversion (*χ*) is given by Eq. 1:

1 $$ \chi\ \left(\%\right)=\left(1-\frac{{\mathrm{A}}_t}{{\mathrm{A}}_0}\right)\times 100 $$

where A_*t*_ is the area of the peak of interest at the end of the irradiation, while *A*_0_ is the area of the peak of interest before the irradiation.

FT-IR spectra were also recorded in attenuated total reflectance mode (ATR), employing a Nicolet™ Smart iTX accessory equipped with an ATR diamond crystal, to check the final monomer-to-polymer conversion at the polymer surface after dark-curing (i.e., 48 h at room temperature).

Photo-DSC analyses were performed by means of a Mettler-Toledo DSC1 STARe System, appropriately modified with a Hamamatsu LC8 lamp with two beams, one for the sample side and the other for the reference side, as reported in previous works [45, 46]. To ensure equal illumination conditions throughout the sample volume, about 5 mg of each formulation were cured in open aluminum pans. The sample space was flushed with nitrogen. Two scans were performed on each sample to subtract the thermal effect of UV irradiation from the photocuring experiment, each one consisting of 4 min of temperature conditioning, 20 min of irradiation, and 4 min more without UV light. The measurements were carried out at 25 mW cm^−2^ of intensity and at room temperature, the increase of temperature due to the irradiation of the lamp being less than 1 °C at the end of the reaction. The reaction was considered completed when it was no longer possible to detect a change in the heat flux. The total heat of reaction was then calculated by the integration of the area under the exothermic peak [47]. The conversion (*χ*) over time was calculated by Eq. 2:

2 $$ \chi =\frac{\underset{0}{\overset{t}{\int }}\frac{\mathrm{d}h}{\mathrm{d}t}\ \mathrm{d}t}{\Delta {h}_{\mathrm{theor}}} $$

where d*h*/d*t* is the heat flow released by the sample during the photocuring and Δ*h*_theor_ is estimated considering the heat of reaction of 60 kJ mol^−1^ for the polymerization of the epoxy monomers [48], and the heat of reaction of 84 kJ mol^−1^ for the polymerization of the vinyl-ether monomers [49].

The insoluble fraction of the cross-linked samples (gel content) was evaluated using the weight loss of the network after a 24-h extraction by 1:1 DCM:PFB solution (expressed in weight fraction) at room temperature (ASTM D2765-16) [50]. The cross-linked fraction was then calculated by Eq. 3:

3 $$ \mathrm{Gel}\ \mathrm{content}\ \left(\%\right)=\left(1-\frac{{\mathrm{W}}_0-{\mathrm{W}}_t}{{\mathrm{W}}_0}\right) $$

where *W*_*t*_ is the weight of dry sample after extraction, while *W*_0_ is the weight of the original sample.

The thermal stability of the polymers was determined by thermogravimetric analysis (TGA) using a Mettler Toledo TGA/SDTA-851 instrument. Approximately 10 mg of the sample were placed in an alumina crucible and heated from room temperature to 800 °C under inert atmosphere (N_2_, 60 mL min^−1^), with a heating rate of 10 °C min^−1^.

Differential scanning calorimetry (DSC) thermograms were recorded using a Mettler-Toledo DSC1 STARe System in the temperatures ranging from − 100 to 50 °C using a heat/cool/heat method at a heating and cooling scanning rate of 10 °C min^−1^, under nitrogen flux. The glass transition temperature (*T*_g_) was determined using the midpoint of the heat capacity jump on the second heating cycle thermogram.

Static contact angle measurements were performed with a FTA 1000C instrument, equipped with a video camera and image analyzer, at room temperature with the sessile drop technique. The probe liquids were water and hexadecane, whose surface tension is 72.1 mN m^−1^ and 28.1 mN m^−1^, respectively. The surface energy was calculated by the Owens-Wendt’s geometric mean method [51]. Three to five measurements were performed on each sample, placing the liquid drops in different parts of the sample surface: the mean value and the error were determined.

Dynamic measurements were done by increasing the drop volume in the wetting process (advancing contact angle) and then decreasing it in the de-wetting phase (receding contact angle). The syringe needle remained in the drop during the whole process. In the first wetting step a 3 μL drop was created on the solid surface and then slowly increased in volume. In the second step, the surface was de-wetted and the drop size reduced, the whole cycle was repeated 3 times at 1 μL s^−1^, with a delay time of 1 s between each cycle. The contact angle hysteresis (CAH) was calculated as the difference between the measured advancing and receding contact angles.

For the sliding angle measurements [52], the same FTA 1000C apparatus was employed, equipped with a tilting stage. After placing a 20 μL liquid drop on the test surface, the film was tilted at 0.5° s^−1^. During the tilting stage, dynamic contact angles were measured, as well as the difference (∆α) between the advancing contact angle and the receding contact angle assessed on the sliding drop. The sliding angle was determined as the angle of inclination at which the drop starts to move and slides across the surface.

## Results and discussion

### Synthesis of the fluorinated monomers

**Scheme 2:**
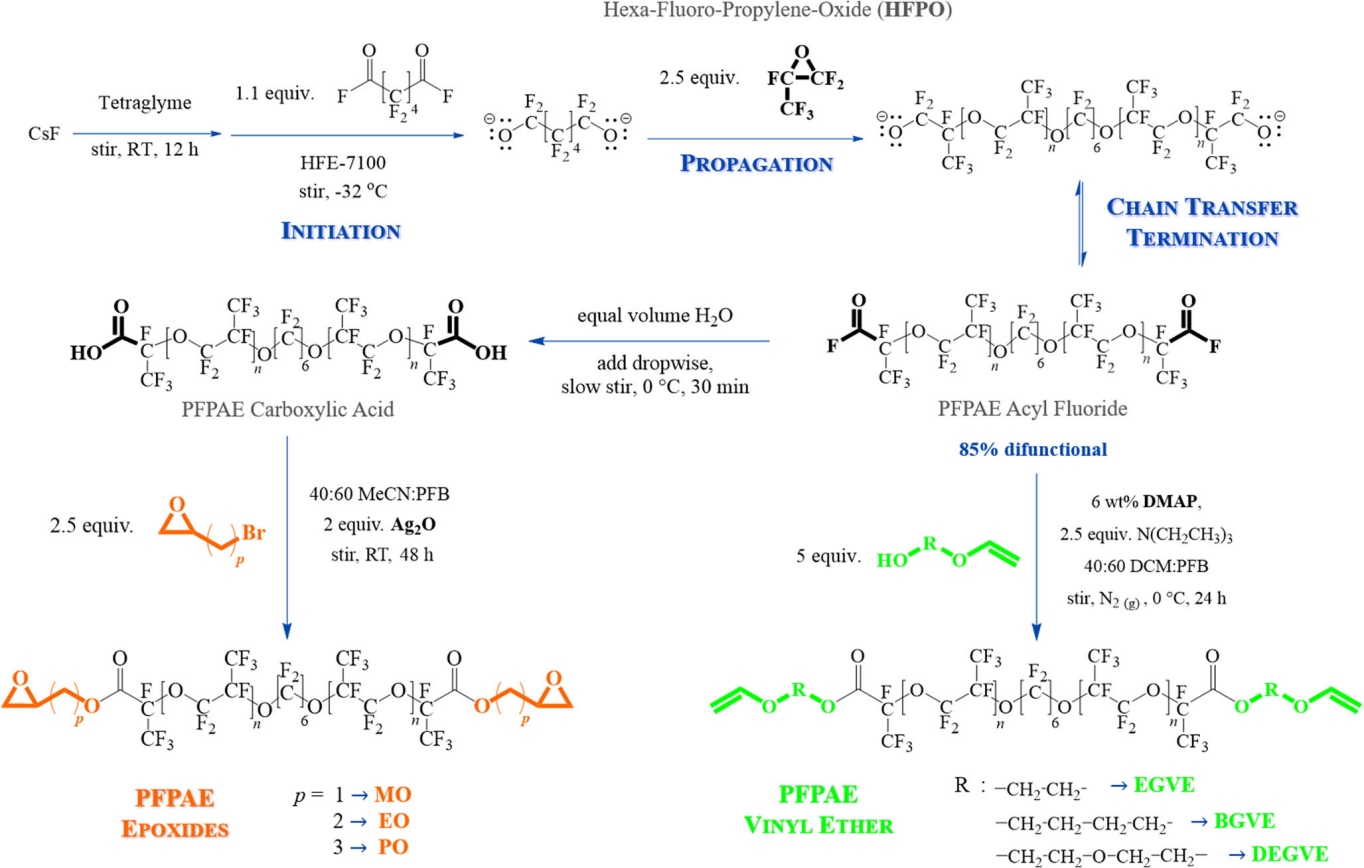
Synthetic paths for the functionalization of PFPAE monomers

The best synthetic pathways that allowed to obtain PFPAE-based vinyl ethers and epoxides are those described in Scheme 2.

**Scheme 3:**
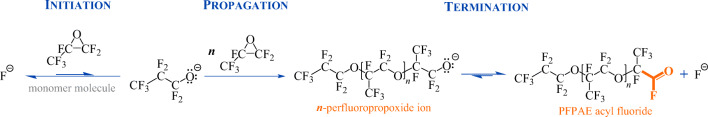
Reaction mechanism leading to a monofunctional product

First, the synthesis of telechelic PFPAE oligomers was conducted by anionic ring-opening polymerization of hexafluoropropylene oxide similarly reported by Ding et al. and Hill et al. [39, 53]. An alkoxide was prepared for allowing initiation: in our case, CsF in tetraglyme reacted with octafluoroadipoylfluoride; then, the monomer, HFPO, was added for propagation. By chain transfer, termination occurred and the telechelic PFPAE diacylfluoride was obtained. This product was used to prepare vinyl ethers, while it was oxidized to dicarboxylic acid for the subsequent preparation of epoxides [41, 42]. The initiation reaction by means of the octafluorodipoylfluoride is, however, accompanied by an initiation reaction due to HFPO itself, which is followed by a propagation leading to a monofunctional product (Scheme 3).

**Scheme 4:**
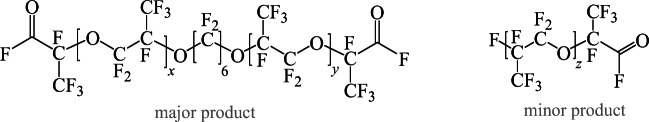
Structures of the monomers present in the final product mixture

Therefore, a monofunctional growing chain propagates producing monofunctional products and competes with the telechelic polymerization. Thus, the final product is a mixture of the structures depicted in Scheme 4.

The functionalization reactions to obtain vinyl ether-ended or oxirane-ended oligomers were conducted on the mixtures, as the separation of the monofunctional from the difunctional PFPAEs was unsuccessful, although numerous chromatography experiments were performed, trying to find the best eluents and TLC stains (S1 and S19 in the Supporting Information).

The synthesis of functional PFPAE derivatives, starting from the acyl fluoride or the carboxylic acid, was challenging as known from previous works [29, 54–57]. One of the main issues was related to the extreme non-polarity of the PFPAE chains [10, 20, 56]; indeed, the PFPAE solubility in solvents commonly used in organic chemistry is quite low. Moreover, even when the solubility of PFPAEs was optimized, the PFPAE starting oligomers reactivity was reduced due to the presence of the long-fluorinated chain. Therefore, the PFPAE vinyl ethers and epoxides were obtained with a low yield (~ 15% of isolated product), but with good purity (see ^1^H-NMR and ^13^C-NMR spectra in the Supporting Information). As well as the precursors, the obtained functional monomers (epoxides and vinyl ethers) contain both monofunctional and telechelic oligomers (Table 1). The telechelic oligomer molar percentage, as estimated by ^19^F-NMR spectra (see the Supporting Information for the calculation), is reported in Table 2. The molecular weight and the degree of polymerization of the products were also calculated by ^19^F-NMR spectra and are reported in Table 2, distinguishing the values of the monofunctional oligomer (*M*_*n*1_) and those of the difunctional oligomer (*M*_*n*2_). The functional group density *δ*_*FG*_ and the average functionality *f* of the PFPAE-based derivatives were evaluated, considering the experimental ratio between mono and difunctional oligomers: as reported in Table 2, the values are very close.

**Table 1:**
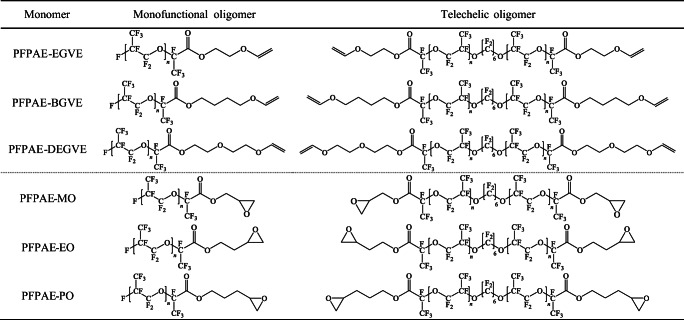
Chemical structure of the PFPAE oligomers

**Table 2:** Degrees of polymerization (${\overline{DP}}_{\mathrm{n}}$), molecular weights of monofunctional (***M***_*n*1_), and difunctional (***M***_*n*2_) oligomers, functional group density (***δ***_***FG***_), and average functionalities *f* of the products

Monomer	Telechelic monomer molar percentage (%)	${\overline{\mathrm{DP}}}_n$	M_*n*1_ (g mol^−1^)	M_*n*2_ (g mol^−1^)	*δ*_*FG*_ (mol g^–1^)	*f*
PFPAE-EGVE	55.5	6	1210	2715	0.078	1.5
PFPAE-BGVE^a^	48.9	8	1574	3445	0.065	1.5
PFPAE-DEGVE	42.8	7	1429	3150	0.067	1.4
PFPAE-MO	62.7	12	2206	4708	0.044	1.6
PFPAE-EO	42.6	8	1550	3394	0.062	1.4
PFPAE-PO	57.4	10	1193	4280	0.048	1.6

^a^Details on the preparation of this sample and its functionality are given in S35 of the Supporting Information

### Photoinduced polymerization

After the addition of a common cationic photoinitiator, the PFPAE derivatives were casted on glass slides and irradiated in air. The kinetics of the photopolymerization process was monitored in real time by FT-IR and photo-DSC.

**Fig. 1:**
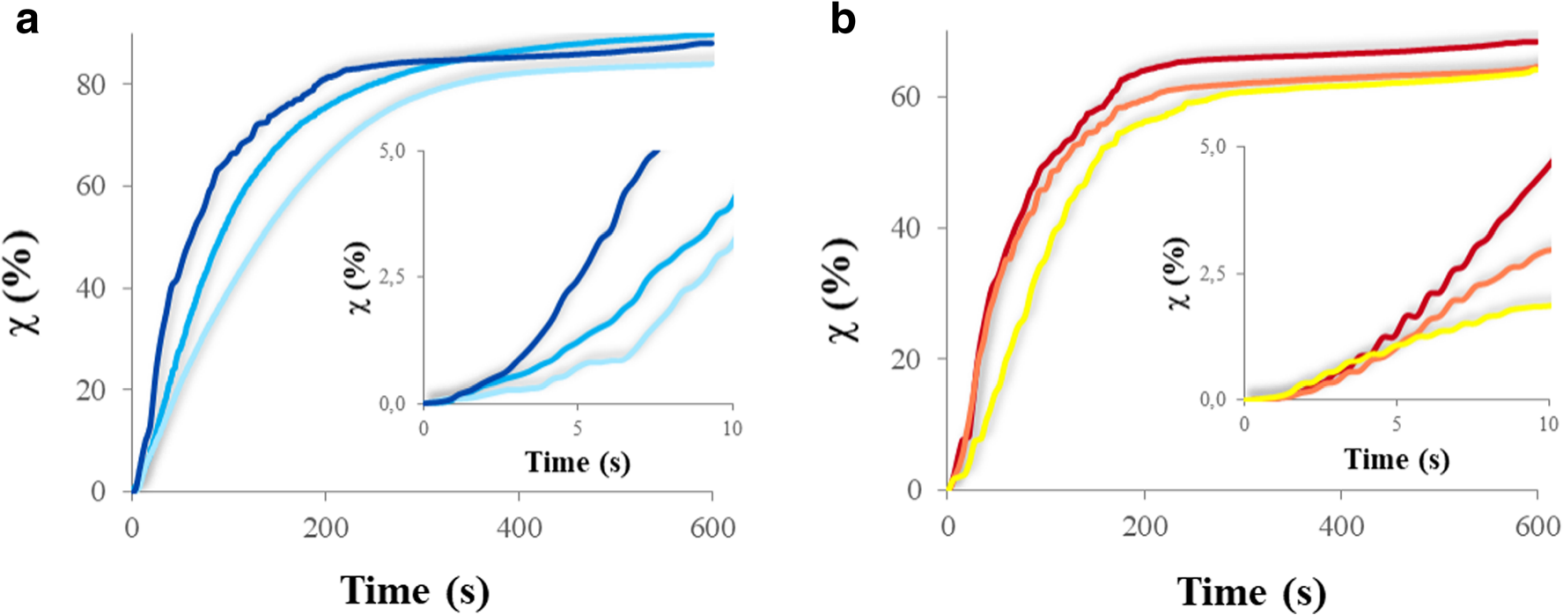
Real time FT-IR conversion (χ) versus time curves of the different fluoropolymers. The insets in **a**) and **b**) zoom the conversion curves at the beginning of the reaction, providing information on the initial polymerization rate. **a**)  PFPAE-EGVE,  PFPAE-BGVE,  PFPAE-DEGVE. **b**)  PFPAE-MO,  PFPAE-EO,  PFPAE-PO

In Fig. 1, the conversion curves by real-time FT-IR of the vinyl ether monomers (Fig. 1a) and of the epoxides (Fig. 1b) are reported. The conversion was evaluated during the irradiation of the sample with UV light by monitoring the decrease of the intensity of the band of the double bond (1620 cm^−1^) and of the band of the oxirane ring (900–920 cm^−1^), for the polymers with vinyl ether and epoxide reactive groups, respectively. The curves follow typical cationic photo-induced polymerization trends exhibited by vinyl ethers and epoxides [58–61] with the kinetics regimes and the complex mechanisms occurring during photopolymerization reactions discussed in literature [60, 62–66].

The fluorinated vinyl ethers (inset of Fig. 1a) react faster than the epoxides polymers (inset of Fig. 1b), as it can be noticed by the higher conversion rate in the first 10 s of irradiation; in about 180 s, their conversion is nearly 60–70% (Fig. 1a), while for PFPAE epoxides is lower than 50% in the same time scale (Fig. 1b). At the end of the irradiation (i.e., 600 s), the final conversion of the fluorinated vinyl ethers is higher than 80% (Fig. 1a), while the fluoroepoxides reach a maximum conversion of approximately 65% (Fig. 1b).

Comparing the oligomers having the same photoreactive groups, it is shown in the insets of Fig. 1 that the polymerization rate follows the order PFPAE-EGVE < PFPAE-BGVE < PFPAE-DEGVE (Fig. 1a) and PFPAE-MO < PFPAE-EO < PFPAE-PO (Fig. 1b). Interestingly, there is a different reactivity, although the functionality and the functional density of the systems are very similar (see Table 2). Therefore, one could suppose that the reactivity depends on the structure of the oligomers, in particular on the length of the hydrogenated spacer, which corresponds to the number of methylene units between the fluorinated chains and the reactive end-groups. The PFPAEs functionalized with longer hydrogenated spacer show a faster initial polymerization rate, also reaching a higher maximum degree of conversion. The effect of the spacer on reactivity has been observed and studied, for both living cationic [37, 64, 67] and radical [68, 69] polymerizations. A long-fluorinated chain induces an electron withdrawing effect, which decreases the monomer reactivity [70–72]. Increasing the number of methylene units between the fluorinated segment and the vinyl ether group or epoxy group, one can modulate the polymerization rate.

**Fig. 2.**
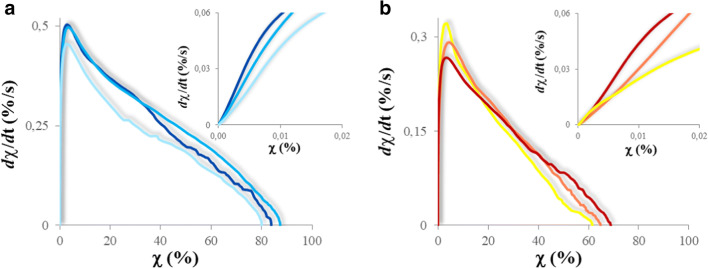
First derivative of the photopolymerization conversion (dχ/dt) versus conversion (χ) for the different polymers, by photo-DSC. The insets in **a** and **b** zoom the dχ/dt versus χ curves at the beginning of the reaction, providing information on the initial polymerization rate. **a**)  PFPAE-EGVE,  PFPAE-BGVE,  PFPAE-DEGVE. **b**)  PFPAE-MO,  PFPAE-EO,  PFPAE-PO

The polymerization kinetics of the PFPAE polymers was also studied employing an alternative, but accurate and reproducible [26, 47] method: the photo-DSC. In Fig. 2a, b, the first derivative of the conversion curve, which represents the reaction rate, is plotted as a function of the conversion (photo-DSC conversion curves are reported in S36 of the Supporting Information).

Also in this case, considering both the initial and the maximum conversion rate, the PFPAE vinyl ethers (inset of Fig. 2a, S37 in the Supporting Information) are demonstrated to react faster than the fluorinated epoxides (inset of Fig. 2b, S37 in the Supporting Information). Furthermore, the conversion reached by the vinyl ethers (around 80%, Fig. 2a) is higher than the final degree of conversion showed by the epoxides (ca. 60%, Fig. 2b).

For both the fluorinated vinyl ethers and the epoxides, the photo-DSC analyses confirmed that the different number of methylene units between the fluorinated segment and the reactive end-group has a non-negligible influence on the initial polymerization rate. In general, functionalized PFPAEs having shorter hydrogenated spacers show slower polymerization rates and lower conversions (Fig. 2a, b), as already observed in the real-time FT-IR kinetics plots (Fig. 1a, b).

Furthermore, considering the gel point, taken as the maximum of the conversion rate, it can be observed that the transition from a polymerizing system to a network slowing the reaction and changing its kinetic regime occurs for lower degrees of conversion for the PFPAEs functionalized with shorter hydrogenated spacers (Fig. 2a, b).

The conversion degrees obtained by different techniques are collected in Table 3. The comparison of the data from real time FT-IR and photo-DSC analysis, evaluated at the same UV dose (i.e., 25 J cm^−2^), are in good agreement. Then, the samples were stored for 48 h to allow the “dark curing” reaction to occur [60, 73, 74], i.e., further monomers conversion after the end of the irradiation, and the photopolymerization conversions were estimated by FT-IR ATR (Table 3, S38 in the Supporting Information). Data show that the cationic photopolymerization spontaneously continues in the dark leading to higher conversion values, i.e., almost complete conversion for the fluoro-vinyl ethers, and higher than 70% for the PFPAE-epoxides.

**Table 3:** Photopolymerization conversions and gel content of the UV-cured homopolymers

Homopolymer	Degree of conversion (%)			Insoluble Fraction (%)
	Photo-DSC^a^	FT-IR^a^	FT-IR ATR^b^	Gel content^c^
PFPAE-EGVE	80	78	97	93
PFPAE-BGVE	87	84	94	90
PFPAE-DEGVE	84	83	95	96
PFPAE-MO	61	60	73	84
PFPAE-EO	65	62	71	87
PFPAE-PO	69	66	76	90

^a^Value of the plateau in the conversion curves for a UV dose of 25 J cm^−2^

^b^Value determined 48 h after the end of irradiation (5 min at 150 mW cm^−2^)

^c^Value determined on samples stored 48 h after the end of irradiation (5 min at 150 mW cm^−2^), after 24 h extraction by 50/50 DCM/PFB at RT

The residual insoluble fraction (gel content, Table 3) of the photopolymerized networks, determined by solvent extraction, is in good agreement with the trend of the conversion data collected by FT-IR ATR. The values are rather high (i.e., always > 80%), showing polymers are cross-linked networks, and higher values are found for the polymers obtained from the PFPAE derivatives with longer hydrogenated spacers. However, the photocured films (colored but transparent) were soft, and more similar to highly viscous liquids rather than solid films due to the high content of PFPAE chains, which are very mobile [32, 75, 76] with a low *T*_g_, at the same time the hydrogenated phase is rubbery at room temperature (as it will be discuss later).

### Photopolymers characterization

**Fig. 3:**
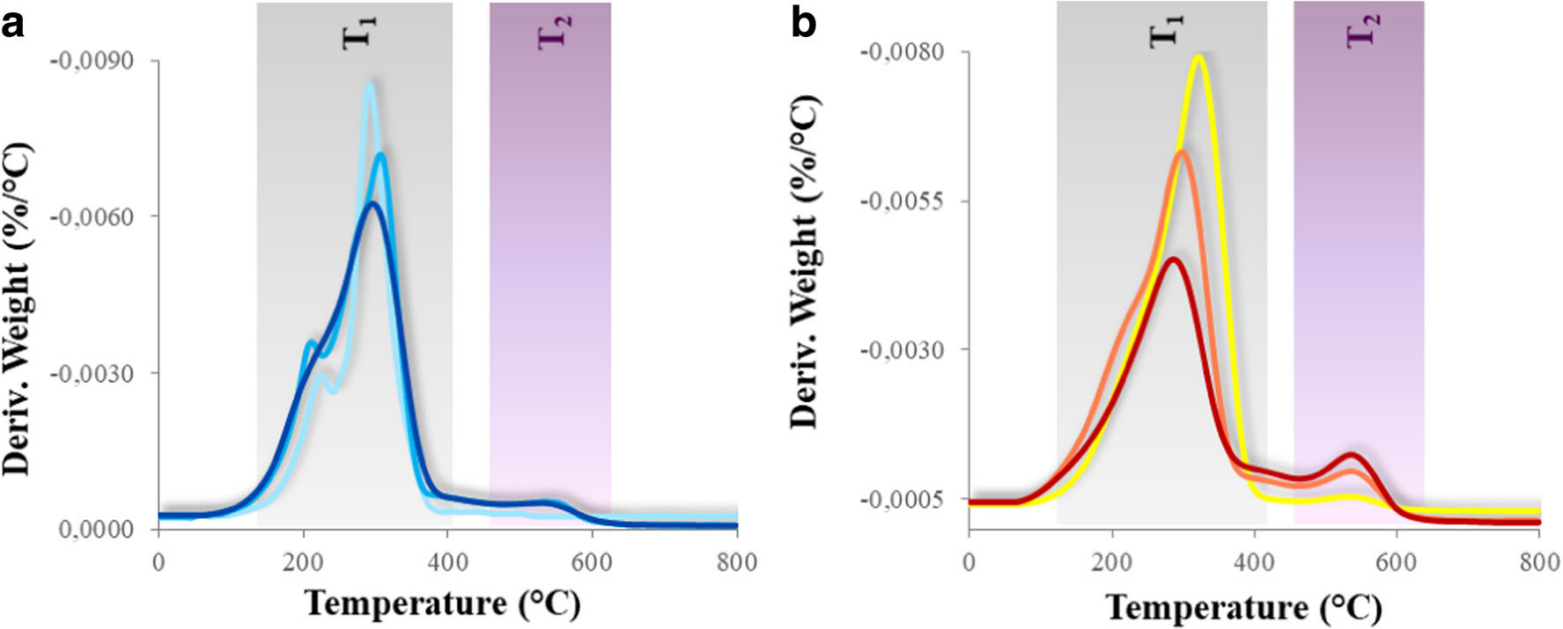
Derivative weight loss (DTG) curves, of UV-cured polymers. **a**)  PFPAE-EGVE,  PFPAE-BGVE,  PFPAE-DEGVE. **b**)  PFPAE-MO,  PFPAE-EO,  PFPAE-PO

The thermal resistance of the photo-cured polymers was assessed by TGA: the derivative weight loss (DTG) curves, recorded in inert atmosphere, are shown in Fig. 3. The DTG curves of the PFPAE polymers display two distinct regions of weight loss, implying that two main steps of degradation occur in those samples.

The first step, between 150 and 400 °C, is responsible for the scission of the perfluoropolyalkylether main chains [69] for both the PFPAE vinyl ethers and epoxides (*T*_1_ region, Fig. 3a, b). The PFPAE thermal degradation may be triggered by the presence of Lewis acids such as AlF_3_ [77] or Al_2_O_3_ [78] and can result in a complete loss of the fluorinated segments above 180 °C. Moreover, the thermal behavior could be adversely affected by residues of the superacids generated during the photoinitiation of the cationic polymerization and still present in the network.

The maximum weight loss of the first degradation step (ca. 30% by weight for each polymer) is higher for the functional PFPAE derivatives with a shorter hydrogenated spacer.

During the second weight loss, which occurs between 450 and 600 °C (*T*_2_ region, Fig. 3a, b), the C–C and C–O bonds of the vinyl ethers and epoxides end-groups are further destroyed due to the high temperatures [69]. The maximum temperature of this second weight loss stage corresponds to ca. the 90 wt.% of degradation (~ 530 °C), and it is almost the same for all the polymers. The final residue is zero, indicating that in N_2_ a complete degradation of material occurs above 600 °C.

As suggested by the crosscheck of the degradation temperature data (collected in S39 of the Supporting Information) with the photopolymerization kinetics data (Table 2), it seems that, in general, as expected, with a lowering of the conversion, the photocured polymers exhibit a poorer thermal resistance.

**Fig. 4:**
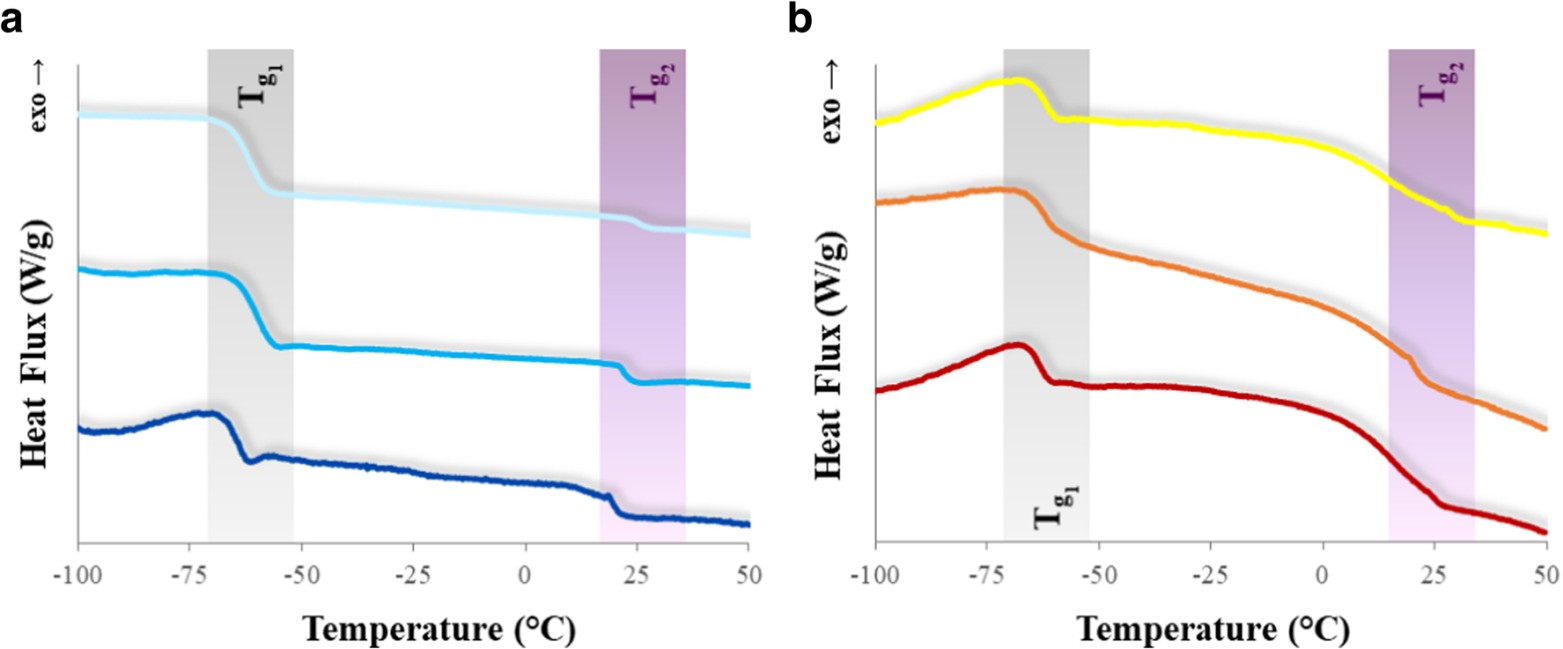
DSC thermograms of the photocured homopolymer. **a**)  PFPAE-EGVE,  PFPAE-BGVE,  PFPAE-DEGVE. **b**)  PFPAE-MO,  PFPAE-EO,  PFPAE-PO

The phase transitions of the fluorinated homopolymers can be observed in the DSC curves reported in Fig. 4. The photopolymers show two different glass transitions: the phase transition occurring at lower temperature (*T*_g1_ ≅− 65 °C) can be attributed to the PFPAE moieties, while the glass transition that takes place at higher temperature (*T*_g2_ = 17–23 °C) can be assigned to the alkyl spacing groups of the functionalized fluoromonomers or to a interphase region, mainly containing hydrogenated segments [79, 80]. These results indicate biphasicity of the PFPAE polymers at molecular level, since their structure contains a fluorinated chain linked to alkyl spacers bearing hydrogenated end-groups [81]. However, films are transparent at a visual inspection. As reported before, the photocured films are rubbery at room temperature.

Wettability of the UV-cured PFPAE polymers was evaluated by contact angle measurement (Table 4) using water (polar solvent) and hexadecane (apolar solvent). The water static contact angle of the photopolymerized PFPAEs was higher than 85°, while a contact angle of about 70° was measured when hexadecane was used as measuring liquid. These contact angle values are commonly exhibited by highly fluorinated materials [31, 32, 38]. They are similar to those shown by photocrosslinked copolymers containing less than 5 wt% of a fluorinated monomer with the same PFPAE chain structure [31, 32]. Neither the length of the hydrogenated spacer nor the crosslinking degree seem to affect the wettability of the fluorinated homopolymers.

**Table 4:** Static contact angle (*θ*), surface energy (*γ*), divided in its dispersive (*γ*^*d*^) and polar (*γ*^*p*^) components, and contact angle hysteresis (CAH), estimated from the difference between the advancing (θ_adv_) and the receding (θ_rec_) angles, of the UV-cured fluoropolymers

Homopolymer	Static contact angle (°)		Surface energy (mN m^−1^)			Dynamic contact angle (°)		Contact angle hysteresis (°)
	θ_water_	θ_hexadecane_	*γ*^*d*^	*γ*^*p*^	*γ*	θ_adv_ (°)	θ_rec_ (°)	CAH
PFPAE-EGVE	90	63	15	6	**21**	90	72	**18**
PFPAE-BGVE	99	78	10	5	**15**	93	77	**16**
PFPAE-DEGVE	93	71	12	7	**19**	89	76	**13**
PFPAE-MO	89	70	12	8	**20**	87	76	**11**
PFPAE-EO	86	67	13	9	**22**	89	75	**14**
PFPAE-PO	88	68	13	8	**21**	89	77	**12**

As expected on the basis of the wettabilities, the surface energy (γ) of the PFPAE polymers is very low (Table 4), and especially the polar component, γ^p^, that is lower than 10 mN m^−1^, is the most responsible of the drop of γ. Low surface energy can guarantee a difficult wettability of most surfaces and repellency toward most liquids: anti-graffiti and anti-staining properties, for example, can be achieved [31].

Further information on the surface properties of the UV-cured polymers can be obtained by the contact angle hysteresis (CAH), i.e., the difference between advancing and receding contact angle (S40 in the Supporting Information). Hysteresis is mainly caused by surface roughness and/or heterogeneity and commonly occurs in materials containing at the surface both hydrophobic and hydrophilic groups [27, 82].

PFPAE polymers show hysteresis values that are generally very low (Table 4), independent from the monomer structure (namely the hydrogenated spacer between the fluorinated chain and the reactive end-group), and, therefore, related to the main PFPAE chain which is the same in all monomers. These low hysteresis values suggest that the photocured PFPAE polymers can be exploited as anti-staining and self-cleaning materials [31, 83].

**Fig. 5:**
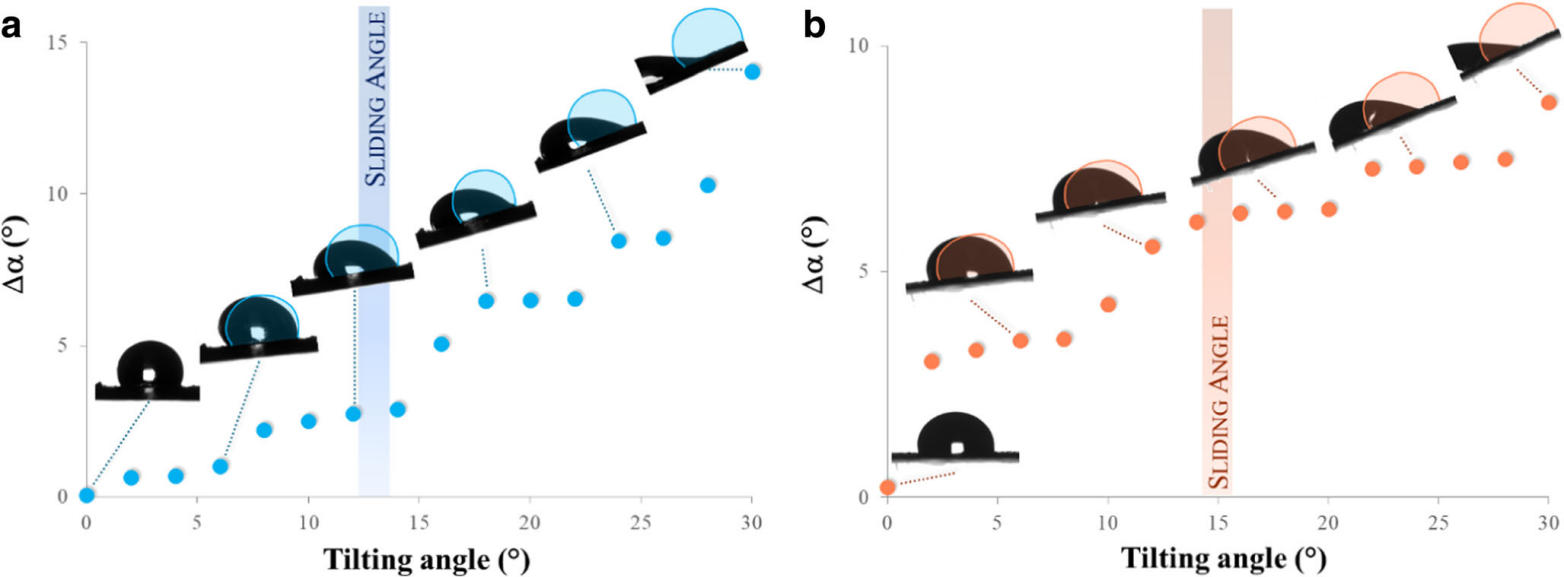
∆α and threshold sliding angle for a drop of water on PFPAE-BGVE (**a**) and PFPAE-EO (**b**) UV-cured films. The blue and orange shadows represent the original shape of the water drops at a tilting angle of 0°

For this reason, the PFPAE polymers surface behavior was also tested by sliding angle measurements. The polymer film surface, on which a water drop is deposited, is tilted, and a threshold sliding angle can be defined as the tilting angle at incipient droplet motion [84]. Figure 5 shows a graph of ∆α (i.e., the difference between the advancing contact angle and the receding contact angle measured on the sliding drop during the tilting stage) versus the tilting angle on a UV-cured film of PFPAE-BGVE (Fig. 5a) and PFPAE-EO (Fig. 5b). As it can be observed, the threshold sliding angle is comprised between 12° and 15°. Similar results were found for most of the films prepared: these values are comparable to those previously reported in similar surfaces containing perfluoropolyalkylether chains [31], demonstrating that these polymers can be exploited as self-cleaning materials.

## Conclusions

The synthesis of polyfunctional vinyl ether and epoxide monomers containing the same PFPAE chain and different hydrogenated spacers between the perfluoropolyether chain and the photoreactive groups was successfully performed, although the reactions had low yields.

The PFPAE derivatives with an average functionality around 1.5 were successfully employed in the photo-induced cationic polymerization obtaining transparent films, as assessed by visual inspection. The interesting photopolymerization behavior, of both the PFPAE vinyl ethers and epoxides, was studied using different techniques, namely real time FT-IR and photo-DSC: cross-linked polymers with high gel content were obtained, while conversion was in the range 60–90% depending on the monomer structure and functionality. In particular, the reactivity of the oligomers depended on the spacer, i.e., the length of the hydrogenated chain connecting the PFPAE and the functional group.

The obtained polymers exhibited biphasicity as two distinct *T*_g_s were present, one at low temperature due to the PFPAE blocks, the second slightly below room temperature (25 °C) due to the hydrogenated domains. Therefore, the materials are soft. TGA results indicated that the thermal stability of the PFPAE polymers can be adversely affected by the presence of acids in the cured networks. Interestingly, hydrophobic and oleophobic properties were shown, and very low contact angle hysteresis and sliding angle. Based on these results, we can conclude that these materials can be proposed to produce high-performance and self-cleaning coatings.

## Supplementary information

The Supporting Information is available free of charge. Characterization of PFPAE vinyl ethers and epoxides monomer; calculation of the molecular weight of the PFPAE monomers; synthesis and characterization of the monofunctional PFPAE-BGVE; study of the polymerization kinetics by photo-DSC; ATR FT-IR study of the photopolymerization reaction; TGA study of the thermal stability, Contact Angle Hysteresis study.

The following files are available free of charge.

ESM 1 (DOCX 2980 KB)

## References

[1] Sangermano M, Razza N, Crivello JV (2014). Cationic UV-curing: technology and applications. Macromol Mater Eng.

[2] Yagci Y, Jockusch S, Turro NJ (2010). Photoinitiated polymerization: advances, challenges, and opportunities. Macromolecules.

[3] Peterson BM, Lin S, Fors BP (2018). Electrochemically controlled cationic polymerization of vinyl ethers. J Am Chem Soc.

[4] Michaudel Q, Chauviré T, Kottisch V, Supej MJ, Stawiasz KJ, Shen L, Zipfel WR, Abruña HD, Freed JH, Fors BP (2017). Mechanistic insight into the photocontrolled cationic polymerization of vinyl ethers. J Am Chem Soc.

[5] Jiang K, Han S, Ma M, Zhang L, Zhao Y, Chen M (2020). Photoorganocatalyzed reversible-deactivation alternating copolymerization of chlorotrifluoroethylene and vinyl ethers under ambient conditions: facile access to main-chain fluorinated copolymers. J Am Chem Soc.

[6] Friesen CM, Améduri B (2018). Outstanding telechelic perfluoropolyalkylethers and applications therefrom. Prog Polym Sci.

[7] Bongiovanni R, Medici A, Zompatori A, Garavaglia S, Tonelli C (2012). Perfluoropolyether polymers by UV curing: Design, synthesis and characterization. Polym Int.

[8] Marchionni G, Ajroldi G, Cinquina P, Tampellini E, Pezzin G (1990). Physical properties of perfluoropolyethers: dependence on composition and molecular weight. Polym Eng Sci.

[9] Ameduri B (2018). Fluoropolymers: the right material for the right applications. Chem - A Eur J.

[10] Wang Y, Betts DE, Finlay JA, Brewer L, Callow ME, Callow JA, Wendt DE, DeSimone JM (2011). Photocurable amphiphilic perfluoropolyether/poly(ethylene glycol) networks for fouling-release coatings. Macromolecules.

[11] Hu Z, Finlay JA, Chen L, Betts DE, Hillmyer MA, Callow ME, Callow JA, DeSimone JM (2009). Photochemically cross-linked perfluoropolyether-based elastomers: Synthesis, physical characterization, and biofouling evaluation. Macromolecules.

[12] Cui Z, Drioli E, Lee YM (2014). Recent progress in fluoropolymers for membranes. Prog Polym Sci.

[13] Sianesi D, Zamboni V, Fontanelli R, Binaghi M (1971). Perfluoropolyethers: their physical properties and behaviour at high and low temperatures. Wear.

[14] Malinverno G, Pantini G, Bootman J (1996). Safety evaluation of perfluoropolyethers, liquid polymers used in barrier creams and other skin-care products. Food Chem Toxicol.

[15] Young CJ, Hurley MD, Wallington TJ, Mabury SA (2006). Atmospheric lifetime and global warming potential of a perfluoropolyether. Environ Sci Technol.

[16] Yang C, Tang D (2000). Patient-specific carotid plaque progression simulation. C Model Eng Sci.

[17] Wang Z, Dewitt JC, Higgins CP, Cousins IT (2017). A never-ending story of Per- and polyfluoroalkyl substances (PFASs)?. Environ Sci Technol.

[18] European Commission (2016) Commission regulation (EU) No 10/2011 on plastic materials and articles intended to come into contact with food. Brussels

[19] U.S. FDA (2019) Components of paper and paperboard in contact with aqueous and fatty foods. In: Indirect FOOD Addit. Pap. Pap. COMPONENTS - Subst. Use Only as Components Pap. Pap. https://www.accessdata.fda.gov/scripts/cdrh/Cfdocs/cfCFR/CFRSearch.cfm?fr=176.170

[20] U.S. FDA (2019) Chromium (Cr III) complex of N-ethyl-N-heptadecylfluoro-octane sulfonyl glycine. In: Indirect FOOD Addit. Pap. Pap. COMPONENTS - Subst. Use Only as Components Pap. Pap. https://www.accessdata.fda.gov/scripts/cdrh/Cfdocs/cfCFR/CFRSearch.cfm?fr=176.160

[21] OECD/UNEP Global PFC Group (2013) United Nations Environment Programme: Synthesis paper on per- and polyfluorinated chemicals (PFCs), Environment, Health and Safety, Environment Directorate, OECD

[22] OECD (2018) Toward a new comprehensive global database of per- and polyfluoroalkyl substances (PFASs): Summary Report on Updating the OECD 2007 List of Per- and Polyfluoroalkyl Substances (PFASs). Ser Risk Manag No. 39.:1–24

[23] Vitale A, Priola A, Tonelli C, Bongiovanni R (2013). Nanoheterogeneous networks by photopolymerization of perfluoropolyethers and acrylic co-monomers. Polym Int.

[24] Vitale A, Priola A, Tonelli C, Bongiovanni R (2014). Improvement of adhesion between a UV curable fluorinated resin and fluorinated elastomers: EFFECT of chemical modification onto the mechanical properties of the joints. Int J Adhes Adhes.

[25] Vitale A, Quaglio M, Marasso SL, Chiodoni A, Cocuzza M, Bongiovanni R (2013). Direct photolithography of perfluoropolyethers for solvent-resistant microfluidics. Langmuir.

[26] Vitale A, Quaglio M, Cocuzza M, Pirri CF, Bongiovanni R (2012). Photopolymerization of a perfluoropolyether oligomer and photolithographic processes for the fabrication of microfluidic devices. Eur Polym J.

[27] Bongiovanni R, Malucelli G, Pollicino A, Tonelli C, Simeone G, Priola A (1998). Perfluoropolyether structures as surface modifying agents of UV-curable systems. Macromol Chem Phys.

[28] Bongiovanni R, Di Meo A, Pollicino A (2008). New perfluoropolyether urethane methacrylates as surface modifiers: effect of molecular weight and end group structure. React Funct Polym.

[29] Bonneaud C, Decostanzi M, Burgess J, Trusiano G, Burgess T, Bongiovanni R, Joly-Duhamel C, Friesen CM (2018). Synthesis of α,β-unsaturated esters of perfluoropolyalkylethers (PFPAEs) based on hexafluoropropylene oxide units for photopolymerization. RSC Adv.

[30] Bonneaud C, Burgess J, Vitale A, Trusiano G, Joly-Duhamel C, Friesen CM, Bongiovanni R (2020). Perfluoropolyalkylether maleimides for protection from oxygen inhibition and surface modification of photoinitiator-free UV-cured polymers. Front Mater.

[31] Trusiano G, Rizzello M, Vitale A, Burgess J, Friesen CM, Joly-Duhamel C, Bongiovanni R (2019). Modification of photocurable epoxides by new perfluoropolyalkylether alcohols for obtaining self-cleaning coatings. Prog Org Coat.

[32] Trusiano G, Vitale A, Rizzello M, Bonneaud C, Joly-Duhamel C, Friesen CM, Bongiovanni R (2019). Controlling perfluoropolyalkylether rearrangements at the surface of photocured networks. Eur Polym J.

[33] Trusiano G, Vitale A, Bonneaud C, et al (2020) Vinyl ethers and epoxides photoinduced copolymerization with perfluoropolyalkylether monomers. Colloid Polym Sci 1–13. 10.1007/s00396-020-04723-3 10.1007/s00396-020-04723-3 PMC7952294 33785978

[34] Sangermano M, Bongiovanni R, Malucelli G, Priola A, Thomas RR, Medsker RE, Kim Y, Kausch CM (2004). Synthesis and cationic photopolymerization of a new fluorinated oxetane monomer. Polymer (Guildf).

[35] Sangermano M, Messori M, Rizzoli A, Grassini S (2010). UV-cured epoxy coatings modified with perfluoropolyether-based materials. Prog Org Coat.

[36] Vitale A, Sangermano M, Bongiovanni R, Burtscher P, Moszner N (2014). Visible light curable restorative composites for dental applications based on epoxy monomer. Materials (Basel).

[37] Vitale A, Cominotti M, Ameduri B, Bongiovanni R (2016). Semi-interpenetrating polymer networks by cationic photopolymerization: Fluorinated vinyl ether chains in a hydrogenated vinyl ether network. Eur Polym J.

[38] Sangermano M, Bongiovanni R, Priola A, Pospiech D (2005). Fluorinated alcohols as surface-active agents in cationic photopolymerization of epoxy monomers. J Polym Sci Part A Polym Chem.

[39] Hill JT, Erdman JP (1977) Anionic polymerization of fluorocarbon epoxides. In: Saegusa T, Goethals E (eds) Ring-Opening Polymerization. ACS Symposium Series, Washington, pp 269–284

[40] Hill JT (1974). Polymers From Hexafluoropropylene Oxide (HFPO). J Macromol Sci Part A - Chem.

[41] Eleuterio HS (1972). Polymerization of Perfluoro Epoxides. J Macromol Sci Part A - Chem.

[42] Hill JT (1977). Octafluoroisobutylene epoxide derivatives. J Fluor Chem.

[43] Cruickshank PA, Fishman M (1969). Studies in alkylation. II. Reactions of Epoxy alkyl Bromides. J Organomet Chem.

[44] Dymax (2020) ECE 5000: Versatile UV flood lamp curing for a variety of industrial applications. In: Dymax ECE Ser. UV Light. Flood. https://dymax.com/products/equipment/light-curing-equipment/flood-curing-systems/ece-5000

[45] Fernández-Francos X, Salla JM, Cadenato A, Morancho JM, Mantecón A, Serra A, Ramis X (2007). Novel thermosets obtained by UV-induced cationic copolymerization of DGEBA with an spirobislactone. J Polym Sci Part A Polym Chem.

[46] Morancho JM, Cadenato A, Ramis X, Fernández-Francos X, Salla JM (2010). Thermal curing and photocuring of an epoxy resin modified with a hyperbranched polymer. Thermochim Acta.

[47] Dalle Vacche S, Geiser V, Leterrier Y, Månson JAE (2010). Time-intensity superposition for photoinitiated polymerization of fluorinated and hyperbranched acrylate nanocomposites. Polymer (Guildf).

[48] Lohse F, Zweifel H (1986) Photocrosslinking of Epoxy Resins. In: Dušek K (ed) Advances in Polymer Science. Springer, Berlin Heidelberg, pp 61–81

[49] Sawada H (1969). Chapter 2. Heat of Polymerization. J Macromol Sci Part C.

[50] ASTM (2011) D2765-11: Standard test methods for determination of gel content and swell ratio of crosslinked ethylene plastics. 1–8

[51] Wu S (2017) Polymer interface and adhesion. Polym Interface Adhes 1–630. 10.1201/9780203742860

[52] Pierce E, Carmona FJ, Amirfazli A (2008). Understanding of sliding and contact angle results in tilted plate experiments. Colloids Surf A Physicochem Eng Asp.

[53] Ding J-F, Proudmore M, Mobbs R (1992). Controlled synthesis of α,β-difunctional poly(hexafluoropropylene oxide). Die Makromol Chem.

[54] Yao W, Li Y, Huang X (2014). Fluorinated poly(meth)acrylate: synthesis and properties. Polymer (Guildf).

[55] Turri S, Scicchitano M, Tonelli C (1996). End group chemistry of fluoro-oligomers: Highly selective syntheses of diepoxy, diallyl, and tetraol derivatives. J Polym Sci Part A Polym Chem.

[56] Surya Prakash GK, Hu J, Olah GA (2003). Alkylation of in situ generated fluorinated alkoxides: Novel synthesis ofpartially fluorinated ethers. Arkivoc.

[57] Bongiovanni R, Malucelli G, Messori M, Pilati F, Priola A, Tonelli C, Toselli M (2000). Acrylic polyester resins containing perfluoropolyethers structures: Synthesis, characterization, and photopolymerization. J Appl Polym Sci.

[58] Fouassier JP, Lalevée J (2012) Cationic photoinitiating systems. Photoinitiators for Polymer Synthesis. Wiley-VCH, Weinheim, pp 289–341

[59] Crivello JV, Lam JHW (1979). Photoinitiated cationic polymerization with triarylsulfonium salts. J Polym Sci Polym Chem Ed.

[60] Yagci Y, Schnabel W (1988). Light-induced cationic polymerization. Makromol Chem Macromol Symp.

[61] Decker C (2002). Light-induced crosslinking polymerization. Polym Int.

[62] Bowman CN, Kloxin CJ (2008). Toward an enhanced understanding and implementation of photopolymerization reactions. AICHE J.

[63] Goodner MD, Bowman CN (1999). Modeling primary radical termination and its effects on autoacceleration in photopolymerization kinetics. Macromolecules.

[64] Choi W, Sawamoto M, Higashimura T (1988). Living cationic homo- and copolymerizations of vinyl ethers bearing a perfluoroalkyl pendant. Polym J.

[65] Bulut U, Crivello JV (2005). Investigation of the reactivity of epoxide monomers in photoinitiated cationic polymerization. Macromolecules.

[66] Decker C (2002). Kinetic study and new applications of UV radiation curing. Macromol Rapid Commun.

[67] Shimomoto H, Fukami D, Kanaoka S, Aoshima S (2011). Fluorinated vinyl ether homopolymers and copolymers: living cationic polymerization and temperature-induced solubility transitions in various organic solvents including perfluoro solvents. J Polym Sci Part A Polym Chem.

[68] Guyot B, Améduri B, Boutevin B (1999). Cinétique de polymérisation radicalaire de (méth)acrylates à chaîne latérale fluorée. Macromol Chem Phys.

[69] Bonneaud C, Burgess JM, Bongiovanni R, Joly-Duhamel C, Friesen CM (2019). Photopolymerization of maleimide perfluoropolyalkylethers without a photoinitiator. J Polym Sci Part A Polym Chem.

[70] Friesen CM, Hay KA, Howell JL, Nyvall DA (2006) Insulated perfluoropolyether alkyl alcohols. United States Pat. Appl Publ 1–6

[71] Tonelli C, Di Meo A, Fontana S, Russo A (2002). Perfluoropolyether functional oligomers: unusual reactivity in organic chemistry. J Fluor Chem.

[72] Tonelli C, Gavezotti P, Strepparola E (1999). Linear perfluoropolyether difunctional oligomers: Chemistry, properties and applications. J Fluor Chem.

[73] Crivello JV (1999). The discovery and development of onium salt cationic photoinitiators. J Polym Sci Part A Polym Chem.

[74] Decker C (1996). Photoinitiated crosslinking polymerisation. Prog Polym Sci.

[75] Game P, Sage D, Chapel JP (2002). Surface mobility of polyurethane networks containing fluorinated amphiphilic reactive additives. Macromolecules.

[76] Esteves ACC, Lyakhova K, Van Der Ven LGJ (2013). Surface segregation of low surface energy polymeric dangling chains in a cross-linked polymer network investigated by a combined experimental-simulation approach. Macromolecules.

[77] Howell JL, Friesen CM, Shtarov AB, Thrasher JS, Waterfeld A, Pérez EW, Sullivan JF (2007). Improved thermal stability of perfluoropolyalkylethers (PFPAEs). J Synth Lubr.

[78] Kasai PH, Wheeler P (1991). Degradation of perfluoropolyethers catalyzed by aluminum chloride. Appl Surf Sci.

[79] Lopez G, Ameduri B, Habas JP (2016). A versatile strategy to synthesize perfluoropolyether-based thermoplastic fluoropolymers by alkyne-azide step-growth polymerization. Macromol Rapid Commun.

[80] Priola A, Bongiovanni R, Malucelli G, Pollicino A, Tonelli C, Simeone G (1997). UV-curable systems containing perfluoropolyether structures: synthesis and characterisation. Macromol Chem Phys.

[81] Tonelli C, Ajroldi G, Marigo A, Marega C, Turturro A (2001). Synthesis methods of fluorinated polyurethanes. 2. Effects on morphology and microstructure. Polymer (Guildf).

[82] Ameduri B, Bongiovanni R, Malucelli G, Pollicino A, Priola A (1999). New fluorinated acrylic monomers for the surface modification of UV-curable systems. J Polym Sci Part A Polym Chem.

[83] Zhang L, Zhou Z, Cheng B, DeSimone JM, Samulski ET (2006). Superhydrophobic behavior of a perfluoropolyether lotus-leaf-like topography. Langmuir.

[84] Samaha MA, Tafreshi HV, Gad-El-Hak M (2012). Influence of flow on longevity of superhydrophobic coatings. Langmuir.

