# Supplementary material for: Novel perfluoropolyalkylethers monomers: synthesis and photo-induced cationic polymerization
Source: Colloid Polym Sci. 2021 Apr 25;299(7):1173–88. doi: 10.1007/s00396-021-04838-1 (PMC8550325; doi:10.1007/s00396-021-04838-1)
Supplement: Supplementary file 1 — (DOCX 2980 KB) [file 396_2021_4838_MOESM1_ESM.docx]

**Supporting Information**

Novel Perfluoropolyalkylethers monomers: synthesis and photo-induced cationic polymerization

Giuseppe Trusiano,^†,*^ Alessandra Vitale,^†,*^ Jason Pulfer,^§^ Josiah Newton,^§,#^ Christine Joly-Duhamel,^‡^ Chadron M. Friesen,^§^ Roberta Bongiovanni^†^

*^†^* Politecnico di Torino, Department of Applied Science and Technology, Corso Duca degli Abruzzi 24, 10129 Torino, Italy

*^§^* Trinity Western University, Department of Chemistry, 22500 University Drive, Langley City, BC V2Y 1Y1

*^#^* Simon Fraser University, Department of Chemistry, 8888 University Drive, BC V5A 1S6 Burnaby, Canada

*^‡^* University of Montpellier, Institut Charles Gerhardt Montpellier, CNRS, ENSCM, Cedex 5, 34095 Montpellier, France

* Corresponding authors: [giuseppe.trusiano@polito.it](mailto:giuseppe.trusiano@polito.it); [alessandra.vitale@polito.it](mailto:alessandra.vitale@polito.it)

**Table of Contents**

[S1. Experimental set-up for the PFPAE diacyl fluoride functionalization reaction with vinyl ethers: 4](#_Toc64547643)

[S2. ^1^H-NMR (400 MHz, benzene-*d_6_*, 25 °C) spectrum of PFPAE-EGVE 5](#_Toc64547644)

[S3. ^13^C-NMR (101 MHz, benzene-*d_6_*, 25 ^o^C) spectrum of PFPAE-EGVE 6](#_Toc64547645)

[S4. ^19^F-NMR (376.5 MHz, benzene-*d_6_*, 25 °C) spectrum of PFPAE-EGVE 7](#_Toc64547646)

[S5. PFPAE-EGVE: Calculation of the percentages of the monofunctional and difunctional oligomers and their molecular weight 8](#_Toc64547647)

[S6. Mass spectrum of PFPAE- EGVE 9](#_Toc64547648)

[S7. ^1^H-NMR (400 MHz, benzene-*d_6_* capillary, 25 °C) spectrum of PFPAE-BGVE 10](#_Toc64547649)

[S8. ^13^C-NMR (101 MHz, benzene-*d_6_*, 25 ^o^C) spectrum of PFPAE-BGVE 11](#_Toc64547650)

[S9. ^19^F-NMR (376.5 MHz, benzene-*d_6_*, 25 °C) spectrum of PFPAE-BGVE 12](#_Toc64547651)

[S10. PFPAE-BGVE: Calculation of the percentages of the monofunctional and difunctional oligomers and their molecular weight 13](#_Toc64547652)

[S11. Mass spectrum of PFPAE- BGVE 14](#_Toc64547653)

[S12. ^1^H-NMR (400 MHz, benzene-*d_6_* capillary, 25 °C) spectrum of PFPAE-DEGVE 15](#_Toc64547654)

[S13. ^13^C-NMR (101 MHz, benzene-*d_6_*, 25 ^o^C) spectrum of PFPAE-DEGVE 16](#_Toc64547655)

[S14. ^19^F-NMR (376.5 MHz, benzene-*d_6_*, 25 °C) spectrum of PFPAE-DEGVE 17](#_Toc64547656)

[S15. PFPAE-DEGVE: Calculation of the percentages of the monofunctional and difunctional oligomers and their molecular weight 18](#_Toc64547657)

[S16. Mass spectrum of PFPAE- DEGVE 19](#_Toc64547658)

[S17. ^1^H-NMR (400 MHz, dichloromethane-*d_2_*, 25 °C) spectrum of (2-bromomethyl)oxirane 20](#_Toc64547659)

[S18. ^1^H-NMR (400 MHz, chloroform-*d*, 25 °C) spectrum of (3-bromopropyl)oxirane 21](#_Toc64547660)

[S19. Experimental set-up for the PFPAE dicarboxylic acid functionalization reaction with epoxides: 22](#_Toc64547661)

[S20. ^1^H-NMR (400 MHz, benzene-d_6_ capillary, 25 °C) spectrum of PFPAE-MO 23](#_Toc64547662)

[S21. ^13^C-NMR (101 MHz, benzene-*d_6_*, 25 ^o^C) spectrum of PFPAE-MO 24](#_Toc64547663)

[S22. ^19^F-NMR (376.5 MHz, benzene-*d_6_*, 25 °C) spectrum of PFPAE-MO 25](#_Toc64547664)

[S23. PFPAE-MO: Calculation of the percentages of the monofunctional and difunctional oligomers and their molecular weight 26](#_Toc64547665)

[S24. Mass spectrum of PFPAE-MO 27](#_Toc64547666)

[S25. ^1^H-NMR (400 MHz, benzene-*d_6_* capillary, 25 °C) spectrum of PFPAE-EO 28](#_Toc64547667)

[S26. ^13^C-NMR (101 MHz, benzene-*d_6_*, 25 ^o^C) spectrum of PFPAE-EO 29](#_Toc64547668)

[S27. ^19^F-NMR (376.5 MHz, benzene-*d_6_*, 25 °C) spectrum of PFPAE-EO 30](#_Toc64547669)

[S28. PFPAE-EO: Calculation of the percentages of the monofunctional and difunctional oligomers and their molecular weight 31](#_Toc64547670)

[S29. Mass spectrum of PFPAE-EO 32](#_Toc64547671)

[S30. ^1^H-NMR (400 MHz, benzene-*d_6_*, 25 °C) spectrum of PFPAE-PO 33](#_Toc64547672)

[S31. ^13^C-NMR (101 MHz, benzene-*d_6_*, 25 ^o^C) spectrum of PFPAE-PO 34](#_Toc64547673)

[S32. ^19^F-NMR (376.5 MHz, benzene-*d_6_*, 25 °C) spectrum of PFPAE-PO 35](#_Toc64547674)

[S33. PFPAE-PO: Calculation of the percentages of the monofunctional and difunctional oligomers and their molecular weight 36](#_Toc64547675)

[S34. Mass spectrum of PFPAE-PO 37](#_Toc64547676)

[S35. Preparation of the photocurable sample PFPAE-BGVE 38](#_Toc64547677)

[S36. Study of the polymerization kinetics by photo-DSC 43](#_Toc64547678)

[S37. Maximum rate of polymerization of the photocured fluoropolymers by photo-DSC. 43](#_Toc64547679)

[S38. Study of the photopolymerization reaction by ATR FT-IR 44](#_Toc64547680)

[S39. TGA: degradation temperatures of the UV-cured fluoropolymers 47](#_Toc64547681)

[S40. Water contact angle hysteresis measurements, on the air side, of the UV-cured polymers 47](#_Toc64547682)

#

# S1. Experimental set-up for the PFPAE diacyl fluoride functionalization reaction with vinyl ethers:

a) ice-bath temperature reaction with addition funnel and stirring; b) room temperature complete reaction; c) purification of the reaction mixture through flash chromatography; d) spots of the desired product (fractions # 2 and 3) on a TLC plate; e) purified product.


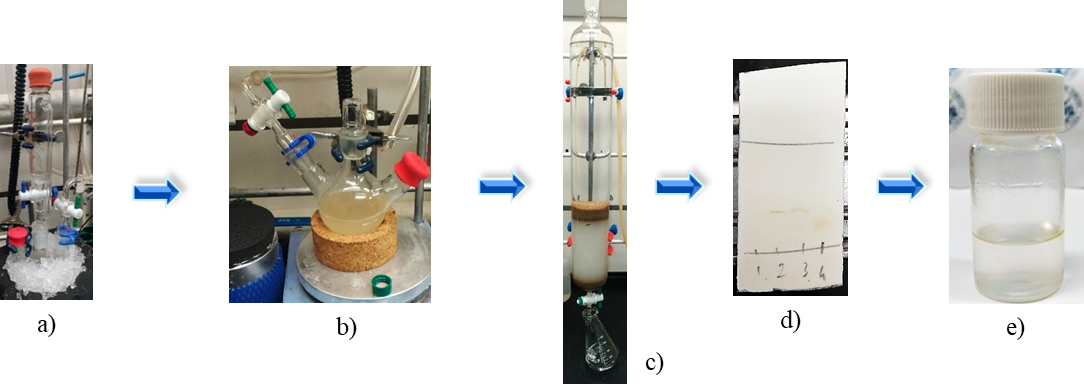


# S2. **^1^H-NMR (400 MHz, benzene-*d_6_*, 25 °C) spectrum of PFPAE-EGVE**

𝛿 = 6.66 (*dd*, -OC***H***CH_2_, ^3^*J_cis_* = 14.4 Hz, ^3^*J_trans_* = 6.8 Hz, 1H), 4.81 (*d*, -C(O)OC***H_2_***CH_2_-, *J* = 3.7 Hz, 2H), 4.40 (*d*, -OCH*=*C***H_trans_***H_cis_, ^3^*J*_cis_ *=* 15.3 Hz, 1H), 4.28 (*d,* -OCH*=*CH_trans_***H_cis_***, ^3^*J*_cis_ = 6.5 Hz, 1H), 4.16 (*s*, -C(O)OCH_2_C***H_2_***-, *J* = 5.3 Hz, 2H).


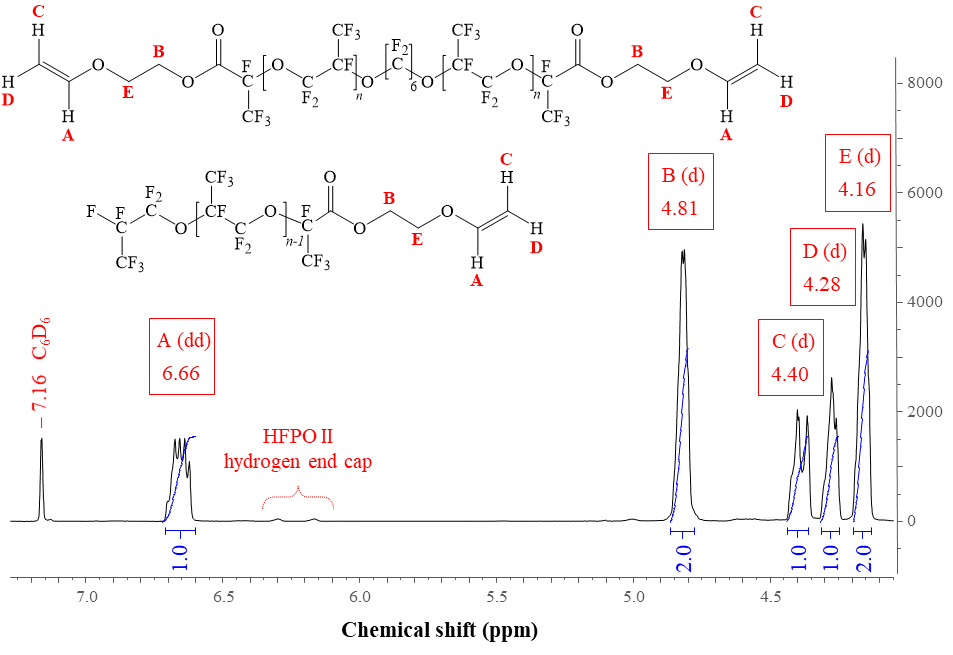


# S3. ^13^C-NMR (101 MHz, benzene-*d_6_*, 25 ^o^C) spectrum of PFPAE-EGVE

𝛿 = 158.53 (*s,* -***C***=O-), 151.14 (*s*, -O***C***HCH_2_), 122.49-99.17 (*m,* carbons of repeat unit), 86.92 (*s,* -OCH***C***H_2_), 66.16 (*s,* -***C***H_2_OCHCH_2_), 64.35 (*s*, -***C***H_2_CH_2_OCHCH_2_).


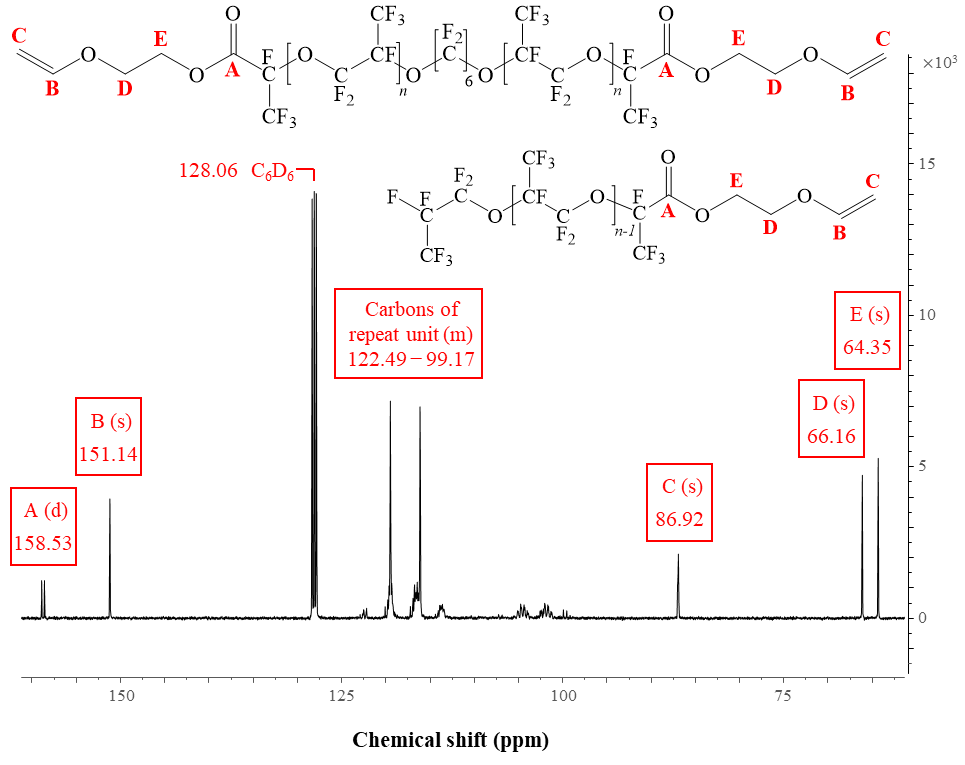


# S4. ^19^F-NMR (376.5 MHz, benzene-*d_6_*, 25 °C) spectrum of PFPAE-EGVE

𝛿 = -145.15 (*q*, C***F***(CF_3_) of repeat unit, mono and difunctional), -131.99 (*ω* C***F***(CF_3_), mono and difunctional), -130.23 (*s*, α C***F_2,_*** monofunctional), -125.67 (*s*, - CF_2_C***F_2_***CF_2_O-***_,_*** difunctional), -122.66 (*s*, - C***F_2_***CF_2_CF_2_O-***_,_*** difunctional), from -85.64 to -78.92 (CF_3_ and CF_2_ of repeat unit).


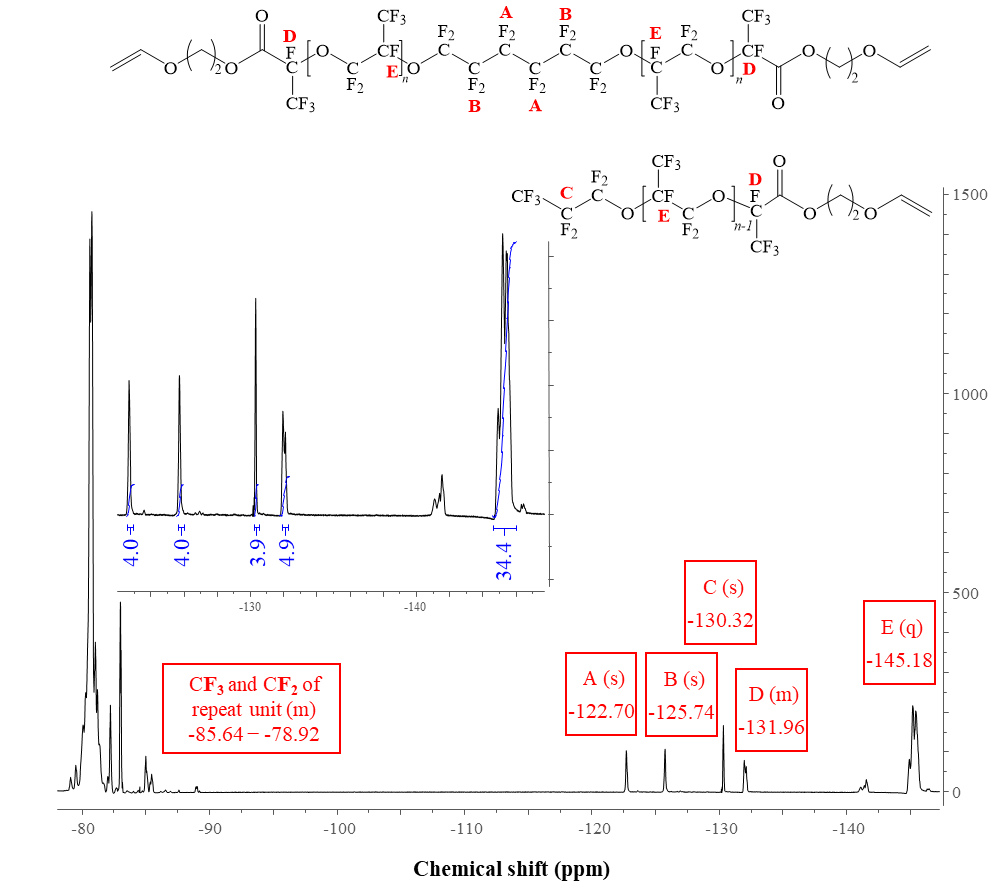


# S5. PFPAE-EGVE: Calculation of the percentages of the monofunctional and difunctional oligomers and their molecular weight

$$\text{\% }\text{di-}\text{PFPAE-EGVE}=\frac{\frac{{\int_{-131.99} \mathrm{CF}}}{{\int_{-130.23} \mathrm{CF}}_{2}}-\frac{1}{2}}{\frac{{\int_{-131.99} \mathrm{CF}}}{{\int_{-130.23} \mathrm{CF}}_{2}}+\frac{1}{2}}\times100=\frac{\frac{2.88}{1.65}-\frac{1}{2}}{\frac{2.88}{1.65}+\frac{1}{2}}\times100=55.47\%$$

$$f=\frac{\sum f_{i} {\%}_{i}}{\sum{\%}_{i}}=\frac{\left( 2\times55.47 \right)+\left( 1\times\left( 100-55.47 \right) \right)}{100}=1.55$$

$$n=\frac{{\int_{-145.15} \mathrm{CF}}}{{\int_{-131.99} \mathrm{CF}}}=\frac{16.97}{2.88}=5.88$$

$$M_{n (monofunctional chain)}=mass of \alpha unit + n\times(mass of repeat unit) + mass of \omega\mathrm{unit}$$

$$M_{n (monofunctional chain)}=18.99+5.88\times\left( 166.01 \right)+215.12=1210.25 g/mol$$

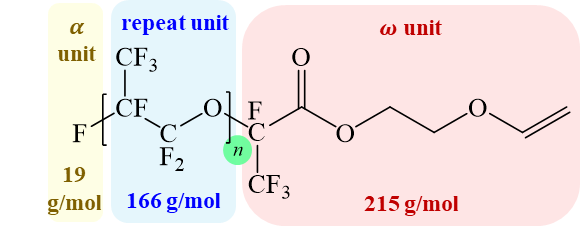


$M_{n (difunctional chain)}=$mass of $\alpha$ unit + $2\times$ $n\times$ (mass of repeat unit) + $2\times$mass of $\omega$ unit

$$M_{n (difunctional chain)}=332.04+2\times5.88\times\left( 166.01 \right)+2\times215.12=2714.56 g/mol$$

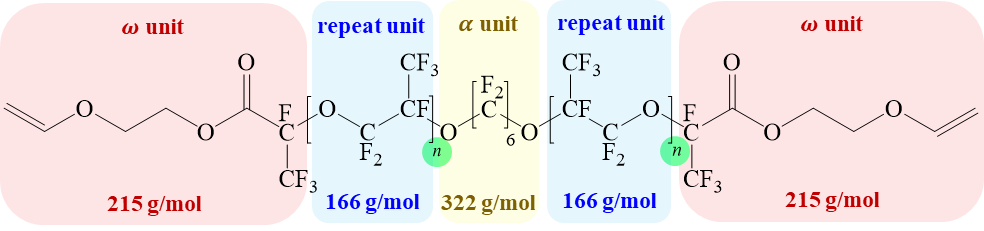


# S6. Mass spectrum of PFPAE- EGVE

GC–MS (EI) fragmentation: m/z = OCHCH_2_^+^ (43 m/z), CH_2_OCHCH_2_^+^ (57 m/z), CF_3_^+^ (69 m/z), CH_2_CH_2_OCHCH_2_^+^ (71 m/z), OCH_2_CH_2_OCHCH_2_^+^ (87 m/z), C_2_F_4_^+^ (100 m/z), C_2_F_5_^+^ (119 m/z), C_3_F_5_O^+^ (147 m/z), C_3_F_6_^+^ (150 m/z), C_3_F_7_^+^ (169 m/z), CF(CF_3_)C(O)OCH_2_CH_2_OCHCH_2_^+^ (215 m/z).


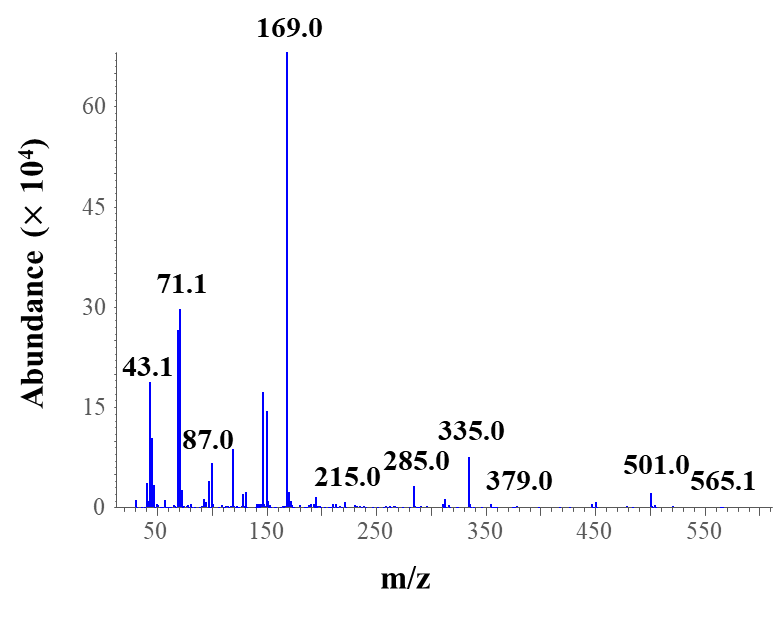


# S7. **^1^H-NMR (400 MHz, benzene-*d_6_* capillary, 25 °C) spectrum of PFPAE-BGVE**

𝛿 = 6.65 (*dd*, -OC**H**CH_2_, ^3^*J_cis_* = 14.4 Hz, ^3^*J_trans_* = 6.6 Hz, 1H), 4.67 (*s*, -C(O)OC***H_2_***CH_2_-, 2H), 4.35 (*d,* ^3^*J*_cis_ = 17.3 Hz, -OCH*=*C***H_trans_***H_cis_, 1H), 4.19 (*d,* ^3^*J*_cis_ *=* 11.1 Hz, -OCH*=*CH_trans_***H_cis_***, 1H), 3.93 (*s*, -C(O)OCH_2_CH_2_CH_2_C***H_2_***-, 2H), 2.14 (*s*, -C(O)OCH_2_C***H_2_***CH_2_CH_2_-, 2H), 2.03 (*s*, -C(O)OCH_2_CH_2_C***H_2_***CH_2_-, 2H).


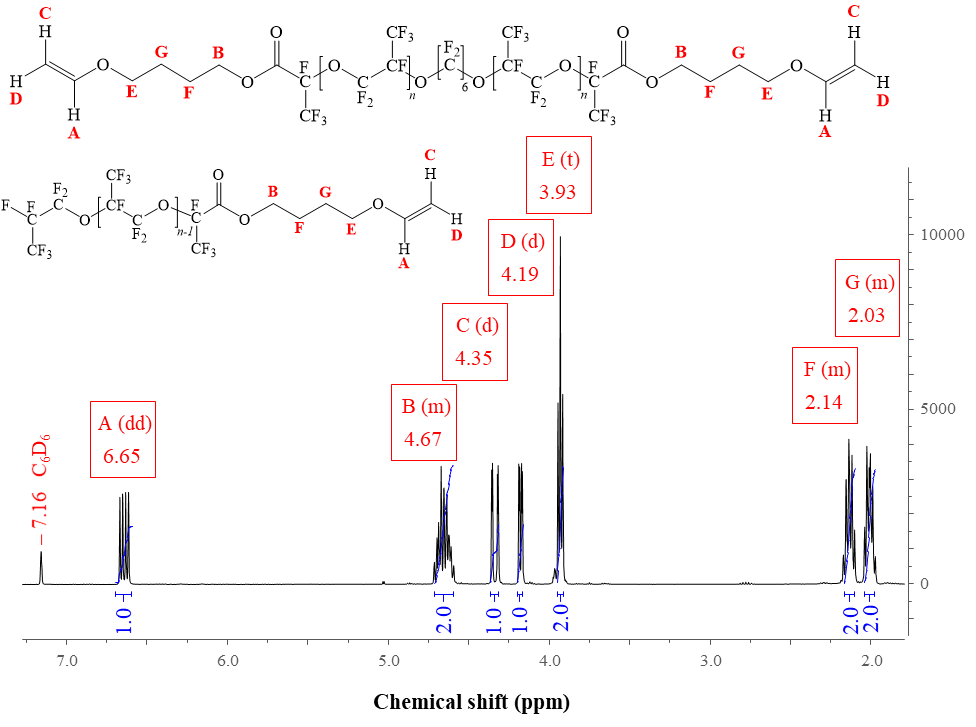


# S8. ^13^C-NMR (101 MHz, benzene-*d_6_*, 25 ^o^C) spectrum of PFPAE-BGVE

𝛿 = 158.46 (*d,* -***C***=O-), 151.74 (*s*, -O***C***HCH_2_), 122.47-99.58 (*m,* carbons of repeat unit), 86.19 (*s,* -OCH***C***H_2_), 68.31 (*s,* -***C***H_2_OCHCH_2_), 66.34 (*s*, -***C***H_2_CH_2_CH_2_CH_2_OCHCH_2_), 25.57 (*s*, -CH_2_CH_2_***C***H_2_CH_2_OCHCH_2_), 25.44 (*s*, -CH_2_***C***H_2_CH_2_CH_2_OCHCH_2_).


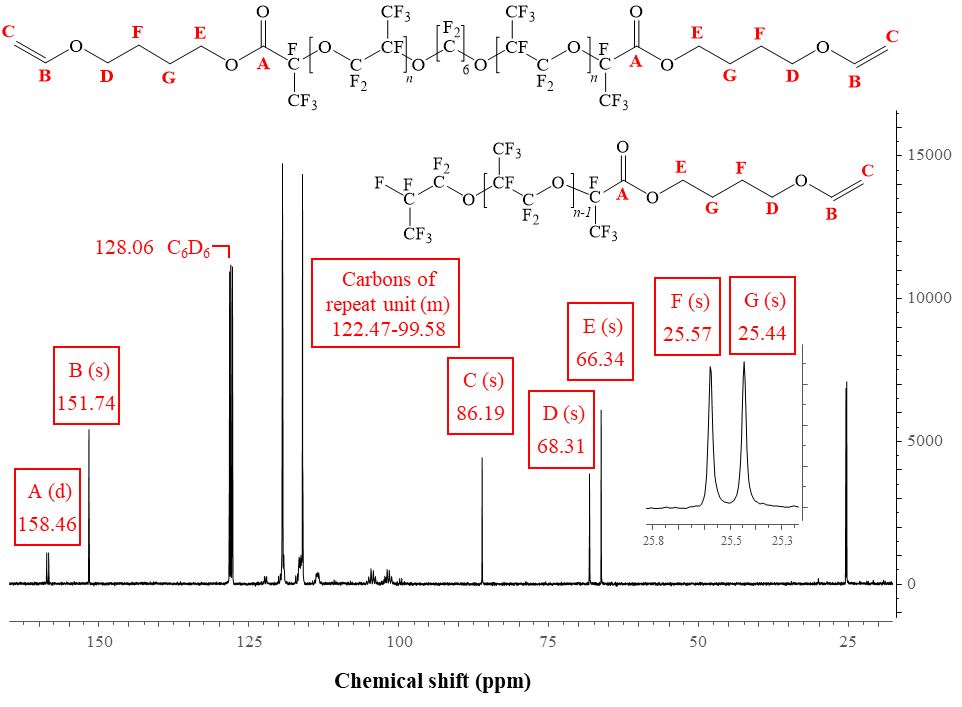


# S9. ^19^F-NMR (376.5 MHz, benzene-*d_6_*, 25 °C) spectrum of PFPAE-BGVE

𝛿 = -145.22 (*q*, C***F***(CF_3_) of repeat unit, mono and difunctional), -131.91 (*ω* C***F***(CF_3_), mono and difunctional), -130.29 (*s*, α C***F_2,_*** monofunctional), -125.73 (*s*, - CF_2_C***F_2_***CF_2_O-***_,_*** difunctional), -122.72 (*s*, -C***F_2_***CF_2_CF_2_O-***_,_*** difunctional), from -85.45 to -79.30 (CF_3_ and CF_2_ of repeat unit).

#
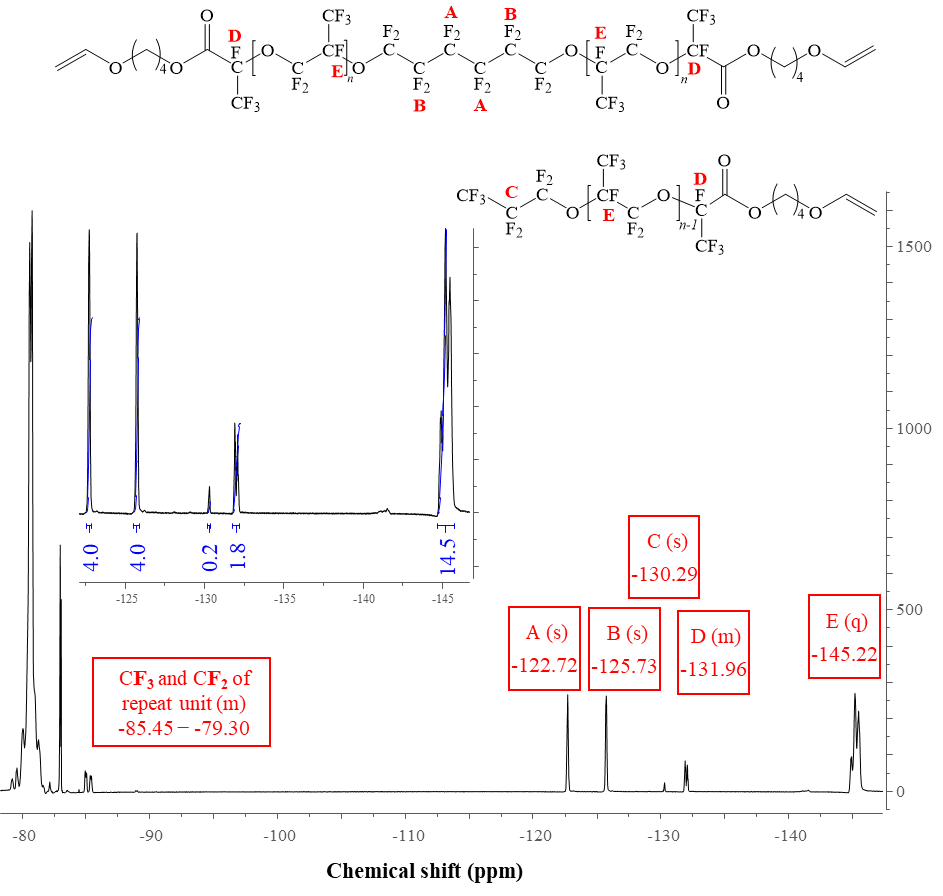
S10. PFPAE-BGVE: Calculation of the percentages of the monofunctional and difunctional oligomers and their molecular weight

$$\text{\% di-PFPAE-BGVE} =\frac{\frac{{\int_{-131.91} \mathrm{CF}}}{{\int_{-130.29} \mathrm{CF}}_{2}}-\frac{1}{2}}{\frac{{\int_{-131.91} \mathrm{CF}}}{{\int_{-130.29} \mathrm{CF}}_{2}}+\frac{1}{2}}\times100=\frac{\frac{1.84}{0.24}-\frac{1}{2}}{\frac{1.84}{0.24}+\frac{1}{2}}\times100=87.75\%$$

$$f=\frac{\sum f_{i} {\%}_{i}}{\sum{\%}_{i}}=\frac{\left( 2\times87.75 \right)+\left( 1\times\left( 100-87.75 \right) \right)}{100}=1.88$$

$$n=\frac{{\int_{-145.22} \mathrm{CF}}}{{\int_{-131.91} \mathrm{CF}}}=\frac{14.55}{1.84}=7.91$$

$$M_{n (monofunctional chain)}=mass of \alpha unit + n\times(mass of repeat unit) + mass of \omega\mathrm{unit}$$

$$M_{n (monofunctional chain)}=18.99+7.91\times\left( 166.01 \right)+243.18=1575.31 g/mol$$

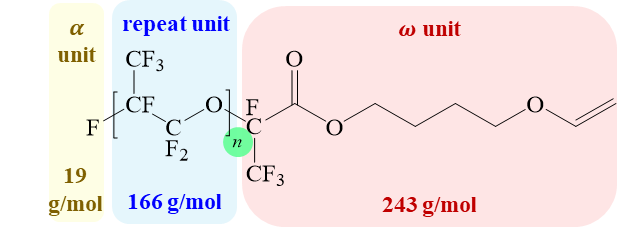


$M_{n (difunctional chain)}=$mass of $\alpha$ unit + $2\times$ $n\times$ (mass of repeat unit) + $2\times$mass of $\omega$ unit

$$M_{n (difunctional chain)}=332.04+2\times7.91\times\left( 166.01 \right)+2\times243.18=3444.68 g/mol$$

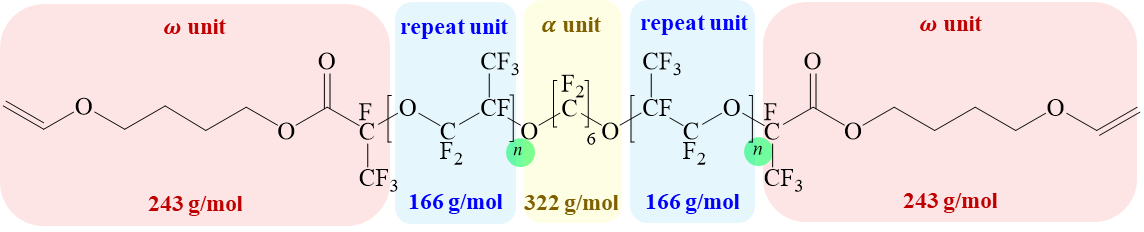


# S11. Mass spectrum of PFPAE- BGVE

GC–MS (EI) fragmentation: m/z = OCHCH_2_^+^ (43 m/z), CH_2_OCHCH_2_^+^ (57 m/z), CF_3_^+^ (69 m/z), CH_2_CH_2_OCHCH_2_^+^ (71 m/z), CH_2_CH_2_CH_2_OCHCH_2_^+^ (85 m/z), CH_2_CH_2_CH_2_CH_2_OCHCH_2_^+^ (99 m/z), C_2_F_4_^+^ (100 m/z), OCH_2_CH_2_CH_2_CH_2_OCHCH_2_^+^ (115 m/z), C_2_F_5_^+^ (119 m/z), C_3_F_5_O^+^ (147 m/z), C_3_F_6_^+^ (150 m/z), C_3_F_7_^+^ (169 m/z), CF(CF_3_)C(O)OCH_2_CH_2_CH_2_CH_2_OCHCH_2_^+^ (243 m/z).


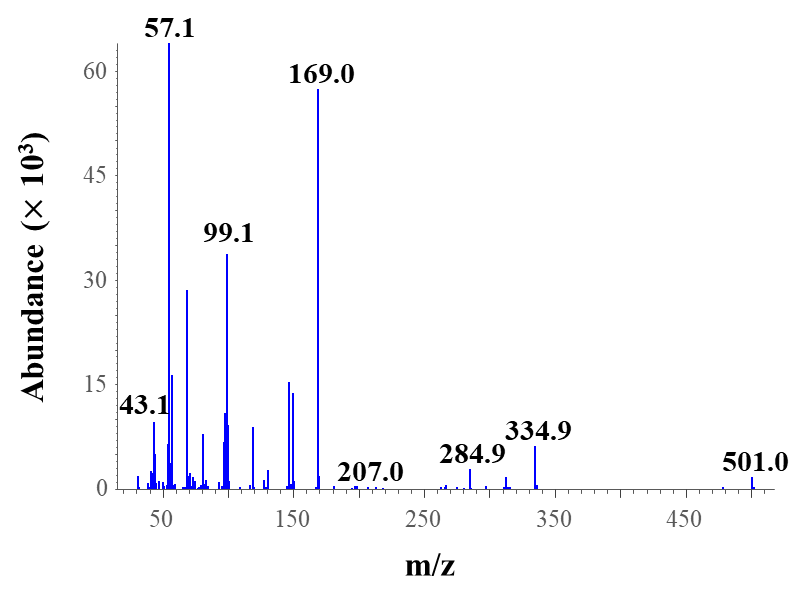


# S12. **^1^H-NMR (400 MHz, benzene-*d_6_* capillary, 25 °C) spectrum of PFPAE-DEGVE**

𝛿 = 6.64 (*dd*, -OC***H***CH_2_, ^3^*J_cis_* = 14.1 Hz, ^3^*J_trans_* = 7.3 Hz, 1H), 4.76 (*s*, -C(O)OC***H_2_***CH_2_O-, 2H), 4.39 (*d,* -OCH*=*C***H_trans_***H_cis_, ^3^*J*_cis_ = 14.1 Hz, 1H), 4.21 (*d*, -OCH*=*CH_trans_***H_cis_***, ^3^*J*_cis_ *=* 7.3 Hz, 1H), 4.03 (*s*, -C(O)OCH_2_C***H_2_***OC***H_2_***CH_2_O-, 4H), 3.95 (*s*, -OCH_2_C***H_2_***OCHCH_2_, 2H). Impurities at 6.28-6.15 ppm are attributable to HFPO oligomers having as end group H-CF_2_-, i.e. hydrogen end capped HFPO (HFPO HEC)


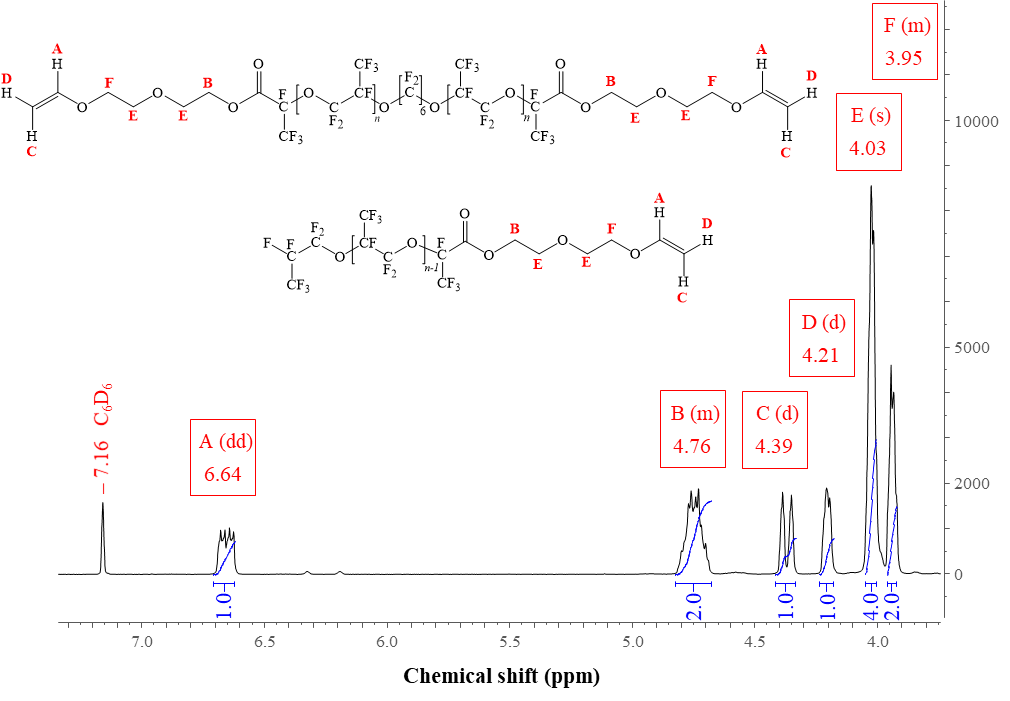


# S13. ^13^C-NMR (101 MHz, benzene-*d_6_*, 25 ^o^C) spectrum of PFPAE-DEGVE

𝛿 = 158.82 (*d*, -***C***=O-), 151.77 (*s*, -O***C***HCH_2_), 122.52-99.61 (*m*, carbons of repeat unit), 86.43 (*s*, -OCH***C***H_2_), 69.98 (*s, -****C***H_2_CH_2_OCHCH_2_), 68.59 (*s*, *-*C(O)OCH_2_***C***H_2_O-), 67.40 (*s*, -OCH_2_***C***H_2_OCHCH_2_), 67.32 (*s*, *-*C(O)O***C***H_2_CH_2_O-).


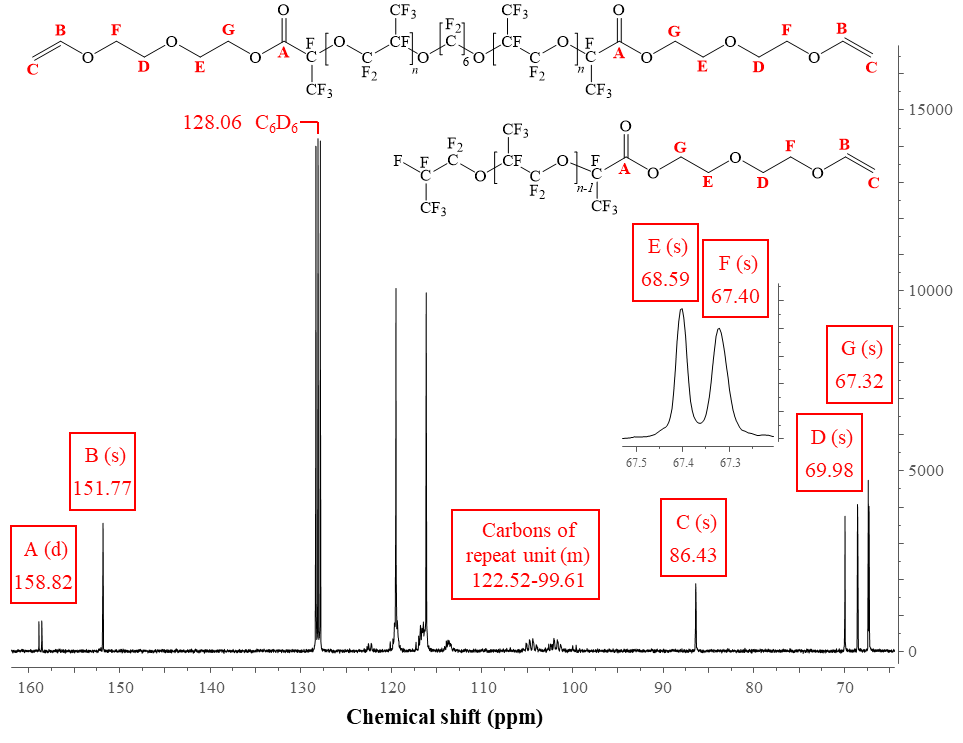


# S14. ^19^F-NMR (376.5 MHz, benzene-*d_6_*, 25 °C) spectrum of PFPAE-DEGVE

𝛿 = -145.18 (*q*, C***F***(CF_3_) of repeat unit, mono and difunctional), -131.96 (*ω* C***F***(CF_3_), mono and difunctional), -130.32 (*s*, α C***F_2,_*** monofunctional), -125.74 (*s*, - CF_2_C***F_2_***CF_2_O-***_,_*** difunctional), -122.70 (*s*, -C***F_2_***CF_2_CF_2_O-***_,_*** difunctional), from -85.48 to -79.10 (CF_3_ and CF_2_ of repeat unit).

**
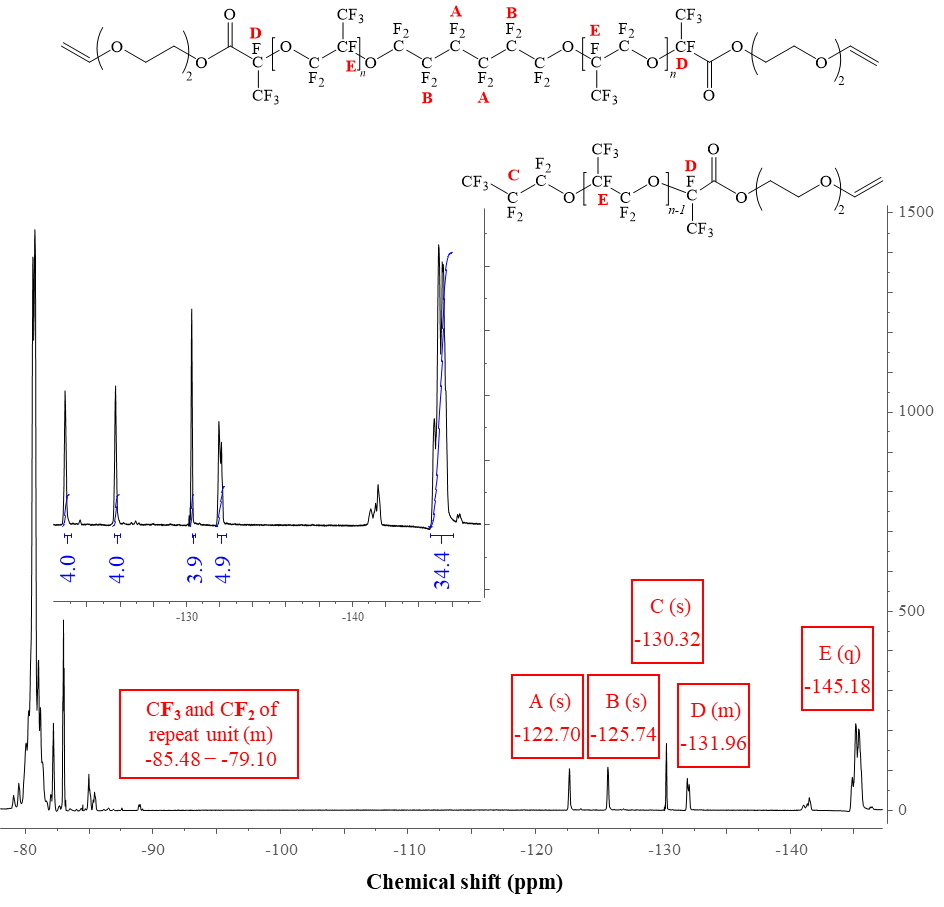
**

# **S15. PFPAE-DEGVE:** Calculation of the percentages of the monofunctional and difunctional oligomers and their molecular weight

$$\text{\% di-PFPAE-DEGVE}=\frac{\frac{{\int_{-131.96} \mathrm{CF}}}{{\int_{-130.32} \mathrm{CF}}_{2}}-\frac{1}{2}}{\frac{{\int_{-131.96} \mathrm{CF}}}{{\int_{-130.32} \mathrm{CF}}_{2}}+\frac{1}{2}}\times100=\frac{\frac{4.97}{3.98}-\frac{1}{2}}{\frac{4.97}{3.98}+\frac{1}{2}}\times100=42.82\%$$

$$f=\frac{\sum f_{i} {\%}_{i}}{\sum{\%}_{i}}=\frac{\left( 2\times42.82 \right)+\left( 1\times\left( 100-42.82 \right) \right)}{100}=1.43$$

$$n=\frac{{\int_{-145.18} \mathrm{CF}}}{{\int_{-131.96} \mathrm{CF}}}=\frac{34.44}{4.97}=6.93$$

$$M_{n (monofunctional chain)}=mass of \alpha unit + n\times(mass of repeat unit) + mass of \omega\mathrm{unit}$$

$$M_{n (monofunctional chain)}=18.99+6.93\times\left( 166.01 \right)+259.18=1428.62 g/mol$$

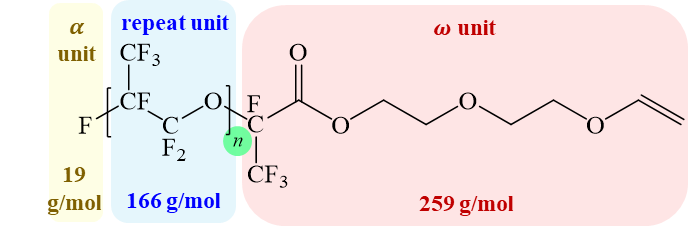


$M_{n (difunctional chain)}=$mass of $\alpha$ unit + $2\times$ $n\times$ (mass of repeat unit) + $2\times$mass of $\omega$ unit

$$M_{n (difunctional chain)}=332.04+2\times6.93\times\left( 166.01 \right)+2\times259.18=3151.3 g/mol$$

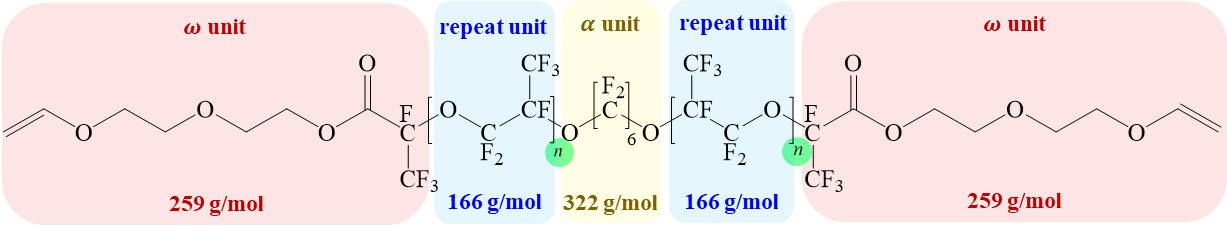


# S16. Mass spectrum of PFPAE- DEGVE

GC–MS (EI) fragmentation: m/z = OCHCH_2_^+^ (43 m/z), CH_2_OCHCH_2_^+^ (57 m/z), CF_3_^+^ (69 m/z), CH_2_CH_2_OCHCH_2_^+^ (71 m/z), OCH_2_CH_2_OCHCH_2_^+^ (87 m/z), C_2_F_4_^+^ (100 m/z), CH_2_CH_2_OCH_2_CH_2_OCHCH_2_^+^ (115 m/z), C_2_F_5_^+^ (119 m/z), C_3_F_5_O^+^ (147 m/z), C_3_F_6_^+^ (150 m/z), C_3_F_7_^+^ (169 m/z), CF(CF_3_)C(O)OCH_2_CH_2_OCH_2_CH_2_OCHCH_2_^+^ (259 m/z).


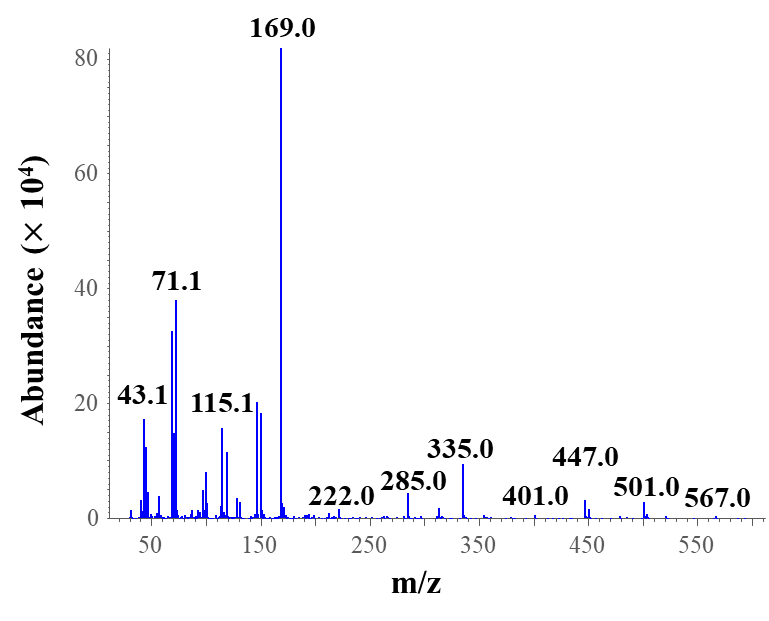


# S17. ^1^H-NMR (400 MHz, dichloromethane-*d_2_*, 25 °C) spectrum of (2-bromomethyl)oxirane

for reference of change in chemical shift upon formation of PFPAE-EO

*δ* = 3.50 (*t*, Br-C***H_2_***-CH_2_CH(O)CH_2_, 2H), 3.06 (*m,* -CH(O)C***H_b_***H_d_, 1H), 2.80 (*t,* -C***H***(O)CH_2_, 1H), 2.56 (*m,* -CH(O)CH_b_***H_d_***, 1H), 2.13 (*m,* Br-CH_2_-C***H_e_***H_f_CH(O)CH_2_, 1H), 2.05 (*m,* Br-CH_2_-CH_e_***H_f_***CH(O)CH_2_, 1H).


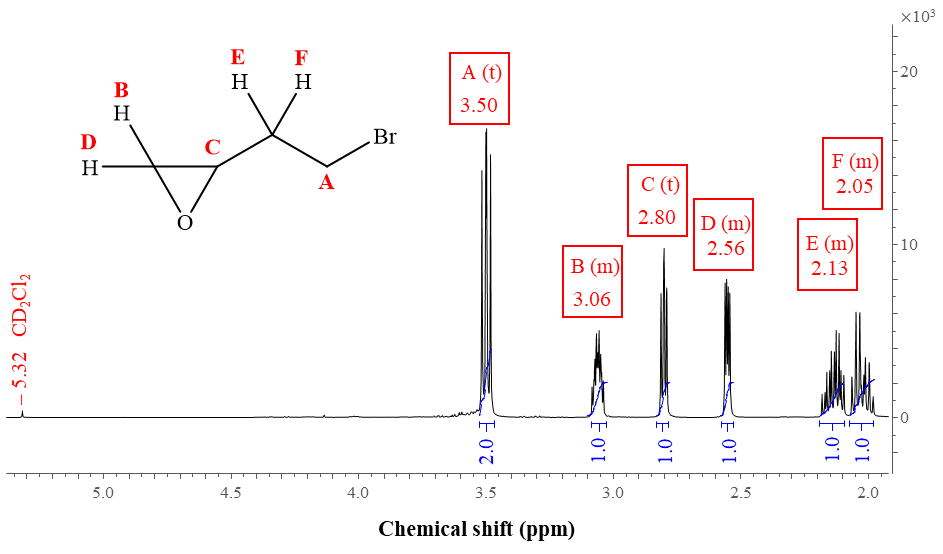


# S18. ^1^H-NMR (400 MHz, chloroform-*d*, 25 °C) spectrum of (3-bromopropyl)oxirane

for reference of change in chemical shift upon formation of PFPAE-PO

*δ* = 3.42 (*m*, Br-C***H_2_***-CH_2_CH_2_-, 2H), 2.89 (*m,* -CH(O)C***H_b_***H_d_, 1H), 2.72 (*t,* -C***H***(O)CH_2_, 1H), 2.47 (*m,* -CH(O)CH_b_***H_d_***, 1H), 1.99 (*m*, BrCH_2_-C***H_2_***-CH_2_-, 2H), 1.78 (*m,* Br-CH_2_CH_2_-C***H_f_***H_g_CH(O)CH_2_, 1H), 1.56 (*m,* Br-CH_2_CH_2_-CH_f_***H_g_***CH(O)CH_2_, 1H).


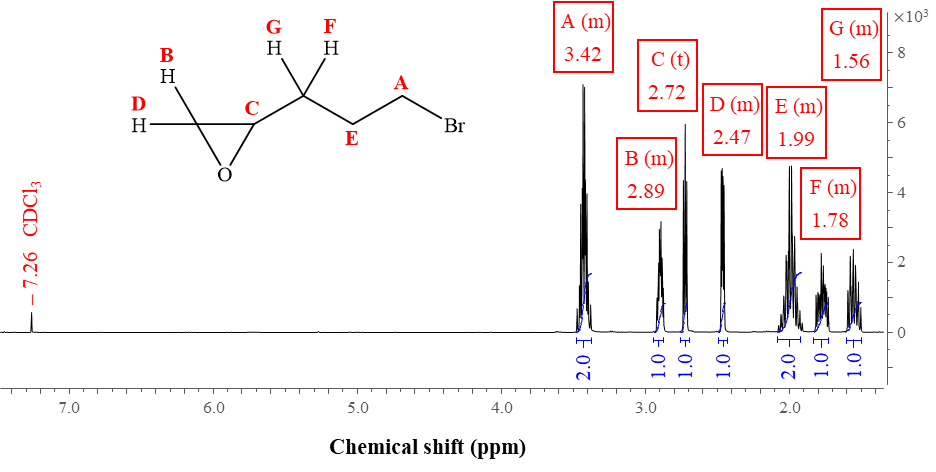


# S19. **Experimental set-up for the PFPAE dicarboxylic acid functionalization reaction with epoxides:**

1. room temperature silver oxide in MeCN:PFB solution with stirring; b) complete reaction; c) purification of the reaction mixture through flash chromatography; d) spots of the desired product (fractions # 3 and 4) on a TLC plate; e) purified product.


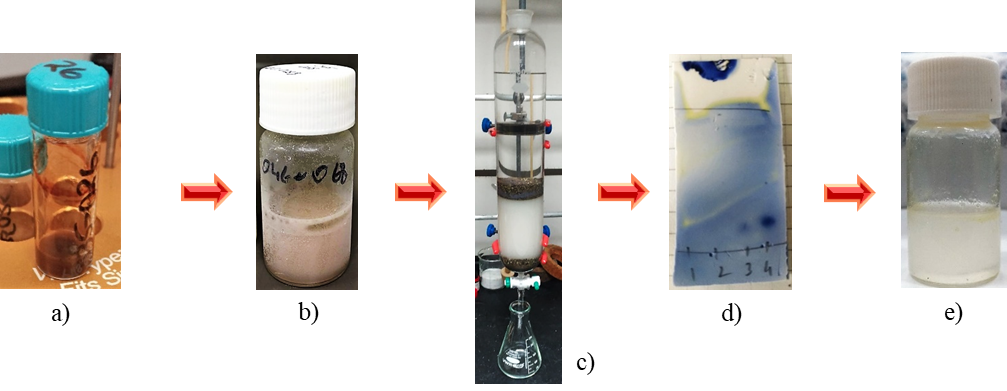


# S20. ^1^H-NMR (400 MHz, benzene-d_6_ capillary, 25 °C) spectrum of PFPAE-MO

𝛿 = 4.77 (*m*, -OC***H_a_***H_b_-CH(O)CH_2_, 1H), 4.45 (*m*, -OCH_a_***H_b_***-CH(O)CH_2_, 1H), 3.36 (*s,* -C***H***(O)CH_2_, 1H), 2.99 (*s,* -CH(O)C***H_d_***H_e_, 1H), 2.80 (*s,* -CH(O)CH_a_***H_e_***, 1H). Impurities at 6.28-6.15 ppm are attributable to HFPO oligomers having as end group H-CF_2_- i.e. hydrogen end capped HFPO (HFPO HEC)


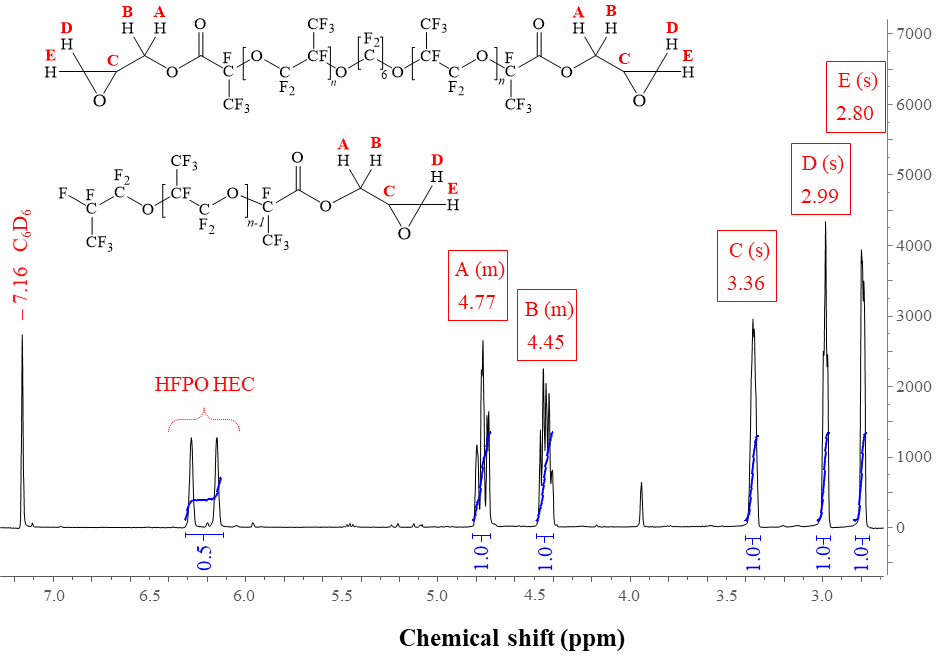


# S21. ^13^C-NMR (101 MHz, benzene-*d_6_*, 25 ^o^C) spectrum of PFPAE-MO

𝛿 = 158.78 (*d*, -***C***=O-), from 122.40 to 95.46 (*m*, −***C***F_3_ and ***C***F_2_ of repeat unit), 68.90 (*d*, -***C***H_2_CH(O)CH_2_), 47.53 (*s*, -CH_2_***C***H(O)CH_2_), 43.70 (*s*, -CH_2_CH(O)***C***H_2_).


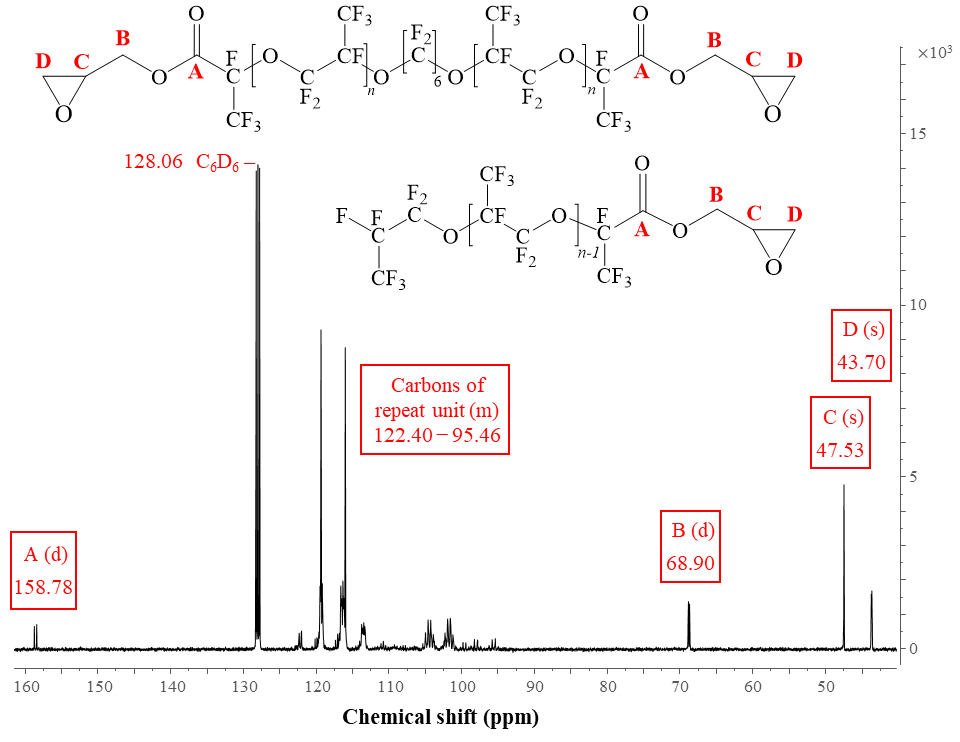


# S22. ^19^F-NMR (376.5 MHz, benzene-*d_6_*, 25 °C) spectrum of PFPAE-MO

*δ* = -146.68 (*s*, HFPO II hydrogen end cap), -145.38 (*q*, C***F***(CF_3_) of repeat unit, mono and difunctional), -132.16 (*ω* C***F***(CF_3_), mono and difunctional), -130.50 (*s*, α C***F_2,_*** monofunctional), -125.91 (*s*, -CF_2_C***F_2_***CF_2_O-***_,_*** difunctional), -122.85 (*s*, -C***F_2_***CF_2_CF_2_O-***_,_*** difunctional), from -87.17 to -79.13 (C***F_3_*** and C***F_2_*** of repeat unit).


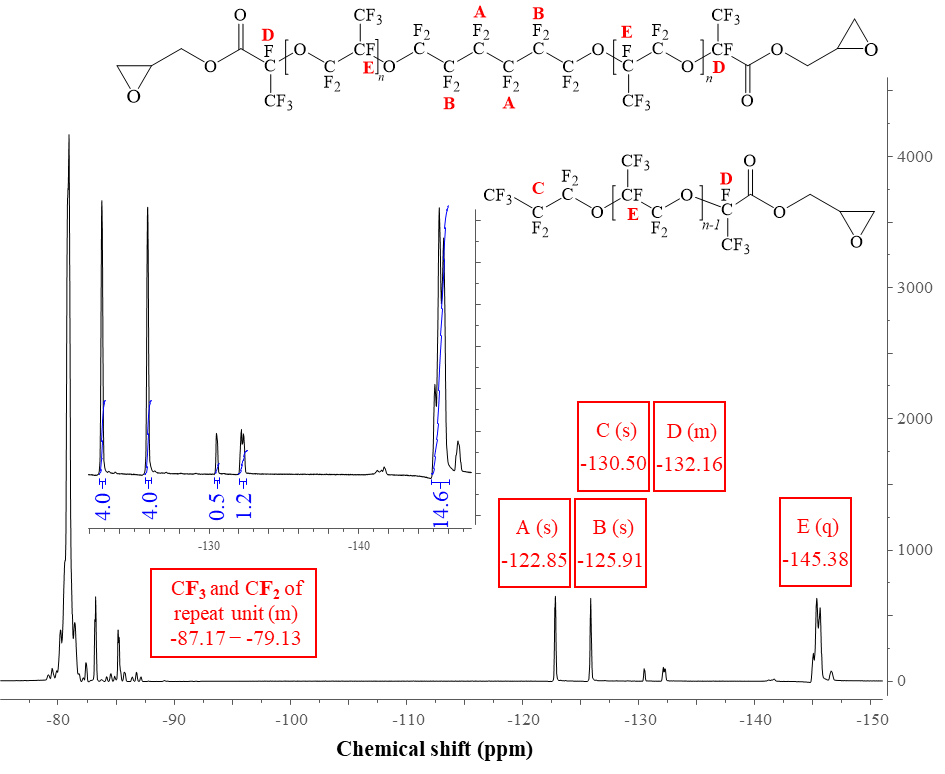


# S23. PFPAE-MO: Calculation of the percentages of the monofunctional and difunctional oligomers and their molecular weight

$$\text{\% di-PFPAE-MO}=\frac{\frac{{\int_{-132.16} \mathrm{CF}}}{{\int_{-130.50} \mathrm{CF}}_{2}}-\frac{1}{2}}{\frac{{\int_{-132.16} \mathrm{CF}}}{{\int_{-130.50} \mathrm{CF}}_{2}}+\frac{1}{2}} \times100=\frac{\frac{1.22}{0.56}-\frac{1}{2}}{\frac{1.22}{0.56}+\frac{1}{2}}\times100=62.67\%$$

$$f=\frac{\sum f_{i} {\%}_{i}}{\sum{\%}_{i}}=\frac{\left( 2\times62.67 \right)+\left( 1\times\left( 100-62.67 \right) \right)}{100}=1.63$$

$$n=\frac{{\int_{-145.38} \mathrm{CF}}}{{\int_{-132.16} \mathrm{CF}}}=\frac{14.60}{1.22}=11.97$$

$$M_{n (monofunctional chain)}=mass of \alpha unit + n\times(mass of repeat unit) + mass of \omega\mathrm{unit}$$

$$M_{n (monofunctional chain)}=18.99+11.97\times\left( 166.01 \right)+201.09=2207.22 g/mol$$

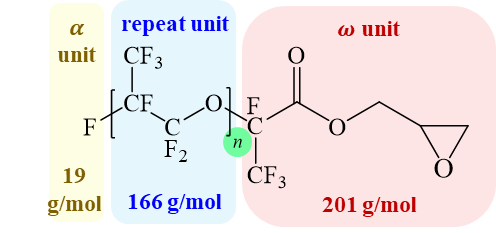


$M_{n (difunctional chain)}=$mass of $\alpha$ unit + $2\times$ $n\times$ (mass of repeat unit) + $2\times$mass of $\omega$ unit

$$M_{n (difunctional chain)}=332.04+2\times11.97\times\left( 166.01 \right)+2\times201.09=4708.5 g/mol$$

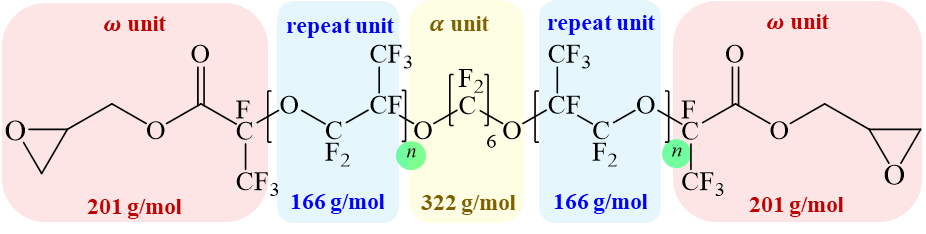


# S24. Mass spectrum of PFPAE-MO

GC–MS (EI) fragmentation: m/z = CHOCH_2_^+^ (43 m/z), CH_2_CHOCH_2_^+^ (57 m/z), CF_3_^+^ (69 m/z), OCH_2_CHOCH_2_^+^ (73 m/z), C_2_F_4_^+^ (100 m/z), C_2_F_5_^+^ (119 m/z), C_3_F_5_O^+^ (147 m/z), C_3_F_6_^+^ (150 m/z), C_3_F_7_^+^ (169 m/z), CF(CF_3_)C(O)OCH_2_CHOCH_2_^+^ (201 m/z).


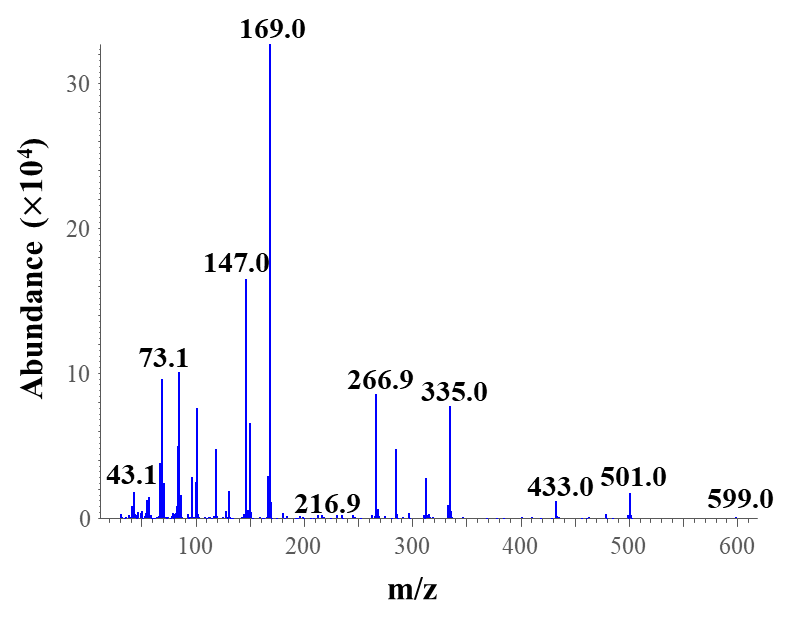


# S25. **^1^H-NMR (400 MHz, benzene-*d_6_* capillary, 25 °C) spectrum of PFPAE-EO**

𝛿 = 4.75 (*m*, -OC***H_2_***-CH_2_-, 2H), 3.12 (*s,* -C***H***(O)CH_2_, 1H), 2.92 (*s,* -CH(O)C***H_c_***H_d_, 1H), 2.64 (*s,* -CH(O)CH_c_***H_d_***, 1H), 2.29 (*m, -*CH_2_-C***H_e_***H_f_-CH(O)CH_2_, 1H), 2.04 (*m,* -CH_2_- CH_e_***H_f_***-CH(O)CH_2_, 1H). Impurities at 6.28-6.15 ppm are attributable to HFPO oligomers having as end group H-CF_2_- i.e. hydrogen end capped HFPO (HFPO HEC)


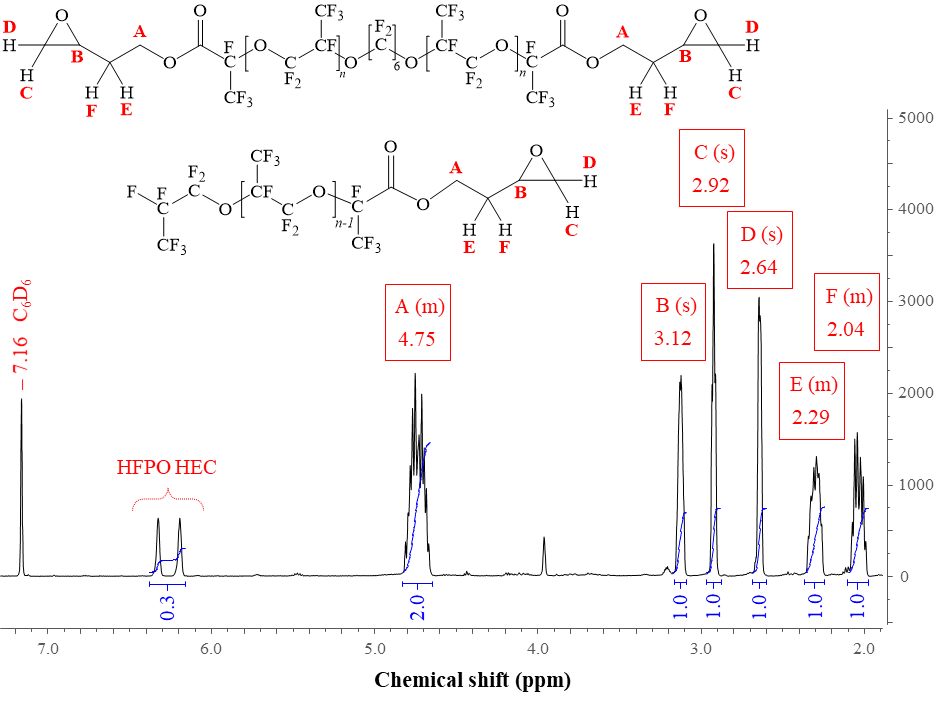


# S26. ^13^C-NMR (101 MHz, benzene-*d_6_*, 25 ^o^C) spectrum of PFPAE-EO

𝛿 = 158.74 (*d*, -***C***=O-), from 122.45 to 95.51 (*m*, −***C***F_3_ and ***C***F_2_ of repeat unit), 65.53 (*s*, -O***C***H_2_CH_2_CH(O)CH_2_), 47.91 (*s*, -***C***H(O)CH_2_), 46.05 (*s*, -CH(O)***C***H_2_), 31.76 (*s*, -CH_2_***C***H_2_CH(O)CH_2_).


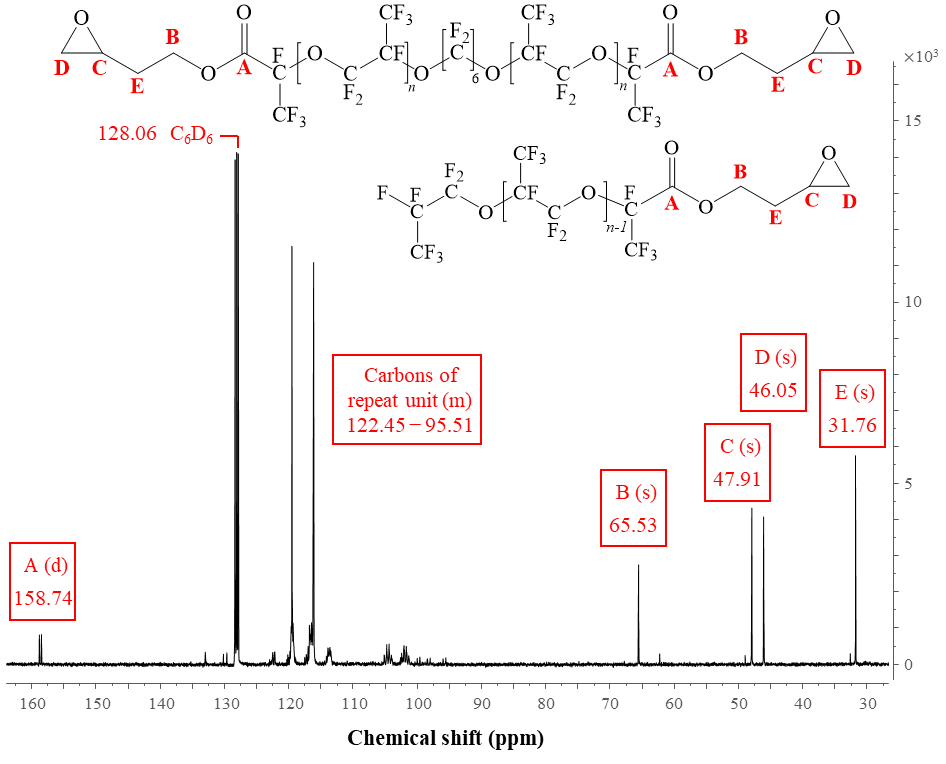


# S27. ^19^F-NMR (376.5 MHz, benzene-*d_6_*, 25 °C) spectrum of PFPAE-EO

𝛿 = -146.55 (*s*, HFPO II hydrogen end cap), -145.25 (*q*, C***F***(CF_3_) of repeat unit, mono and difunctional), -132.01 (*ω* C***F***(CF_3_), mono and difunctional), -130.38 (*s*, α C***F_2,_*** monofunctional), -125.78 (*s*, -CF_2_C***F_2_***CF_2_O-***_,_*** difunctional), -122.75 (*s*, -C***F_2_***CF_2_CF_2_O-***_,_*** difunctional), from -87.01 to -79.21 (CF_3_ and CF_2_ of repeat unit).


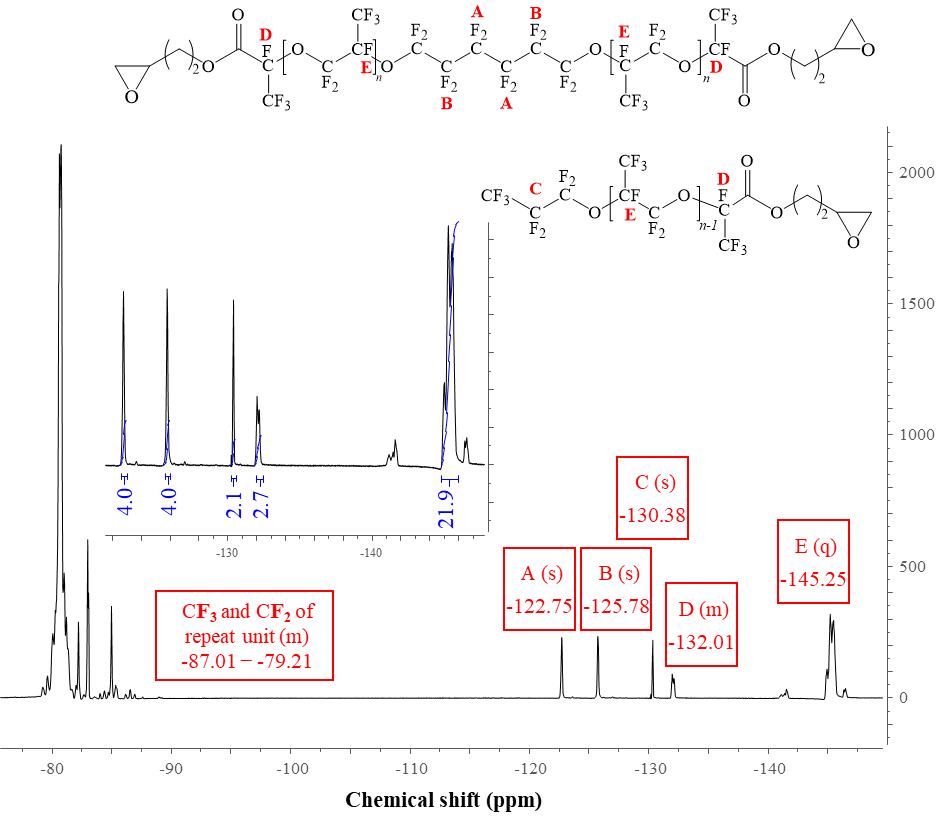


# S28. PFPAE-EO: Calculation of the percentages of the monofunctional and difunctional oligomers and their molecular weight

$$\text{\% di-PFPAE-EO}=\frac{\frac{{\int_{-132.01} \mathrm{CF}}}{{\int_{-130.38} \mathrm{CF}}_{2}}-\frac{1}{2}}{\frac{{\int_{-132.01} \mathrm{CF}}}{{\int_{-130.38} \mathrm{CF}}_{2}}+\frac{1}{2}}\times100=\frac{\frac{2.77}{2.23}-\frac{1}{2}}{\frac{2.77}{2.23}+\frac{1}{2}}\times100=42.60\%$$

$$f=\frac{\sum f_{i} {\%}_{i}}{\sum{\%}_{i}}=\frac{\left( 2\times42.60 \right)+\left( 1\times\left( 100-42.60 \right) \right)}{100}=1.43$$

$$n=\frac{{\int_{-145.25} \mathrm{CF}}}{{\int_{-132.01} \mathrm{CF}}}=\frac{21.97}{2.77}=7.93$$

$$M_{n (monofunctional chain)}=mass of \alpha unit + n\times(mass of repeat unit) + mass of \omega\mathrm{unit}$$

$$M_{n (monofunctional chain)}=18.99+7.93\times\left( 166.01 \right)+215.12=1550.57 g/mol$$

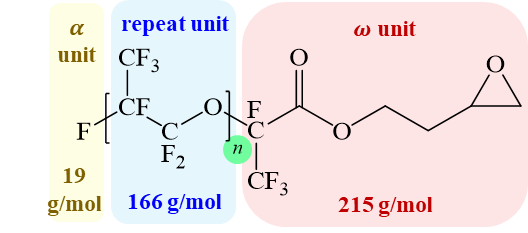


$M_{n (difunctional chain)}=$mass of $\alpha$ unit + $2\times$ $n\times$ (mass of repeat unit) + $2\times$mass of $\omega$ unit

$$M_{n (difunctional chain)}=332.04+2\times7.93\times\left( 166.01 \right)+2\times215.12=3395.20 g/mol$$

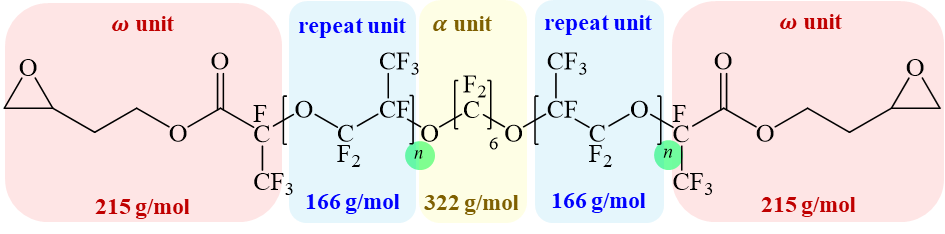


# S29. Mass spectrum of PFPAE-EO

GC–MS (EI) fragmentation: m/z = CHOCH_2_^+^ (43 m/z), CH_2_CHOCH_2_^+^ (57 m/z), CF_3_^+^ (69 m/z), CH_2_CH_2_CHOCH_2_^+^ (71 m/z), OCH_2_CH_2_CHOCH_2_^+^ (87 m/z), C_2_F_4_^+^ (100 m/z), C_2_F_5_^+^ (119 m/z), C_3_F_5_O^+^ (147 m/z), C_3_F_6_^+^ (150 m/z), C_3_F_7_^+^ (169 m/z), CF(CF_3_)C(O)OCH_2_CH_2_CHOCH_2_^+^ (215 m/z).

**
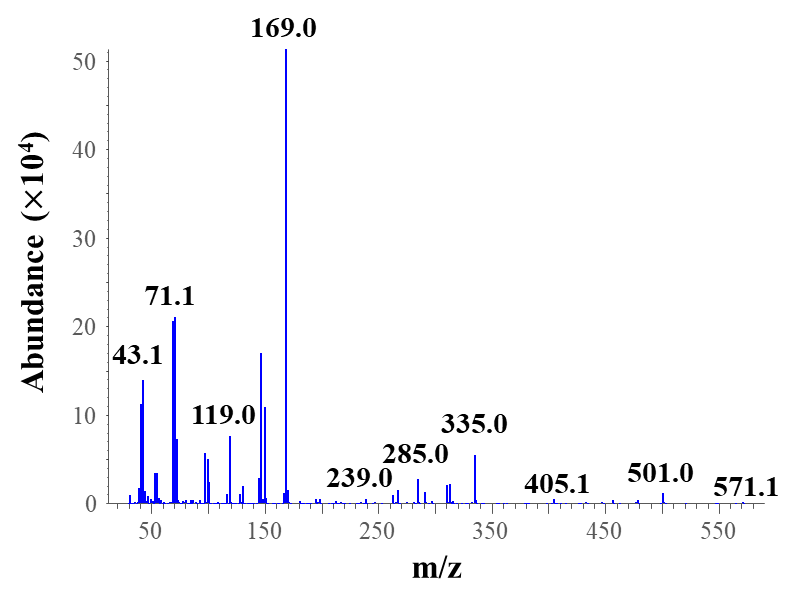
**

# S30. **^1^H-NMR (400 MHz, benzene-*d_6_*, 25 °C) spectrum of PFPAE-PO**

𝛿 = 4.69 (*m*, -OC***H_2_***-CH_2_-, 2H), 3.06 (*q,* -C***H***(O)CH_2_, 1H), 2.89 (*t,* -CH(O)C***H_c_***H_d_, 1H), 2.61 (*q,* -CH(O)CH_c_***H_d_***, 1H), 2.16 (*m,* -OCH_2_C***H***_2_CH_2_-, 2H), 2.04 (*m,* -C***H_f_***H_g_-CH(O)CH_2_, 1H), 1.68 (*m,* -CH_f_***H_g_***-CH(O)CH_2_, 1H). Impurities at 6.32-6.19 ppm are attributable to HFPO oligomers having as end group H-CF_2_-i.e. hydrogen end capped HFPO (HFPO HEC)


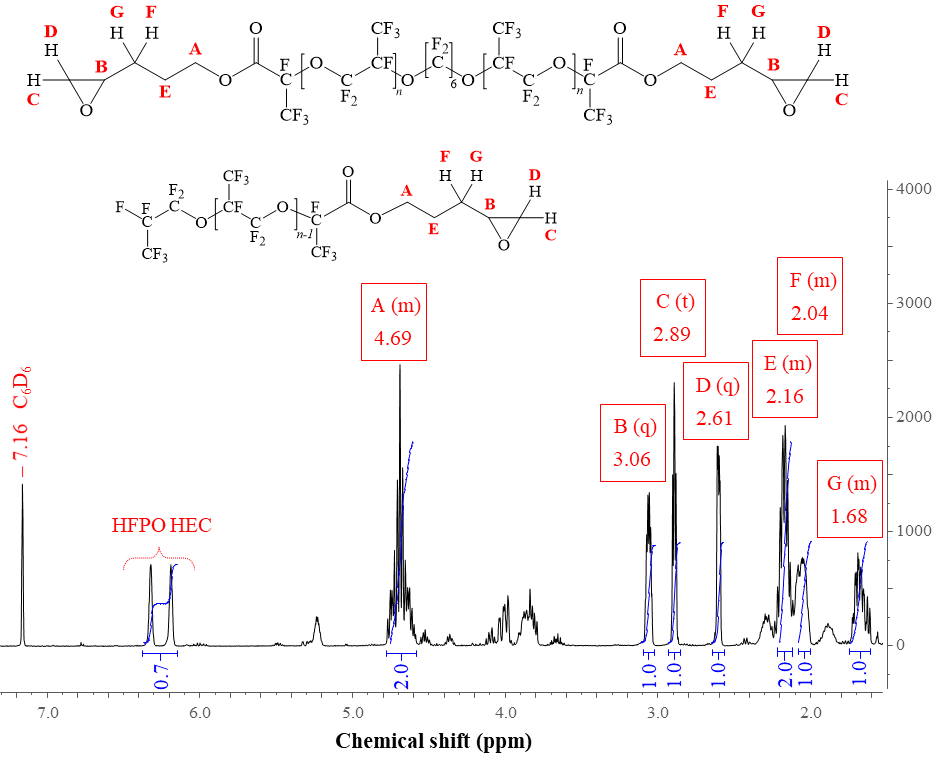


# S31. ^13^C-NMR (101 MHz, benzene-*d_6_*, 25 ^o^C) spectrum of PFPAE-PO

𝛿 = 158.76 (*d*, -***C***=O-), from 122.51 to 95.52 (*m*, −***C***F_3_ and ***C***F_2_ of repeat unit), 68.14 (*s*, -O***C***H_2_CH_2_CH_2_-), 50.61 (*s*, -***C***H(O)CH_2_), 45.94 (*s*, -CH(O)***C***H_2_), 28.86 (*s*, -OCH_2_CH_2_***C***H_2_-), 25.35 (*s*, -OCH_2_***C***H_2_CH_2_-).


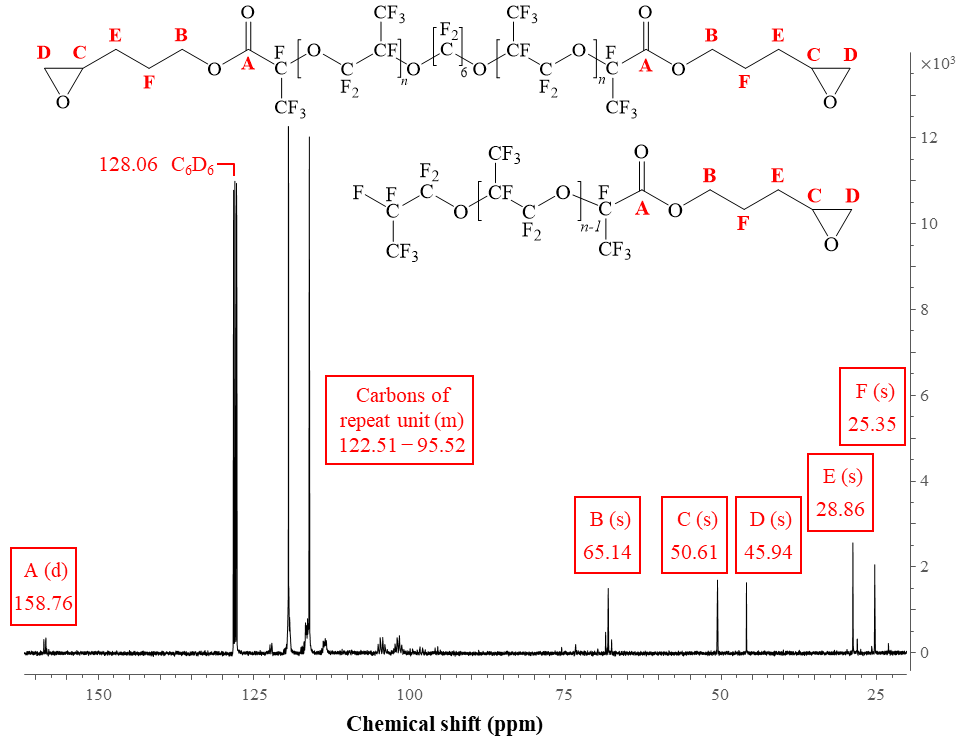


# S32. ^19^F-NMR (376.5 MHz, benzene-*d_6_*, 25 °C) spectrum of PFPAE-PO

𝛿 = -146.38 (*s*, HFPO II hydrogen end cap), -145.21 (*q*, C***F***(CF_3_) of repeat unit, mono and difunctional), -131.90 (*ω* C***F***(CF_3_), mono and difunctional), -130.26 (*s*, α C***F_2,_*** monofunctional), -125.70 (*s*, -CF_2_C***F_2_***CF_2_O-***_,_*** difunctional), -122.69 (*s*, -C***F_2_***CF_2_CF_2_O-***_,_*** difunctional), from -86.95 to -79.14 (CF_3_ and CF_2_ of repeat unit).


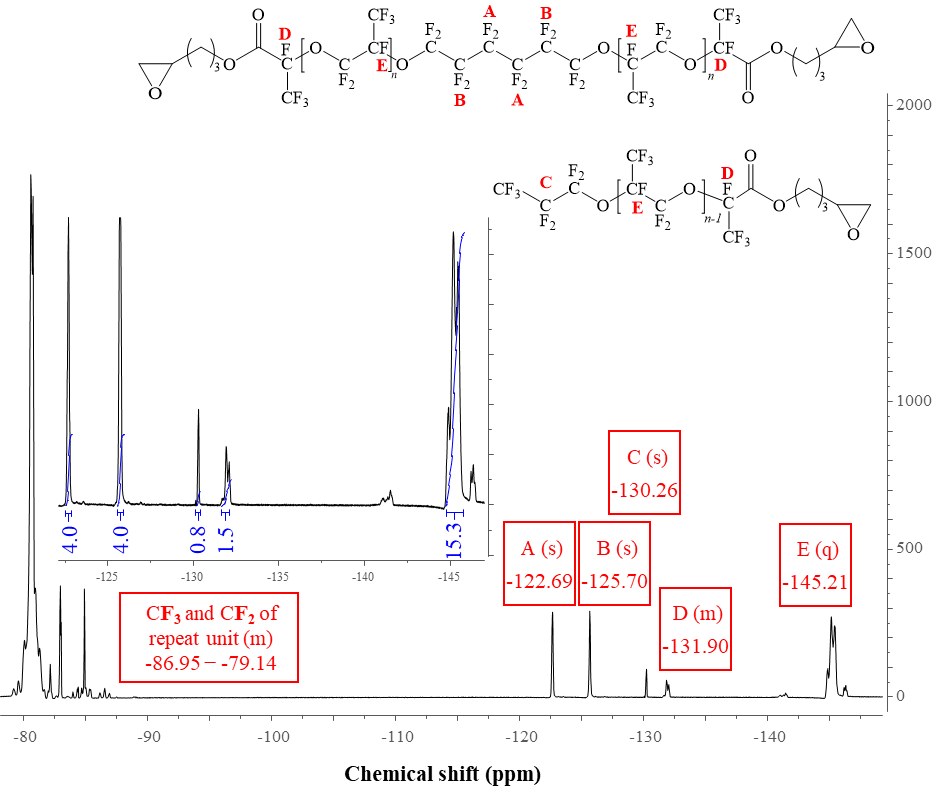


# S33. PFPAE-PO: Calculation of the percentages of the monofunctional and difunctional oligomers and their molecular weight

$$\text{\% di-PFPAE-PO}=\frac{\frac{{\int_{-131.90} \mathrm{CF}}}{{\int_{-130.26} \mathrm{CF}}_{2}}-\frac{1}{2}}{\frac{{\int_{-131.90} \mathrm{CF}}}{{\int_{-130.26} \mathrm{CF}}_{2}}+\frac{1}{2}}\times100=\frac{\frac{1.46}{0.79}-\frac{1}{2}}{\frac{1.46}{0.79}+\frac{1}{2}}\times100=57.41\%$$

$$f=\frac{\sum f_{i} {\%}_{i}}{\sum{\%}_{i}}=\frac{\left( 2\times57.41 \right)+\left( 1\times\left( 100-57.41 \right) \right)}{100}=1.58$$

$$n=\frac{{\int_{-145.25} \mathrm{CF}}}{{\int_{-132.01} \mathrm{CF}}}=\frac{15.34}{1.46}=10.51$$

$$M_{n (monofunctional chain)}=mass of \alpha unit + n\times(mass of repeat unit) + mass of \omega\mathrm{unit}$$

$$M_{n (monofunctional chain)}=18.99+10.51\times\left( 166.01 \right)+229.15=1992.90 g/mol$$

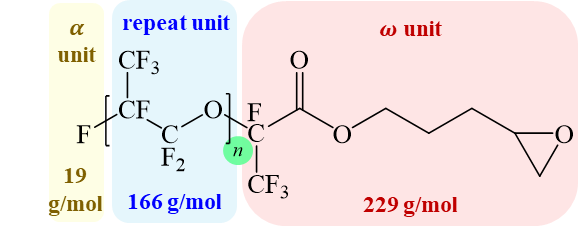


$M_{n (difunctional chain)}=$mass of $\alpha$ unit + $2\times$ $n\times$ (mass of repeat unit) + $2\times$mass of $\omega$ unit

$$M_{n (difunctional chain)}=332.04+2\times10.51\times\left( 166.01 \right)+2\times229.15=4279.87 g/mol$$

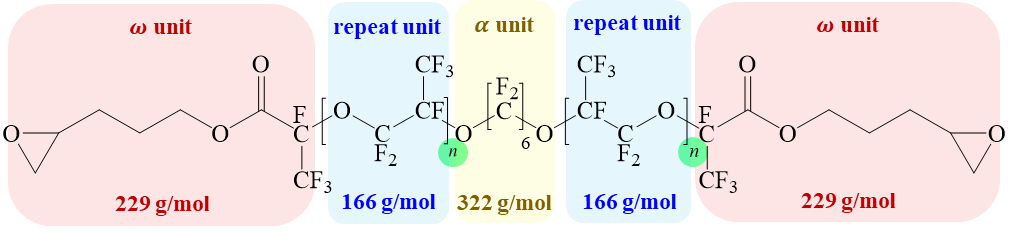


# S34. Mass spectrum of PFPAE-PO

GC–MS (EI) fragmentation: m/z = CHOCH_2_^+^ (43 m/z), CH_2_CHOCH_2_^+^ (57 m/z), CF_3_^+^ (69 m/z), CH_2_CH_2_CHOCH_2_^+^ (71 m/z), CH_2_CH_2_CH_2_CHOCH_2_^+^ (85 m/z), C_2_F_4_^+^ (100 m/z), OCH_2_CH_2_CH_2_CHOCH_2_^+^ (101 m/z), C_2_F_5_^+^ (119 m/z), C_3_F_5_O^+^ (147 m/z), C_3_F_6_^+^ (150 m/z), C_3_F_7_^+^ (169 m/z), CF(CF_3_)C(O)OCH_2_CH_2_CH_2_CHOCH_2_^+^ (229 m/z).


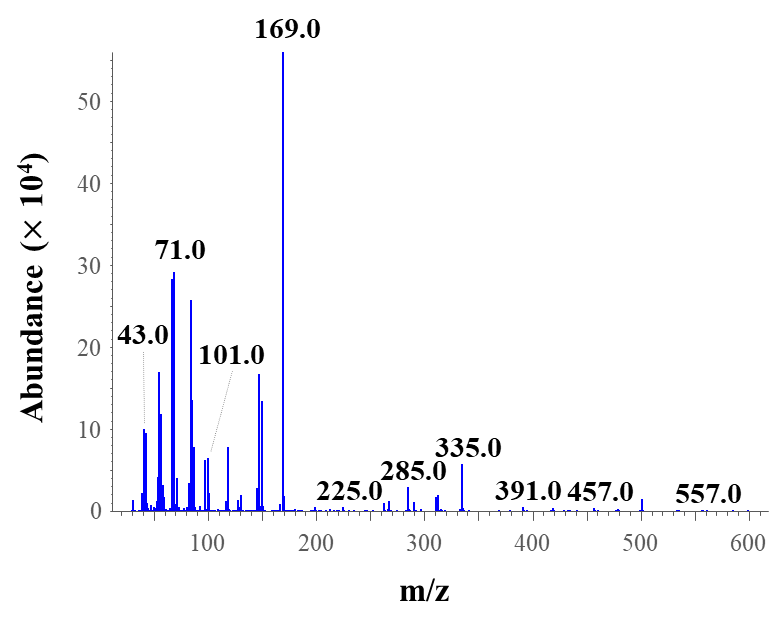


# S35. Preparation of the photocurable sample **PFPAE-BGVE**

From the PFPAE-BGVE NMR spectra, the difunctional content was found much higher than the other products and the average functionality was slightly higher than 1.9, therefore higher than the other products. Therefore, it was decided to synthesize a monofunctional monomer to reduce the functionality of the product to be used in photopolymerization. The synthesis and characterization of the monofunctional oligomer is reported below. Given the Mn of the monofunctional monomer (obtained from the ^19^F-NMR), the sample for the photopolymerization tests was prepared as follows: for each 1 mmol of PFPAE-BGVE (f=1.88, Mn medio =3216 g/mol), 0.8 mmol of mono BGVE (f=1, Mn =1355 g/mol) were added. Therefore, an average functionality of 1.6 was obtained.

**Synthesis of the monofunctional PFPAE-BGVE**

A solution of PFPAE acyl fluoride (1 equiv., M_n_ = 1380 g/mol, 1.45 mmol, 1 mL) in 5 mL of dried PFB was added to a 100 mL 3-necked round-bottomed flask and cooled at 0 °C. A mixture of triethylamine (1.1 equiv., 1.593 mmol, 0.221 mL), DMAP (3 wt%, 0.087 mmol, 0.01 g), and 1,4 butylene glycol vinyl ether (2 equiv., 1.593 mmol, 0.197 mL) in 25 mL of dry 40:60 DCM:PFB solution, was added, dropwise, to the PFPAE acyl fluoride/PFB mixture, at 0 °C, under continuous stirring. After 30 minutes, the ice bath was removed, and the reaction mixture was let to stir at room temperature during 12 h.

The reaction was followed by thin layer chromatography (TLC): powdered iodine was used as TLC stain and a 5:95 EtOAc/pentane solution as eluent. The conversion of the acyl fluoride into the corresponding vinyl ether was checked by the appearance of an orange-brown spot on light yellow background, located between the baseline of the TLC plate and the eluent front. The reaction was completed after 12 hours, as suggested by gas chromatography–mass spectrometry (GC–MS). Then, the reaction mixture was purified by flash chromatography (5:95 EtOAc/pentane solution used as eluent). After vacuum purging, a transparent oil was obtained as pure product (yield: 88%).

**Scheme S1** Synthetic paths followed to obtain the monofunctional PFPAE-BGVE.

**^1^H-NMR (400 MHz, benzene-d6 capillary, 25 °C) spectrum of monofunctional PFPAE-BGVE**

𝛿 = 6.60 (*dd*, -OC***H***CH_2_, ^3^*J_cis_* = 14.4 Hz, ^3^*J_trans_* = 6.8 Hz, 1H), 4.63 (*m*, -C(O)OC***H_2_***CH_2_-, 2H), 4.29 (*d,* ^3^*J*_cis_ = 14.4 Hz, -OCH*=*C***H_trans_***H_cis_, 1H), 4.13 (*d,* ^3^*J*_cis_ *=* 6.8 Hz, -OCH*=*CH_trans_***H_cis_***, 1H), 3.89 (*t*, -C(O)OCH_2_CH_2_CH_2_C***H_2_***-, 2H), 2.09 (*m*, -C(O)OCH_2_C***H_2_***CH_2_CH_2_-, 2H), 1.96 (*m*, -C(O)OCH_2_CH_2_C***H_2_***CH_2_-, 2H).


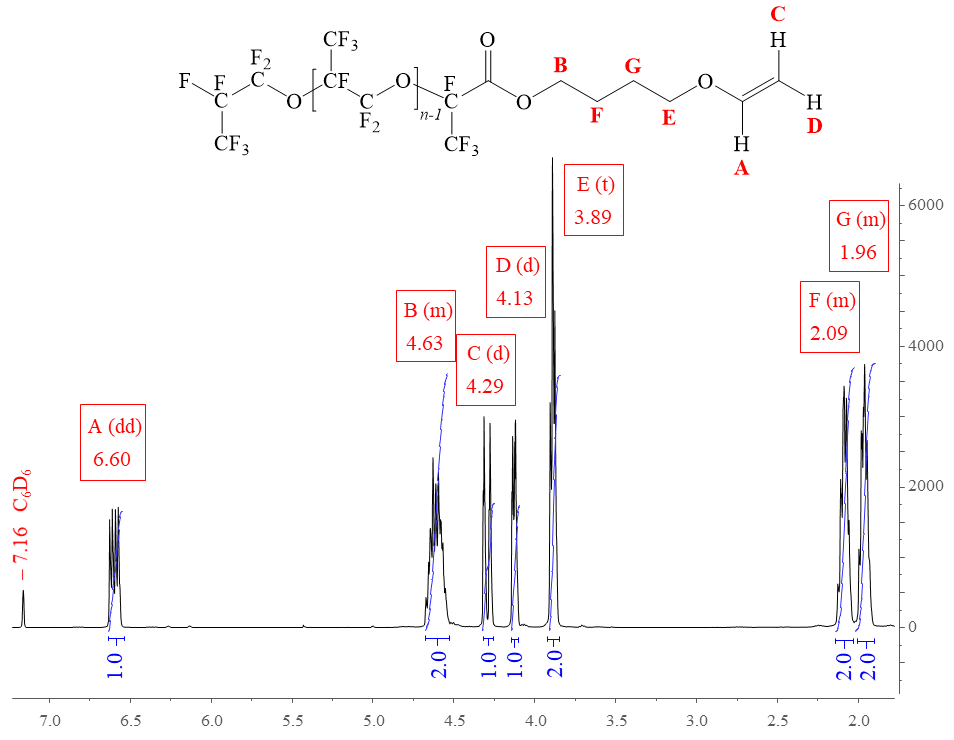


**^13^C-NMR (101 MHz, benzene-*d_6_*, 25 ^o^C) spectrum of monofunctional PFPAE-BGVE**

𝛿 = 158.75 (*d,* -***C***=O-), 151.65 (*s*, -O***C***HCH_2_), 122.91–9.17 (*m,* carbons of repeat unit), 85.90 (*s,* -OCH***C***H_2_), 68.22 (*s,* -***C***H_2_OCHCH_2_), 66.27 (*s*, -***C***H_2_CH_2_CH_2_CH_2_OCHCH_2_), 25.45 (*s*, -CH_2_CH_2_***C***H_2_CH_2_OCHCH_2_), 25.33 (*s*, -CH_2_***C***H_2_CH_2_CH_2_OCHCH_2_).


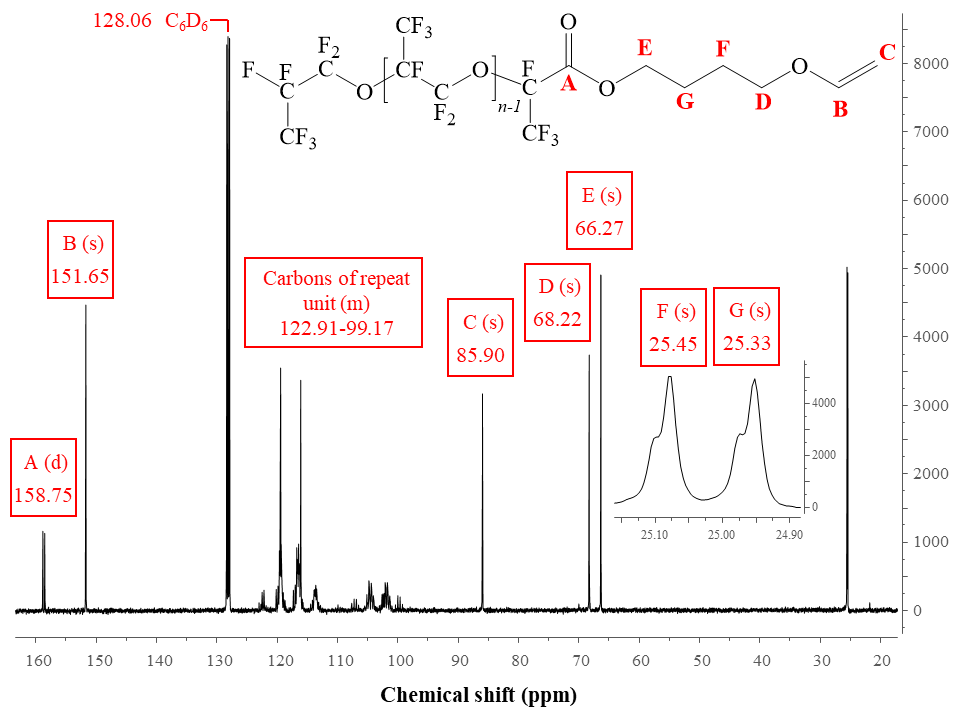


**^19^F-NMR (376.5 MHz, benzene-*d_6_*, 25 °C) spectrum of monofunctional PFPAE-BGVE**

𝛿 = -145.59 (*q*, C***F***(CF_3_) of repeat unit), -132.12 (*ω* C***F***(CF_3_)), -130.58 (*s*, α C***F_2_***), -85.60 to -79.40 (C***F_3_*** and C***F_2_*** of repeat unit).


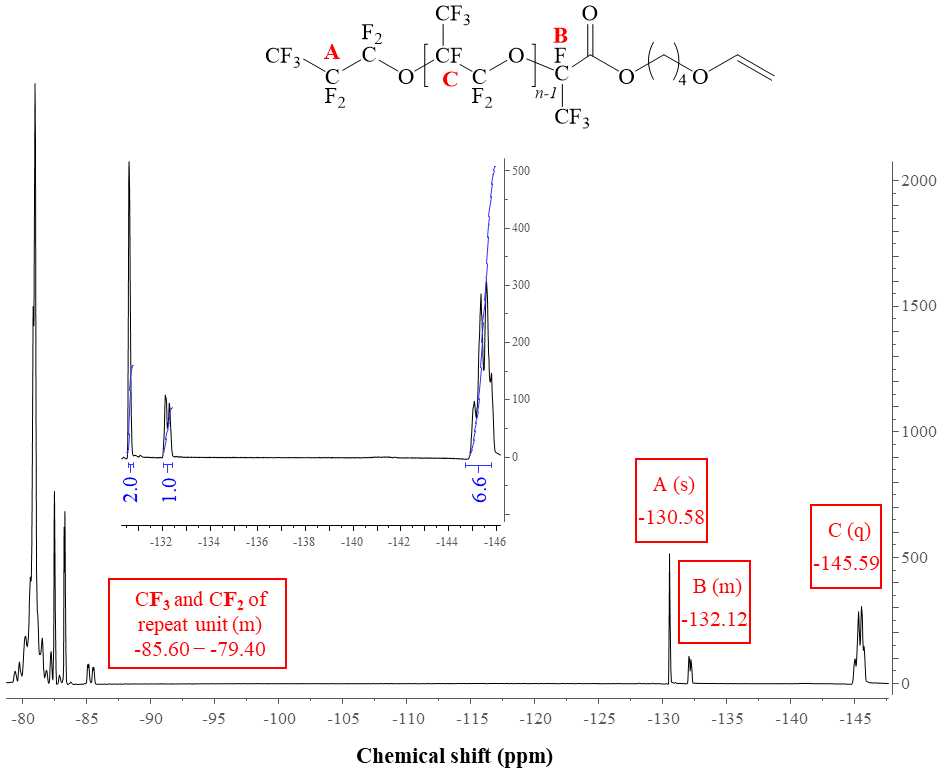


**Calculation of the molecular weight of the monofunctional PFPAE-BGVE**

$$n=\int_{-145.59} \mathrm{CF}=6.58$$

$M_{n}=$1354,52

**Mass spectrum of monofunctional PFPAE- BGVE**

GC–MS (EI) fragmentation: m/z = OCHCH_2_^+^ (43 m/z), CH_2_OCHCH_2_^+^ (57 m/z), CF_3_^+^ (69 m/z), CH_2_CH_2_OCHCH_2_^+^ (71 m/z), CH_2_CH_2_CH_2_OCHCH_2_^+^ (85 m/z), CH_2_CH_2_CH_2_CH_2_OCHCH_2_^+^ (99 m/z), C_2_F_4_^+^ (100 m/z), OCH_2_CH_2_CH_2_CH_2_OCHCH_2_^+^ (115 m/z), C_2_F_5_^+^ (119 m/z), C_3_F_5_O^+^ (147 m/z), C_3_F_6_^+^ (150 m/z), C_3_F_7_^+^ (169 m/z), CF(CF_3_)C(O)OCH_2_CH_2_CH_2_CH_2_OCHCH_2_^+^ (243 m/z).


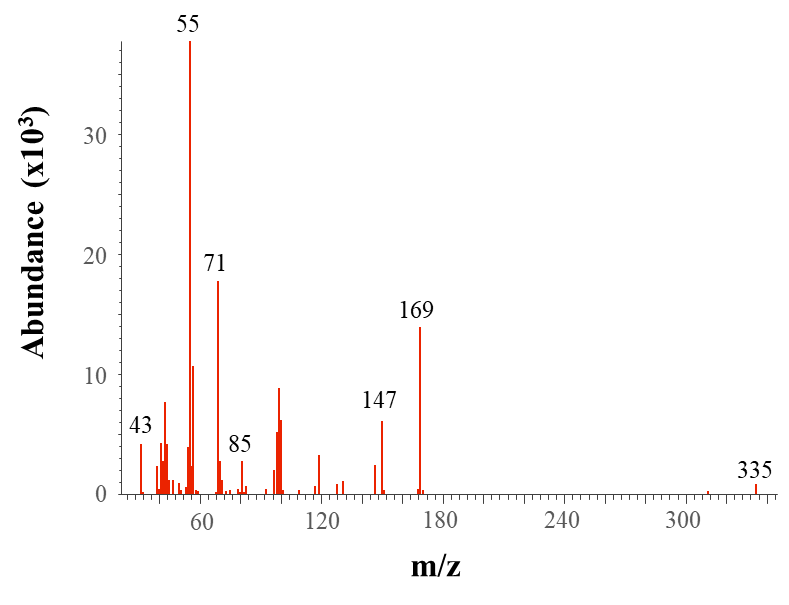


# S36. Study of the polymerization kinetics by photo-DSC


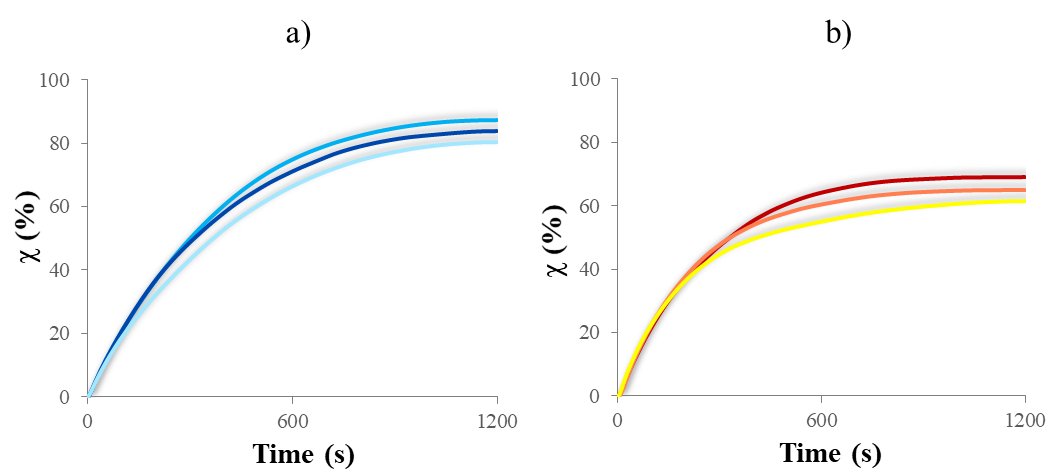


a) **–** PFPAE-EGVE, **–** PFPAE-BGVE, **–** PFPAE-DEGVE; b) ***–*** PFPAE-MO, **–** PFPAE-EO, **–** PFPAE-PO.

# S37. Maximum rate of polymerization of the photocured fluoropolymers by photo-DSC.

| Polymer | R_P_^MAX^ (% s^-1^) |
| --- | --- |
|  | Photo-DSC |
| PFPAE-EGVE | 0.46 |
| PFPAE-BDVE | 0.49 |
| PFPAE-DEGVE | 0.51 |
| PFPAE-MO | 0.32 |
| PFPAE-EO | 0.29 |
| PFPAE-PO | 0.27 |

# S38. Study of the photopolymerization reaction by ATR FT-IR


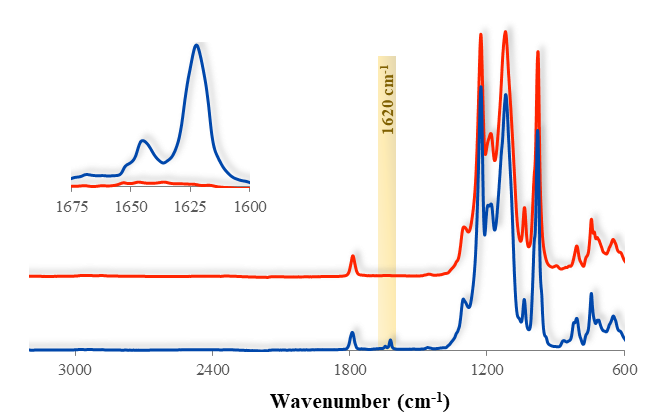


ATR FT-IR spectra of the PFPAE-EGVE polymer

before irradiation;
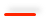
after irradiation + 48 h post-curing;


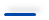


**R*_h_***: ~**1780** cm^-1^ **C=O** bond; ~**1620** cm^-1^ **C=C**; ~**1100** cm^-1^ **C-O-C ethers**;

**R*_f_*** : ~**1240** cm^-1^ stretching **C-F** bond, ~**1100** cm^-1^ **C-O-C ethers**;

**Photoinitiator**: ~**2950** cm^-1^ stretching C=**C-H**; ~**1600-1320** cm^-1^ **C_6_H_6_** bonds.


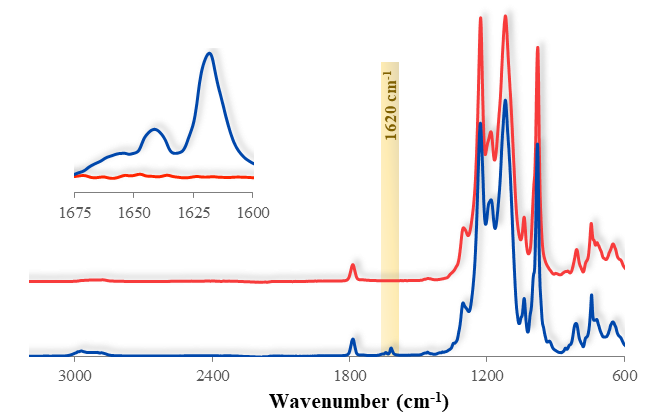


ATR FT-IR spectra of the PFPAE-BGVE polymer


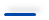
before irradiation;
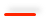
after irradiation + 48 h post-curing;

**R*_h_***: ~**1780** cm^-1^ **C=O** bond; ~**1620** cm^-1^ **C=C**; ~**1100** cm^-1^ **C-O-C ethers**;

**R*_f_*** : ~**1240** cm^-1^ stretching **C-F** bond, ~**1100** cm^-1^ **C-O-C ethers**;

**Photoinitiator**: ~**2950** cm^-1^ stretching C=**C-H**; ~**1600-1320** cm^-1^ **C_6_H_6_** bonds.


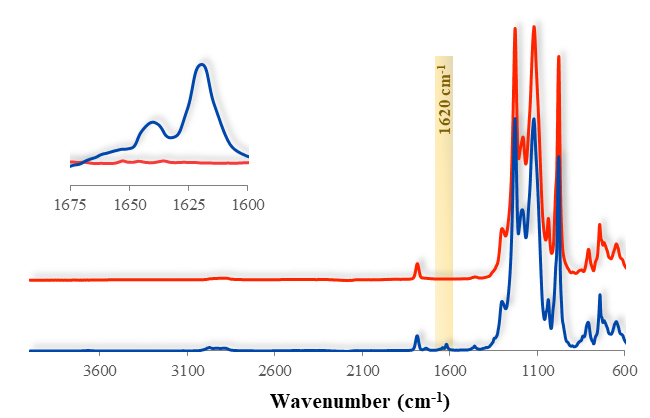


ATR FT-IR spectra of the PFPAE-DEGVE polymer


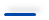
before irradiation;
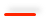
after irradiation + 48 h post-curing;

**R*_h_***: peak ~**1780** cm^-1^ **C=O** bond; ~**1620** cm^-1^ **C=C**; peak ~**1100** cm^-1^ **C-O-C ethers**;

**R*_f_*** : ~**1240** cm^-1^ stretching **C-F** bond, ~**1100** cm^-1^ **C-O-C ethers**;

**Photoinitiator**: ~**2950** cm^-1^ stretching C=**C-H**; ~**1600-1320** cm^-1^ **C_6_H_6_** bonds.


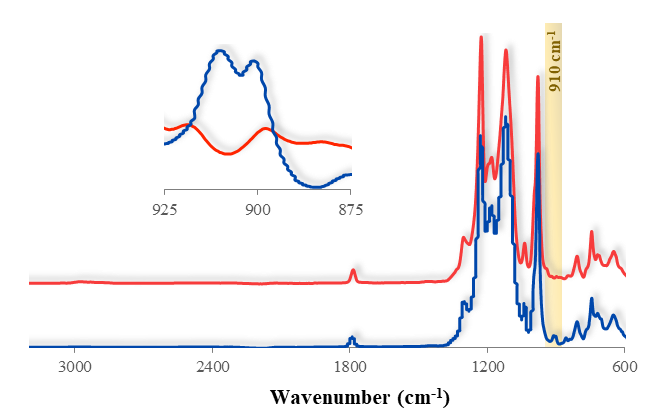


ATR FT-IR spectra of the PFPAE-MO polymer


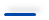
before irradiation;
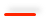
after irradiation + 48 h post-curing;

**R*_h_***: ~**1780** cm^-1^ **C=O** bond; ~**1100** cm^-1^ **C-O-C ethers**; ~**910** cm^-1^ **epoxides**;

**R*_f_*** : ~**1240** cm^-1^ stretching **C-F** bond, ~**1100** cm^-1^ **C-O-C ethers**;

**Photoinitiator**: peak ~**2950** cm^-1^ stretching C=**C-H**; peak ~**1600-1320** cm^-1^ **C_6_H_6_** bonds.


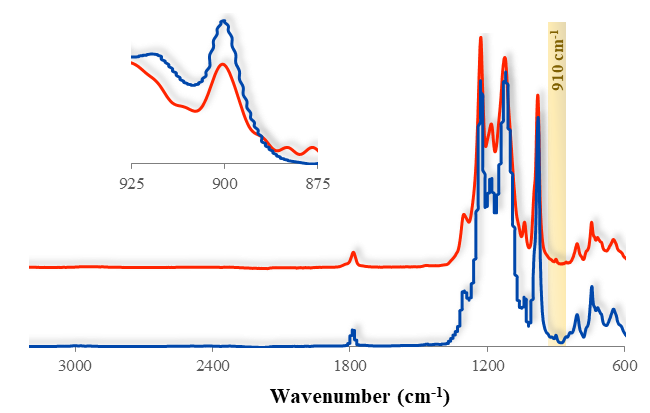


ATR FT-IR spectra of the PFPAE-EO polymer


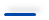
before irradiation;
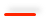
after irradiation + 48 h post-curing;

**R*_h_***: ~**1780** cm^-1^ **C=O** bond; ~**1100** cm^-1^ **C-O-C ethers**; ~**910** cm^-1^ **epoxides**;

**R*_f_*** : ~**1240** cm^-1^ stretching **C-F** bond, ~**1100** cm^-1^ **C-O-C ethers**;

**Photoinitiator**: ~**2950** cm^-1^ stretching C=**C-H**; ~**1600-1320** cm^-1^ **C_6_H_6_** bonds.


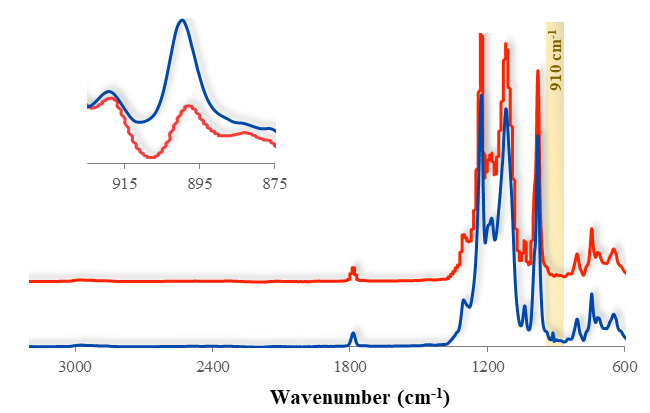


ATR FT-IR spectra of the PFPAE-PO polymer


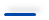
before irradiation;
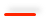
after irradiation + 48 h post-curing;

**R*_h_***: ~**1780** cm^-1^ **C=O** ; ~**1100** cm^-1^ **C-O-C ethers**; ~**910** cm^-1^ **epoxides**;

**R*_f_*** : ~**1240** cm^-1^ stretching **C-F** , ~**1100** cm^-1^ **C-O-C ethers**;

**Photoinitiator**: ~**2950** cm^-1^ stretching C=**C-H**; peak ~**1600-1320** cm^-1^ **C_6_H_6_**.

# S39. TGA: degradation temperatures of the UV-cured fluoropolymers

| Homopolymer | T_onset_  (°C) | T_max1_  (°C) | T_max2_ $\sim$ T_90%_  (°C) |
| --- | --- | --- | --- |
| PFPAE-EGVE | 126 | 290 | 457 |
| PFPAE-BGVE | 131 | 304 | 523 |
| PFPAE-DEGVE | 138 | 299 | 544 |
| PFPAE-MO | 137 | 326 | 524 |
| PFPAE-EO | 129 | 304 | 534 |
| PFPAE-PO | 119 | 293 | 532 |

# S40. Water contact angle hysteresis measurements, on the air side, of the UV-cured polymers

1. **–** PFPAE-EGVE, **–** PFPAE-BGVE, **–** PFPAE-DEGVE;
2. **–** PFPAE-MO, **–** PFPAE-EO, **–** PFPAE-PO.


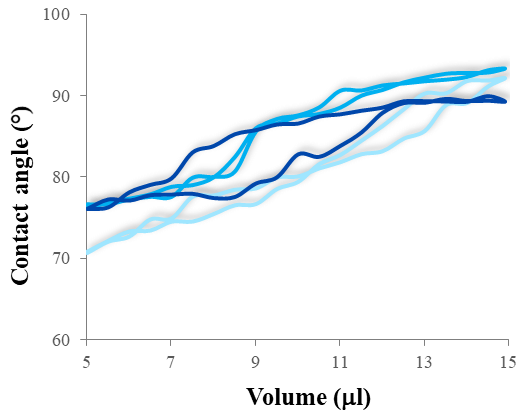
a)
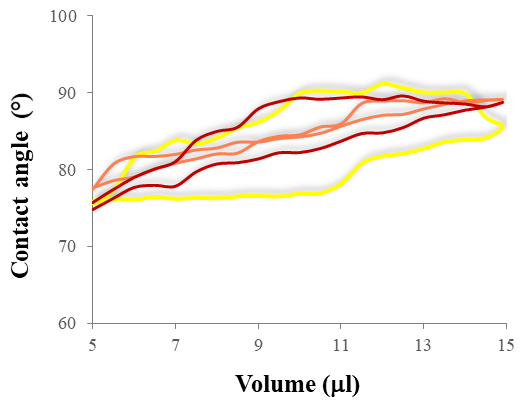
b)
